# Diving dinosaurs? Caveats on the use of bone compactness and pFDA for inferring lifestyle

**DOI:** 10.1371/journal.pone.0298957

**Published:** 2024-03-06

**Authors:** Nathan P. Myhrvold, Stephanie L. Baumgart, Daniel Vidal, Frank E. Fish, Donald M. Henderson, Evan T. Saitta, Paul C. Sereno

**Affiliations:** 1 Intellectual Ventures, Bellevue, Washington, United States of America; 2 Department of Organismal Biology and Anatomy, University of Chicago, Chicago, Illinois, United States of America; 3 Department of Physiological Sciences, College of Veterinary Medicine, University of Florida, Gainesville, Florida, United States of America; 4 Facultad de Ciencias, Departamento de Física Matemática y de Fluidos, Grupo de Biología Evolutiva, UNED, Madrid, Madrid, Spain; 5 Department of Biology, West Chester University, West Chester, Pennsylvania, United States of America; 6 Royal Tyrrell Museum of Palaeontology, Drumheller, Alberta, Canada; 7 Committee on Evolutionary Biology, University of Chicago, Chicago, Illinois, United States of America; Chinese Academy of Sciences, CHINA

## Abstract

The lifestyle of spinosaurid dinosaurs has been a topic of lively debate ever since the unveiling of important new skeletal parts for *Spinosaurus aegyptiacus* in 2014 and 2020. Disparate lifestyles for this taxon have been proposed in the literature; some have argued that it was semiaquatic to varying degrees, hunting fish from the margins of water bodies, or perhaps while wading or swimming on the surface; others suggest that it was a fully aquatic underwater pursuit predator. The various proposals are based on equally disparate lines of evidence. A recent study by Fabbri and coworkers sought to resolve this matter by applying the statistical method of phylogenetic flexible discriminant analysis to femur and rib bone diameters and a bone microanatomy metric called global bone compactness. From their statistical analyses of datasets based on a wide range of extant and extinct taxa, they concluded that two spinosaurid dinosaurs (*S*. *aegyptiacus*, *Baryonyx walkeri*) were fully submerged “subaqueous foragers,” whereas a third spinosaurid (*Suchomimus tenerensis*) remained a terrestrial predator. We performed a thorough reexamination of the datasets, analyses, and methodological assumptions on which those conclusions were based, which reveals substantial problems in each of these areas. In the datasets of exemplar taxa, we found unsupported categorization of taxon lifestyle, inconsistent inclusion and exclusion of taxa, and inappropriate choice of taxa and independent variables. We also explored the effects of uncontrolled sources of variation in estimates of bone compactness that arise from biological factors and measurement error. We found that the ability to draw quantitative conclusions is limited when taxa are represented by single data points with potentially large intrinsic variability. The results of our analysis of the statistical method show that it has low accuracy when applied to these datasets and that the data distributions do not meet fundamental assumptions of the method. These findings not only invalidate the conclusions of the particular analysis of Fabbri *et al*. but also have important implications for future quantitative uses of bone compactness and discriminant analysis in paleontology.

## Introduction

### Spinosaurids discovery

Spinosaurids are Cretaceous-era therapods known for their enormous size, their long, narrow skulls, and the dorsal sails that exemplify *Spinosaurus* and some other genera. When Stromer first described *Spinosaurus aegyptiacus* in 1915 from Upper Cretaceous outcrops in Egypt’s Western Desert, he highlighted the spaced, conical teeth and elongate jaws as crocodile-like adaptations for a piscivorous diet [1]. Similar dietary inferences were made some 70 years later in initial descriptions of two older, closely related spinosaurids, *Baryonyx walkeri* [2] and *Suchomimus tenerensis* [3], from Lower Cretaceous outcrops in England and Niger, respectively. Although in recent years remains of other spinosaurids have been discovered, they do not impact the arguments we address regarding the three aforementioned well-known spinosaurids. The following overview of *Spinosaurus* lifestyle inference is not intended to be a complete or thorough review of all arguments or scientific contributions to the topic but instead focuses on key points relevant to the current study.

### Spinosaurus lifestyle inference

In describing *Baryonyx* in more detail, Charig and Milner outlined what may be termed a shallow-water opportunist lifestyle [4]. Although they considered fish an important dietary component, skeletal features plausibly related to functionality in water deserving of semiaquatic status were absent:

On balance, we still envisage *Baryonyx* as mainly a fish-eater. It probably crouched on the banks of lakes, creeks and rivers or waded in the shallows (Frontispiece), and it secured its prey by direct seizure with the jaws and perhaps also by ‘gaffing’. Small fishes would have been swallowed whole, larger ones broken up by the powerful fore-limbs with their huge claws. Fishing, however, was not the only source of food…. If we accept that fish formed a significant part of the diet of *Baryonyx*, then we must consider the possibility that the animal led an aquatic or semi-aquatic existence. Nevertheless, its anatomy gives no indication of any modifications towards that mode of life.

In 2014, Ibrahim *et al*. introduced the notion of a “semiaquatic” *Spinosaurus aegyptiacus*, with a lifestyle tied more closely to the water’s edge, on the basis of a partial skeleton from Upper Cretaceous rocks in Morocco [5]:

We describe adaptations for a semiaquatic lifestyle in the dinosaur *Spinosaurus aegyptiacus*…. These adaptations suggest that *Spinosaurus* was primarily a piscivore, subsisting on sharks, sawfish, coelacanths, lungfish, and actinopterygians that were common in the Kem Kem river system.

These authors noted the downsized, retracted external nares and several unusual postcranial features, which they viewed as enhancing predation while wading and surface swimming using “foot-powered paddling” and “lateral undulation of the tail.” These features, which included reduced pelvic girdle and hind limbs, solid long bones, long pedal digit I, flattened pedal unguals, and reduced caudal articulations, would have limited terrestrial agility. These authors asserted that *Spinosaurus* “must have been an obligate quadruped on land,” based on their calculation of the location of the center of mass anterior to the hips. However, that calculation (made by PCS, one of the current authors) has since been recognized to have erroneously shifted the center of mass forward from the hip by the addition, rather than the subtraction, of the estimated volume of internal air space.

The suite of postcranial features in *Spinosaurus* alluded to above nonetheless clearly distinguish it from the baryonychines *Baryonyx* and *Suchomimus* and from other terrestrial nonavian theropods [5,6]. Ecologically, *Spinosaurus* was envisioned as a semiaquatic piscivorous predator frequenting both land and water, capable of both walking and surface swimming, based on anatomical features in functional analogy to crocodiles, shore birds, and semiaquatic mammals, with the dorsal sail functioning as a “display structure that would have remained visible while swimming” [5].

This more complete view of the skeleton renewed interest in *Spinosaurus*, prompting a series of papers on its lifestyle and functional capacities in water. In 2016, Gimsa *et al*. argued that the dorsal sail may have played an important role in fully submerged underwater swimming and active pursuit of prey [7]. In 2017, Hone and Holtz summarized various viewpoints on spinosaurid diet, function, and habitat preference, arguing for a predominantly piscivorous diet and semiaquatic lifestyle while not ruling out scavenging on land or the use of their forelimbs to “dig for buried” prey [6].

In 2018, Henderson created 3-D models of *Suchomimus* and *Spinosaurus* to examine both terrestrial locomotion and buoyancy [8]. For *Spinosaurus* he estimated that the terrestrial center of mass would have been located in the hip region over the hind limbs, suggesting a bipedal stance similar to *Suchomimus*. With respect to swimming, Henderson’s calculations led him to conclude that *Spinosaurus* could float but was laterally unstable and tended to tip over. Buoyancy from air sacs within the axial column rendered *Spinosaurus* “unsinkable”—the force necessary to submerge the body fully in a dive would be more than the animal could reasonably generate. Henderson concluded that these factors make the swimming locomotion proposed in 2014 by Ibrahim *et al*. [5] implausible. He instead proposed that *Spinosaurus* was more likely identified as following the model put forth by Charig and Miller for *Baryonyx*, summarized in the quote above. In particular, Henderson proposed that *Spinosaurus* could “procure aquatic prey without having to become fully immersed” [8], akin to “gaffing” with forelimb claws as proposed by Charig and Miller [4], in analogy to the ambush predation of fish by grizzly bears [8].

In 2019, Arden *et al*. [9] proposed that spinosaurids were “highly specialized semiaquatic predators” on the basis of cranial features, in particular the position of external nares and orbits. Narial retraction in spinosaurids, however, provides no clear association with an open water aquatic lifestyle, when examined in the light of comparative cranial measurements [10]. Orbital position in spinosaurids, likewise, is similar to that seen in terrestrial nonavian theropods [10].

Then in 2020, the discovery of the high-spined tail of the Moroccan skeleton inspired the aquatic pursuit predator hypothesis, described by Ibrahim *et al*. [11]:

Contrary to recent suggestions^10^ that *Spinosaurus* was confined to wading and the apprehension of prey from around the edges of bodies of water, the morphology and function of its tail—along with its other adaptations for life in water^7^—point to *Spinosaurus* having been an active and highly specialized aquatic predator that pursued and caught its prey in the water column (S7 Fig).

Here Ibrahim *et al*. cite Henderson’s 2018 paper ([8], their ref. [10]), which they reject without directly challenging its methodology or results. Instead, Ibrahim *et al*. rested their conclusions entirely on (1) qualitative anatomical analysis of the new tail specimen and (2) a series of experiments in which 2-D plastic models of several different tail shapes were moved robotically in a water tank. In figures and video of the experiments, the *Spinosaurus* tail-shape model is shown submerged with its sagittal plane vertical and long axis parallel to the water surface and tank bottom [11, Fig 3A, Supplementary Information]. Although Ibrahim *et al*. did not directly specify swimming depth or diving behavior, their experimental tail model was submerged to a depth equal to or greater than half the length of the tail, which they reconstructed with a length of approximately 10 m. *Spinosaurus* thus would have been swimming with its center line at least 5 m below the surface—deep enough for full submergence, including its dorsal sail. In their S7 Fig, they depicted a “swimming pose” of *Spinosaurus* inclined upward at approximately 45 degrees, as if it were swimming toward the surface after a deep dive “in the water column,” as they described in the above quotation. *Spinosaurus*, in their view, was not limited to surface swimming, which they never modeled. Although they never used the words “dive” or “diving” in the paper, there is little other means to fully submerge and pursue prey in the water column.

Ibrahim *et al*. distinguished their 2020 findings [11], which emphasized active pursuit predation in the water column, from the earlier studies [5,6, and others] that proposed what they termed a “partially aquatic, piscivorous mode of life” [11]. In their view, the fully aquatic pursuit predator hypothesis ranked as novel, a hypothesis we agree is distinctively aquatic in interpretation. More recently, Gimsa and Gimsa interpreted their small-scale model results similarly, as supporting their previous hypothesis that “*Spinosaurus* was a capable swimmer with the dorsal sail serving hydrodynamic purposes during submerged swimming” [12]. The fully aquatic pursuit predator hypothesis, nonetheless, was challenged in 2021 by Hone and Holtz by a range of qualitative comparisons and a quantitative comparison of overall skull shape [13]. They suggested that drag would have limited the swimming speed of *Spinosaurus* at the surface or underwater, concluding that the fully aquatic pursuit predator hypothesis is unlikely for a number of reasons [13]:

As a putative aquatic pursuit predator, *Spinosaurus* has issues with instability in water, high drag, the position of the eyes and nostrils, low swimming efficiency, strong neck ventriflexion, and isotopic signatures showing extended periods in terrestrial conditions and feeding on terrestrial animals, and there remain questions about its ability to swim and submerge effectively as a whole.

Their conclusion regarding *Spinosaurus* lifestyle was this [13]:

*Spinosaurus* is therefore best interpreted as shoreline generalist based on the available information. Capable of capturing both aquatic and terrestrial prey, and perhaps an opportunistic scavenger, adult *Spinosaurus* likely took aquatic prey by standing in shallow water or at the margins of water bodies.

That description of *Spinosaurus* echoed that of Charig and Milner regarding the lifestyle of *Baryonyx* quoted above [4]. Indeed, the “generalist” designation might apply equally to many large theropods. Finally, we note here that the terms “shore” and “coast” (or “shoreline” and “coastline”) usually connote land adjacent to an ocean or sea, whereas we do know from recent finds that *Spinosaurus* roamed far inland [14].

In 2022, Sereno and a group of coauthors (including most authors of the present study) published a study that began with accurate 3-D skeletal models of both *Spinosaurus* and *Suchomimus*, based on all available fossil materials [14]. After building a flesh model of *Spinosaurus* over its skeletal model, with body parts adjusted to estimated densities, they performed biomechanical tests for the various proposed functional hypotheses. The center of mass in their flesh model was found to be located above the acetabulum, supporting bipedal stance and bipedal locomotion on land.

Drag experienced during swimming was calculated, using an estimate of body surface area. Analysis of the surface area of the tail, feet, and hands was performed to enable quantitative estimation of the maximum propulsive thrust that could be generated by *Spinosaurus*, which was found to be quite modest compared to drag. They concluded that if *Spinosaurus* swam, it would have done so very slowly, achieving a maximum velocity of ~ 0.8 m/s in surface swimming and ~1.4 m/s if swimming at a depth of 10 m or more to avoid wave drag. They contrasted these with typical velocities of extant pursuit predators such as dolphins and orcas, which range from 10–33 m/s, and concluded that *Spinosaurus* was far too slow a swimmer to have relied primarily on pursuit predation of fish.

Sereno *et al*. also performed a stability analysis, which showed that *Spinosaurus* could wade if supported by its feet [14]. However, if it waded deep enough that it started to float (>2.6 m), torque of the dorsal sail would cause it to tip sideways, leaving it floating on its side with the waterline roughly parallel to the dorsoventral plane (Fig 3B of [14]). Righting itself would have been impossible, due to the severe limitations on transverse thrust generated by its limbs and tail, their inadequate propulsive force, and their location along the body. They concluded that *Spinosaurus* was at best ineffective as a surface swimmer (free of the substrate), but more likely could not swim at all.

In regards to diving, Sereno *et al*. [14] replicated the “unsinkable” finding of Henderson [8]. Their buoyancy analysis showed that *Spinosaurus* could not generate the thrust needed to counter buoyant forces and fully submerge its body; the estimated thrust from the limbs and tail was too small by a factor of 15 to 25, depending on the buoyancy model used. Nor could *Spinosaurus* remain submerged, even if it were positioned underwater. They concluded that *Spinosaurus* could not fully submerge to accomplish a dive.

These findings provide direct quantitative refutation of the aquatic pursuit predator hypothesis that describes active swimming, diving, and pursuit predation “in the water column.” They do not falsify arguments for predation while wading into water over 2 m in depth or hunting for, or scavenging, terrestrial prey. Sereno *et al*. described the lifestyle of *Spinosaurus* as a “semiaquatic bipedal ambush piscivore that frequented the margins of coastal and inland waterways” [14]. This description of *Spinosaurus* ecology overlaps with many, although perhaps not all, of the conclusions of previous studies [8,13].

### Bone compactness statistics as lifestyle arbiter

In 2022, Fabbri *et al*. used statistical analysis of bone compactness to classify spinosaurids and other dinosaurs with respect to underwater foraging habits [15]. They paired global bone compactness (*Cg*), defined as cross-sectional area covered by bone divided by total cross-sectional area, and maximum bone diameter (*MD*) with a relatively new statistical method called phylogenetic flexible discriminant analysis (pFDA). This method is described below in the statistical method implementations section of Materials and methods.

The goal was to classify carnivorous dinosaur taxa as either “subaqueous foragers” or not. Bypassing detailed studies of anatomy and biomechanics, their method offered the tantalizing possibility that a broad database including many taxa, each represented by a single (*MD*, *Cg*) datapoint, could yield statistical evidence that would directly reveal where dinosaurs foraged. From this analysis, they concluded that spinosaurids were “aquatic specialists” but with “surprising ecological disparity.” *Spinosaurus* and *Baryonyx*, they argued, made regular use of “subaqueous foraging” with “fully submerged behavior,” whereas *Suchomimus*, a close relative of *Baryonyx*, was a nondiving terrestrial predator restricted to wading in the shallows [15: 852].

The importance of this study is twofold. First, they outlined what appeared to be definitive evidence and a novel approach to determine lifestyle or habitat questions based on bone cross sections, with implications for interpreting the fossil record. Second, if this approach could successfully resolve such a thorny issue, then perhaps it, or approaches inspired by it, could be applied to other lifestyle or habitat questions in the fossil record.

### Study goals

The purpose of the present study is to reexamine the datasets and analytical techniques employed by Fabbri *et al*. to elucidate the foraging habits of spinosaurids, with an aim toward testing the validity for lifestyle inference of bone microanatomy metrics, such as *Cg*, and the use of discriminant analysis, in particular pFDA, as an appropriate statistical method for such inference.

**Fig 1:**
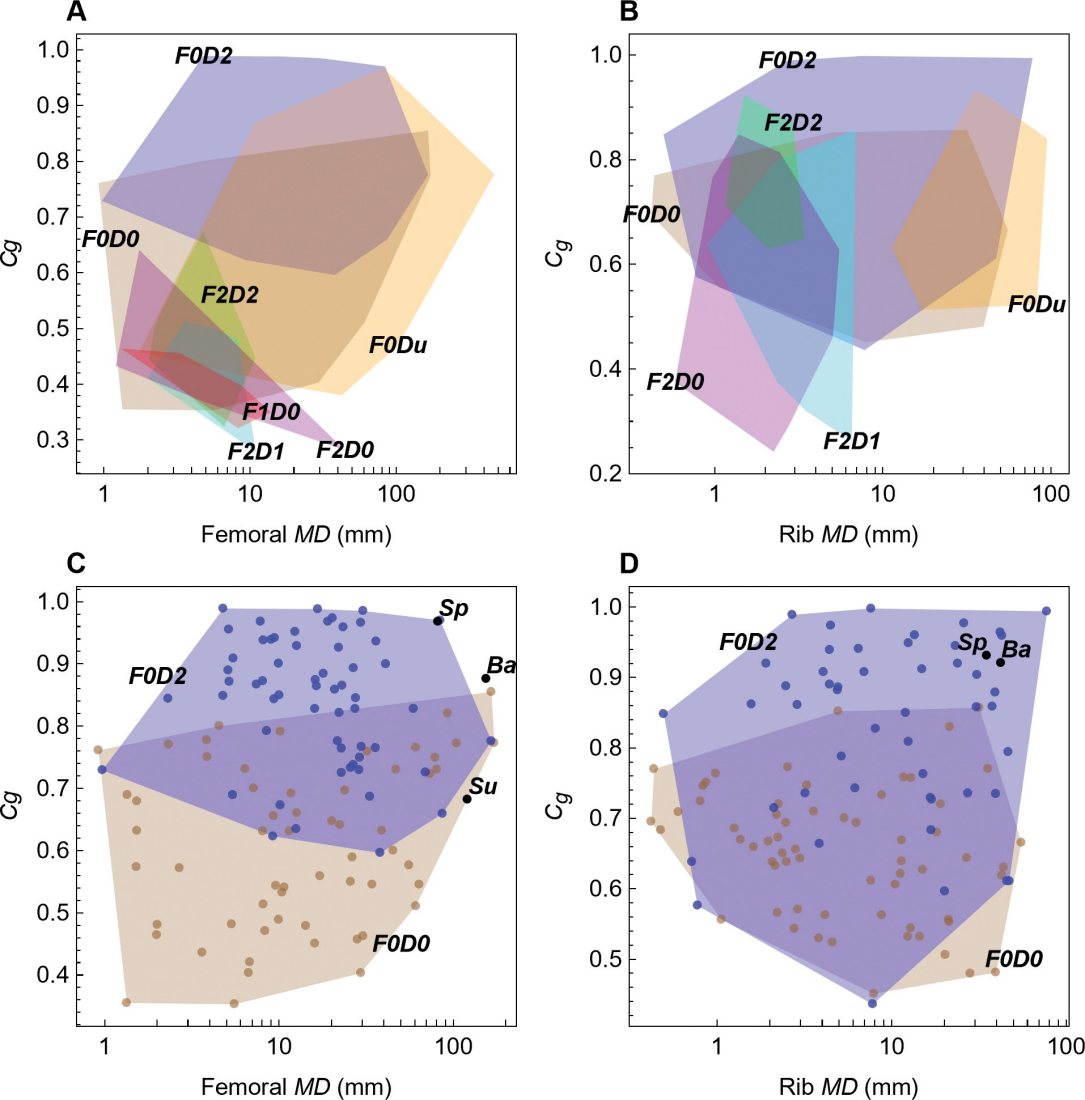
Lifestyle overlap in femoral and rib data from Fabbri *et al*. [15]. Femoral (A, C) and rib (B, D) plots of maximum bone diameter (*MD*) versus bone compactness (*Cg*). (A, B) Convex hull polygons colored by functional group, as defined by Fabbri *et al*. Groups with four or fewer datapoints not shown. (C, D) Points and corresponding convex hull polygons for terrestrial groups (*F0D0*) and groups that include nonflying divers (*F0D2*). Abbreviations: 0, absent; 1, rarer; 2, habitual; Ba, *Baryonyx*; D, diving; F, flying; Sp, *Spinosaurus*; Su, *Suchomimus*; u, unknown.

Fabbri *et al*. compiled datasets of (*MD*, *Cg*) points from femoral and rib cross sections representing exemplar taxa from many disparate clades of reptile, mammals, and birds. They manually coded each taxon with two lifestyle attributes, *F* (for flying ability) and *D* (for diving), using a three-value scale: 0, absent; 1, rarer; 2, habitual (see Materials and methods for details). The attribute combinations were then used to divide the taxa into functional groups, for example *F0D0* taxa are terrestrial, whereas *F0D2* taxa are nonflying divers. In Fig 1, convex hull polygons plot the extent of the datapoints for each group.

The femoral data for all classes shows extensive overlap among the polygons defined by the (*MD*, *Cg*) points (Fig 1A). The flying classes (*F1D0*, *F2D0*, *F2D1*, *F2D2*) all overlap each other, and most also overlap the *F0D0* class of terrestrial animals that cannot fly and seldom if ever dive. The rib data, though visually different, exhibit no less overlap (Fig 1B).

To classify the spinosaurid dinosaurs, the most relevant comparison is group *F0D0* to *F0D2*, *F0D2* being the nonflying taxa that are habitual “subaqueous foragers,” in the terminology of Fabbri *et al*. [15]. These are shown in Fig 1C and 1D. The pFDA statistical method employed in their study seeks a straight line, called the decision boundary, that cleanly separates datapoints by class. In this case, that means finding a straight line that has the blue points on one side and the brown points on the other side in Fig 1C and 1D. As these plots clearly demonstrate, this is not possible—the blue and brown points are too intermingled. Any line that one did draw would misclassify many of the known taxa by putting them on the wrong side of the line. A method that cannot accurately classify known taxa is suspect when applied to classifying unknown taxa such as the spinosaurids.

The implicit assumption made by Fabbri *et al*. is that a complex statistical method (pFDA) can somehow draw that decision boundary to make an accurate classification. While it is true that in some cases statistical methods can achieve surprising results, we show below that is not the case here. Instead, the complex and opaque statistical methods used by Fabbri *et al*. obscured a fundamental difficulty with classification that arises from the use of these training datasets.

Moreover, statistical analysis is built upon a chain of steps. Performed properly, such analysis can allow surprising conclusions to be drawn with great scientific rigor. However, we find critical problems with many of the steps Fabbri *et al*. conducted in their analysis. Any one of these flaws is sufficient to greatly diminish the probative value of their conclusions; some are sufficient to refute those conclusions altogether. Our study examines each of the problems in detail to elucidate the issues that future research must address when using pFDA, bone compactness metrics, and related methods.

We conclude that the number and nature of the problems with the results reported by Fabbri *et al*. render the approach used in that study largely invalid and of little evidentiary value. The most generous interpretation of those results is that *Spinosaurus* and *Baryonyx* (but not *Suchomimus*) have a slight statistical affinity with animals that have a range of semiaquatic adaptations. A result of this kind would not be helpful in choosing among the conflicting hypotheses for spinosaurid ecology.

As we demonstrate in this study, the pFDA method must be used with some caution because it neither includes tests of its distributional assumptions on the datasets nor natively provides estimates of uncertainty in its classifications that arise from the sample size or other properties of the dataset. Here, we supplement pFDA with explicit tests of the distribution assumptions and find that the dataset used by Fabbri *et al*. fails to meet those tests. Indeed, some portions of the dataset are statistically indistinguishable from uniform random distributions of points.

## Materials and methods

### Institutional Abbreviations

BSPG Bayerische Staatssammlung für Paläontologie und Geologie, Munich, Germany.

FSAC Faculté des Sciences Aïn Chock, University of Casablanca, Morocco.

MNBH Musée National Boubou Hama, Niamey, République de Niger.

CMN Canadian Museum of Nature, Ottawa, Canada.

UCRC University of Chicago Research Collection, Chicago, United States of America.

### Datasets and methods of Fabbri *et al*

The materials and methods we used in our study are best understood in the context of those employed by Fabbri *et al*., so we first summarize the relevant datasets and methods reported in their paper and a subsequent preprint [15,16].

The pFDA method, described below, uses a training dataset comprising several exemplar datasets, each of which is divided into subsets known as classes. Data points in each class share a class property, such as value or range of values of one or more categorical variables. The algorithm analyses the training data, along with test data points. As implemented by Fabbri *et al*., each datapoint corresponds to a specimen of a particular taxon, and two categorical variables are used to assign taxa to classes.

Fabbri *et al*. represented each specimen in a sampled taxon with a two-dimensional data point (log_10_(*MD*), *Cg*). The parameter *MD* is the maximum diameter of the sampled bone (either femur or rib). The parameter *Cg* can be calculated from an image of a bone cross section binarized based on presence or absence of bone. This image can be derived either from a thin-section photomicrograph or a CT radiograph, as detailed below. Fabbri *et al*. gathered the majority of *Cg* and *MD* measurements in their datasets from the literature; when they collected their own measurements, they used the BoneProfileR program [17,18] to calculate *Cg*.

The two primary datasets employed by Fabbri *et al*. are based on femoral cross sections from one set of taxa and dorsal rib cross sections from a different but largely overlapping set of taxa. Most taxa are represented by a single data point in the femoral dataset, the rib dataset, or both. Multiple datapoints were included for some taxa. Fabbri *et al*. constructed a phylogenetic consensus tree across the taxa. The tree is used in their statistical methods to correct for phylogenetic bias in either phylogenetic general least squares (PGLS) regression or pFDA analysis, using standard methods.

In the initial phase of their analysis, Fabbri *et al*. assigned two categorical variables to each taxon data point. For clarity and concision, we abbreviate the functional groups identified by Fabbri *et al*., using *F* and *D* to designate “flying” and “diving,” respectively, for the two lifestyle behaviors they identified, along with the values (0–2): “absent” (0), “present but infrequent” (1), and “frequent” (2). Here we abbreviate each taxon group as *FxDy*, where *x* and *y* denote the values of the flying and diving variables, respectively.

Habitual (frequent) divers—“subaqueous foragers” in the terminology of Fabbri *et al*.—include taxa such as the emperor penguin *Aptenodytes*, which was assigned categorical variables *F0D2*. As another example, the razorbill *Alca torda* is an extant seabird that frequently flies and dives, so it was classified as *F2D2*.

In summary, the datasets include two skeletal elements (femur and rib), two measured variables (*Cg* and *MD*), and two categorical variables (flying and diving), each of which can be assigned any of three values. Fabbri *et al*. make no distinction in their coding of variables between extinct and extant status, but that distinction is important in some of our analyses. Table 1 summarizes the some of the important subgroups.

**Table 1:** Example functional groups and subgroups in Fabbri *et al*. [15], as designated in this paper.

Our abbreviation	Lifestyle	Taxon type	Subgroups
***F0D0***	Terrestrial (nonflying/nondiving)	All	Terrestrial
		Extant	Extant terrestrial
		Extinct	Extinct terrestrial
***F0D2***	Nonflying diver (nonflying/frequent diving)	All	Divers
		Extant	Extant divers
		Extinct	Extinct divers
***F2D2***	Flying diver (frequent flying & diving)	All	Flying divers
		Extant	Extant flying divers
		Extinct	Extinct flying divers

Fabbri *et al*. [15] assigned for “flying” the values 0 = “unable” to fly, 1 = “nonsustained flight,” and 2 = “sustained flight.” They assigned for “diving” the variables 0 = “unable” to dive, 1 = “infrequent” diving, and 2 = “frequent” diving. Some taxa were designated “unknown” rather than assigned a variable. The three most important subgroups are shown in the table.

Fabbri *et al*. listed the taxon names, which are stated to be shared between the rib and femoral datasets, in their Supplementary Table 1 [15]. Tables of taxon data in a spreadsheet file, along with an R script computer code, were published in their Supplementary Dataset [19]. However, the code does not read the published spreadsheet and instead reads a set of four different spreadsheet data files (Table 2). These previously unpublished files, provided to us by Fabbri *et al*. via email, are in the S1–S4 Files accompanying this article.

**Table 2:** Four training datasets used in Fabbri *et al*. [15].

Dataset label	Bone	Taxa included	Source file
**ds1**	Femur	200	Femur_compactness_all.csv
**ds2**	Rib	174	Rib_compactness_all.csv
**ds3**	Femur	187	Femur_compactness_no_graviportals_no_pelagics.csv
**ds4**	Rib	148	Rib_compactness_no_graviportals_no_pelagics.csv

Training datasets read by the R scripts that generate the pFDA and PGLS results of Fabbri *et al*. (obtained by personal communication). Taxa included is the count in the dataset across all categorical variables.

Comparison of the data in the published spreadsheets with the files from Table 2, the Supplementary Table 1 of ref. [15], and the text of that paper reveals unexplained discrepancies. Two taxa present in the files from Table 2 (one each in the femur and rib files) do not appear in the associated phylogenetic tree. As a result, these two taxa are automatically discarded by the tree-matching routine in the pFDA code used by Fabbri *et al*. Whereas the body of the paper states that 83 taxa were shared between femur and rib datasets, we count 76 shared taxa. We were nevertheless able to replicate the published results of Fabbri *et al*. using the code and the previously unpublished data files (Table 2), which indicates that they contain the data used to generate the results of the paper.

Datasets ds1 and ds2 (Table 2) include all femoral and rib taxa, respectively. Dataset ds3 is a subset of ds1, and ds4 a subset of ds2, in which selected taxa were removed, as detailed below. Supplementary Table 6 of [15] lists all of the taxa removed from ds2 to form ds4. But Supplementary Table 5, the corresponding table for ds1 and ds3, omits without explanation the taxa *Choeropsis liberiensis* and *Desmostylus hesperus*, which are in ds1 but missing from ds3.

In their main text and Supplementary Tables 5 and 6, Fabbri *et al*. labeled the taxa to be removed as “deep diving.” Elsewhere in their Supplementary information, as well as in the file names, they instead used the term “pelagic.” These terms are not interchangeable, as they convey very distinctive—and sometimes nonoverlapping—lifestyles.

#### Analytical stages of Fabbri *et al*

Fabbri *et al*. employed a three-stage analytical method. The first stage performed PGLS to regress *Cg* (as the dependent variable) against the categorical lifestyle variable *D* (results in Table 1 of ref. [15]), and then regressed *Cg* (as dependent variable) against all combinations of the categorical lifestyle variables and *MD*. ANOVA results were presented [15: Supplementary Tables 3 and 4]. The results show very weak but statistically significant correlations in some cases, with *P* values reported as low as 0 (presumably due to rounding) but *R*^2^ = 0.176 for femoral data ds1, and *R*^2^ = 0.108 for rib data ds2.

In the second stage of their analysis, datasets were prepared for pFDA. Datasets for each skeletal element (*i*.*e*., femur or rib) were sorted by the categorical variables to yield two classes: nonflying subaqueous foragers (*F0D2* using the abbreviations of this study) and everything else (*F0D0*, *F0D1*, *F1D0*, *F1D1*, *F1D2*, *F2D2*); the class assignments are listed in the spreadsheet files of Table 2. These two classes were subsequently used for the classification of test taxa. Fabbri *et al*. stated that “our inference has only two possible outcomes: subaqueous forager or non-subaqueous forager” [15].

The third and final stage of their analysis applied the pFDA algorithm to process the training datasets and then classify other data points representing test taxa, including spinosaurids. The algorithm is coded in an R script that builds on base-level pFDA code deposited by Motani and Schmitz in an online repository [20].

pFDA requires a phylogenetic tree across all taxa as input. The original pFDA papers [21,22], and the available code repository [20] used training datasets of entirely extant taxa. The same is true for all prior uses of pFDA that we could find via searches on Google Scholar for citations of the original papers or repository (representative examples include [23–26]). Fabbri *et al*. instead used training sets that mix extant and extinct taxa. To account for uncertainty in the timing of the phylogenetic tree nodes for extinct taxa, their method adopted an *ad hoc* approach, which is not referenced as occurring elsewhere: “We repeated analyses across 100 informal supertrees with varying branch lengths to account for stratigraphic uncertainty” [15]. This was done for both their PGLS and pFDA analyses. Their R code creates the random trees and loops over them.

This stage resulted in a set of assignments classifying the test taxa into the two classes in the training set. For each assignment, it generated a posterior probability of class membership in the *D =* 2 class, denoted *P*_2_ hereafter. Because each run of the pFDA produced 100 results—one result for each of the 100 random phylogenetic trees—Fabbri *et al*. presented the median of the set of *P*_2_ values. The default classification criterion was to assign a test taxon to a class if the posterior probability was ≥0.5; the classifications across the 100 trials were reported as a count of the number the trials classified as belonging to the *D =* 2 class.

### Methodology for measurement of bone compactness

We performed new *Cg* measurements on specimens not included in the datasets of Fabbri *et al*., and we also attempted to replicate some of their measurements of *Cg*. We used Materialise Mimics Innovation Suite 23.0 to segment computed-tomographic (CT) scans of specimens new to this study. We positioned long bones for cross section perpendicular to the shaft axis. We used a threshold that highlighted bone and exported that highlighted image of the cross-sectional slice.

We used the BoneProfileR R package [17,18] and the binarized femoral slice images provided by Fabbri *et al*. in their Fig 1 and S1–S5 Figs [15] to measure *Cg*. To ensure that pixels were correctly read by BoneProfilerR, Affinity Photo was used to binarize all new images. Because user-input parameters for the BoneProfilerR program were not reported in Fabbri *et al*., some variance in our results is expected. For complete sections, we used the ontogenetic center (recommended by the authors of BoneProfileR [17]) in the BP_EstimateCompactness function and defaults of 60 angles and 100 distances. We collected bone compactness data from the flexit and flexit-with-pi rotation models. There were three partial cross sections, which were run using a user-defined center with setting partial = TRUE in the BP_EstimateCompactness function. A few of the cross sections published by Fabbri *et al*. are of low resolution, necessitating rebinarization.

### Computed tomography

In order to provide measurements of *Cg* for spinosaurids on specimens not considered in Fabbri *et al*., CT scans were acquired for femora of *Suchomimus tenerensis* (MNBH GAD500, MNBH GAD72) and *Spinosaurus aegyptiacus* (FSAC-KK 11888) at the University of Chicago Hospitals by Dr. Nicholas Gruszauskas and Dr. David Klein using a Philips Brilliance iCT 256-slice multi-detector CT scanner. CT scans for the additional *Spinosaurus sp*. femora (CMN 41869, CMN 50382) were generated by Vincent Bolduc at the Transportation Safety Board of Canada’s North Star Imaging CT scanner. Scan settings for each of the specimens are included in S1 Table.

### Statistical method implementations

All pFDA results in this paper were based on R scripts and associated data files obtained from the authors of Fabbri *et al*. [15] and on base-level pFDA code deposited by Motani and Schmitz in an online repository [20]. Bootstrap trials and related modifications were implemented in R, with minimal changes necessary to the base-level pFDA code for debugging.

Bootstrapping pFDA consists of randomly selecting with replacement a sample of the dataset taxa of the same length as the original dataset, and then running the analysis on each such trial set. The selection process results in bootstrap samples that may omit some specimens from the original dataset and may include other specimens more than once. As is typical in bootstrap analysis, 2000 trials were done for each bootstrap run [27–29]. Consistent with the approach of Fabbri *et al*., we created 100 random phylogenetic trees for each such trial, so a single bootstrap analysis of a dataset created 200,000 individual pFDA runs.

Phylogenetic trees must be pruned appropriately, which was accomplished in the same manner as pFDA, using the same R library functions that were employed by the pFDA code from Motani and Schmitz [20] that was used by Fabbri *et al*. As a verification step, the phylogenetic matrices and transformed datasets were independently calculated with Phylogenetics-for-Mathematica [30]; we found identical results within expected numerical precision. Output data from the pFDA functions, including the confusion matrix and posterior probabilities, were saved in files for later analysis and plotting. Confidence intervals on bootstrap output data were computed using the bias-corrected and accelerated (BCa) bootstrap algorithm [27–29], which is based on both bootstrap and jackknife trials. This was implemented by the authors in Mathematica. The R code, Mathematica bootstrap code, and other Mathematica code used in this study are available in an online repository [31].

In our checks for possible selection bias in the taxa included in the datasets, we performed permutation tests on the rank distribution of *Cg* between extinct and extant taxa, using Mathematica to implement standard methods [32]. Statistical analysis of the output of the trials gathered in R, along with the data tables and figures, were generated with code written in Mathematica 13.2 [33]. Statistical tests, such as Brown-Forsythe, Conover, and Levene variance equivalence tests, used standard library functions in Mathematica. Other library functions were used to fit distributions in the construction of smooth kernel distribution plots and quantile-quantile plots.

Code was written by the authors for simple LDA (linear discriminant analysis) and a Monte Carlo simulation using LDA, which are described in the next subsection. Eq (3) in the section below on the pFDA method was derived in Mathematica.

Statistical distributions were fit to data using standard library functions in Mathematica. Code written by the authors in Mathematica calculated AIC and AIC_c_ values and Akaike weights for distribution fits using standard methods [34]. Mathematica was also used to generate all the graphs and plots in the paper.

To test whether data points generated by pFDA exhibit genuine clustering, we used the Hopkins statistic. Code implementing Hopkins statistic tests was written by the authors in Mathematica, using the published algorithms [35,36]. Under the null hypothesis, the Hopkins statistic is expected to approximate a beta distribution: Beta (*m*, *m*), where *m* is the number of points sampled. As recommended in the literature [35,36], a random sample of 20% of the points in a test set was used, and the *H* statistic was calculated as the mean of 100 random trials. As an additional verification, a Monte Carlo suite of 10,000 pseudorandom examples of a uniformly random distribution were generated and tested to build an empirical sampling distribution for the null hypothesis. This was done separately for each of the variants of the Hopkins statistic test, as well as for each point count in a set being tested.

#### The pFDA method

Fabbri *et al*. used pFDA to reach their conclusions regarding the identification of habitual behaviors in extinct tetrapods. pFDA, a phylogenetic adaptation of flexible discriminant analysis (FDA), was first applied to study nocturnality in dinosaurs via statistical analysis of eye and scleral ring shape [21,22]. FDA, in turn, is a generalization by Hastie *et al*. [37] of Fisher’s much earlier linear discriminant analysis (LDA) [38].

Fisher created LDA to separate classes of data. Each class is represented by a set of points (in dimensions 2 or greater) drawn from multivariate normal distributions. The distribution for each class must have a distinct mean (centroid), but all classes must share the same covariance matrix. Later work has shown that LDA is closely related to ANOVA and regression techniques [39]. LDA computes the coordinates of a line, called the decision boundary, that divides the points into regions that can be classified into the nearest class. LDA has previously been used with bone-compactness data to discriminate among (classify) groups without incorporating phylogenetic data in the analysis [40–44].

The general form of the probability density function for a bivariate normal distribution is given by Eq (1), where *x* is a two-dimensional position vector, *μ* is a two-dimensional position of the centroid of the distribution, Σ is a 2×2 covariance matrix that has |Σ| as its determinant, and the superscript ^T^ denotes matrix transpose. The probability function for class *k* is given by

$$P_{k}(x,\mu_{k},\Sigma )=\frac{e^{-\frac{1}{2}{\left(x-\mu_{k}\right)}^{T}\Sigma^{-1}(x-\mu_{k})}}{2\pi \sqrt{\left|\Sigma \right|}}.$$ (1)

In the case of two-class or binary classification, LDA assumes that there is a different distribution for each class, with centroids *μ* = *μ*_*1*_, *μ*_*2*_ for classes *k* = 1,2. The centroids must be distinct (*i*.*e*., *μ*_*1*_≠*μ*_*2*_*)*, but both distributions have the same covariance matrix Σ. Mathematically, this assumption ensures that the decision boundary is a line [39].

A related classification method that allows each set to have a different covariance matrix is known as quadratic discriminant analysis (QDA) because the decision boundary between the datasets is a quadratic curve (*i*.*e*., a conic section). If LDA were applied to such a dataset, however, one would expect highly inaccurate classification because the straight-line assumption is violated [39].

LDA classifies points by computing the Mahalanobis distance from a test point to the centroid of two or more reference groups, using the pooled, within-group covariance matrix [39]. The squared Mahalanobis distance appears in an argument to the exponential function in Eq (1). In the case of a distribution with unit variance and a covariance matrix that is the identity matrix, *i*.*e*., $\Sigma =\left(\begin{array}{cc} 1 & 0\\ 0 & 1 \end{array}\right)$, it reduces to the Euclidean distance.

In LDA and FDA, a fundamental assumption is that a test point can be classified by assigning it to the group that has the smallest Mahalanobis distance between the point and the group centroids μ_1_, μ_2_ (*i*.*e*., the multidimensional means of the classes). The locus of points equidistant between group centroids corresponds to the decision boundary; for LDA and pFDA, that is a line. Hastie *et al*. generalized LDA to FDA by using a general framework that allowed general nonlinear decision boundaries. They also added support for a Bayesian approach, using prior probabilities [37].

All of these methods (LDA, FDA, pFDA) perform a geometric transformation to find the directions in which the variance between the sets is minimized and maximized. This acts as dimensional reduction; in a system with two classes and two-dimensional data points, the geometric transformation projects the data into one-dimensional points called discriminants that are used perform classification and assign posterior probabilities of class membership [39].

Motani and Schmitz [21] introduced pFDA as a specific instance of FDA in which a phylogenetic-bias correction is performed in a similar fashion to PGLS, by using branch lengths from phylogenetic trees that cover the taxa in the analysis to determine phylogenetic correlations among taxa under an evolutionary model, such as Brownian motion. In principle, FDA could allow the use of nonlinear decision boundaries, but pFDA as implemented by Motani and Schmitz [20] (and used by Fabbri *et al*.) is restricted to using linear boundaries, thereby assuming that both groups have the same covariance matrix, as in Eq (1). pFDA is thus a phylogenetic version of LDA. As currently conceived, pFDA does not allow classes to have different covariance matrices as with QDA, nor does it allow other classes of curves or relation of distributional assumptions. Conceivably a pQDA or a quadratic variant of pFDA could be developed, but this has not been proposed, nor is it used by Fabbri *et al*.

The procedure presented in Motani and Schmitz [21] uses extant taxa that have well-constrained phylogenies and branch lengths for the training set. The use of data from extant taxa in the training set has several advantages. One is that the phylogenies are likely to be better known, reducing the possibility that error from the phylogeny could confound the results. However, the primary reason is that Motani and Schmitz were seeking to classify a behavioral pattern (*e*.*g*., whether daily activity was primarily nocturnal or diurnal), which can be observed in living organisms but is not directly accessible for extinct taxa.

Using extant taxa to make a classification inference on extinct taxa implicitly presupposes that the statistical distribution of the variables used in the analysis is the same for extinct and extant taxa. Otherwise, one could come up with a criterion boundary based on extant taxa that would have unknown relevance to the extinct test taxon.

In the case of the Motani and Schmitz study, the variables were eye-related dimensions, which have a strong theoretical basis in optical physics, so consistency in distribution across millions of years of evolution is highly plausible. As a result, it was not a major concern for their study. However, such temporal invariance in distribution is not automatically guaranteed when pFDA is applied to other variables.

In an extensive literature search, we were unable to find any other study that trained the pFDA classifier on mixed extinct and extant taxa, as Fabbri *et al*. did. To address the potentially greater phylogenetic uncertainty with extinct taxa, Fabbri *et al*. created 100 trees of random branch length, each having its own associated phylogenetic covariance matrix. The matrices are sequentially passed to code that performs FDA, resulting in 100 classification probabilities for each test taxon—one for each random tree. Any new method such as this should be accompanied by evidence that it performs as intended to address the problem of uncertain phylogeny and that the parameters chosen—*e*.*g*., the number of trees, the random assignment of branch lengths, ignoring different tree topologies—are sufficient for the classification task. Fabbri *et al*. presented no such evidence or justification.

Fabbri *et al*. used a low threshold of 50% on the median probability, which results in weak classifications. The use of the median is not justified because a median discards random trees that, by construction, are all equally likely to represent past evolution. A better approach, which is widely used in the statistical literature, is to estimate a confidence interval, such as the 95% confidence interval.

Although in principle the phylogenetic signal could have a strong effect, in practice, Fabbri *et al*. find very little evidence of phylogenetic signal in their dataset, with Pagel’s λ parameter taking values 0.02≤λ≤0.07 across the various datasets and trials. This is consistent with other studies of *Cg* that used comparable datasets to analyze convergent features across many clades [45,46]. As a result, one would expect little difference between these results and those obtained with ordinary LDA. In view of that and the uncertainty in the tree for extinct taxa, we question whether the use of a phylogenetic method is worth the added complexity for this dataset.

To illustrate the properties of LDA and pFDA, we consider a special case of Eq (1) for two distributions having the properties given in Eq (2), where the covariance matrix Σ is identical for both distributions and is a multiple *σ*^2^/2 of the 2×2 identity matrix.

$$\Sigma =\sigma^{2}\left(\begin{array}{cc} 1 & 0\\ 0 & 1 \end{array}\right),\mu_{1}=-\mu_{2}=\left(\begin{array}{c} 1.2\\ 1.2 \end{array}\right)$$ (2)

**Fig 2:**
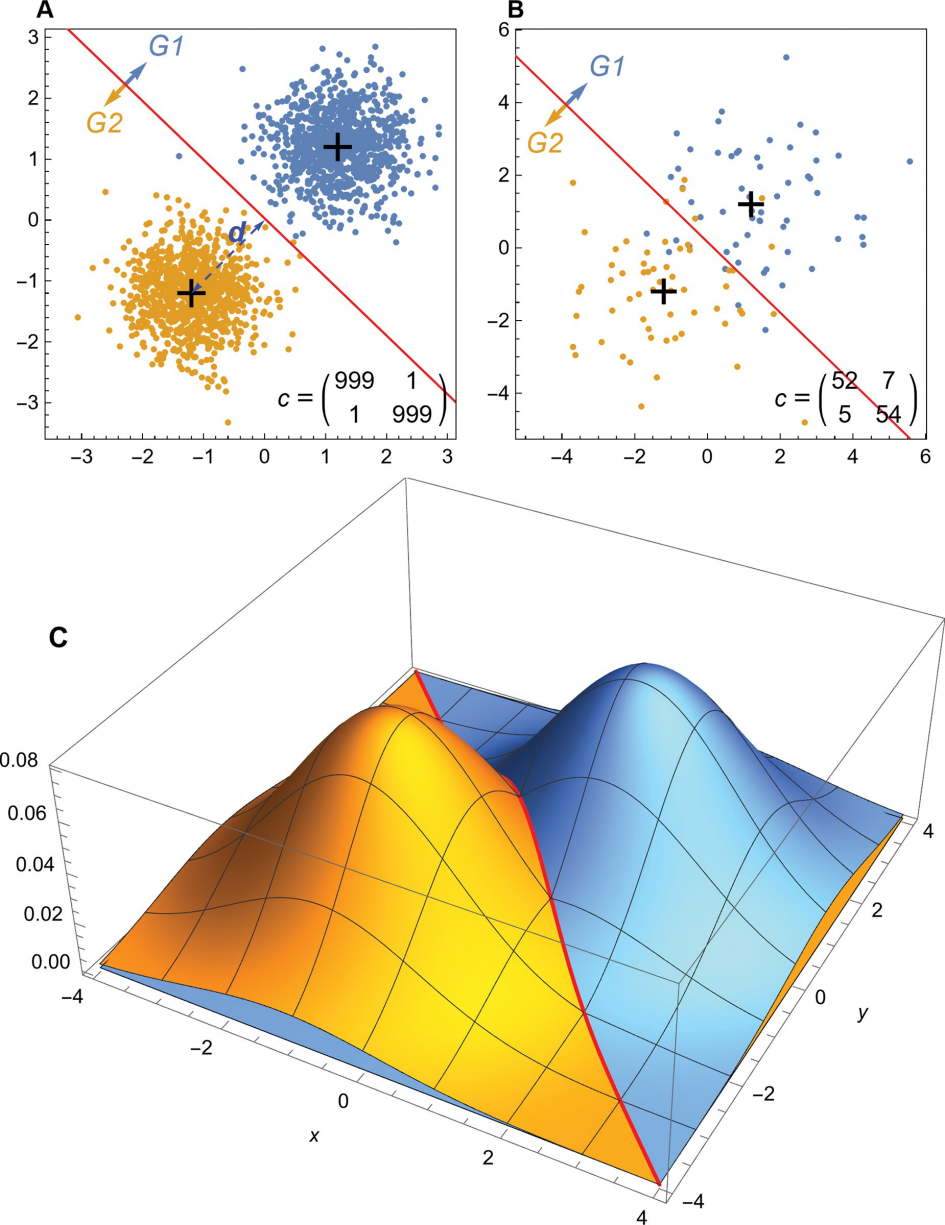
Simulated data plots for LDA methods. (A) 1000 pseudorandom points drawn from each of two multivariate normal distributions given by Eqs (1) and (2) and *σ* = 0.55, with points from each distribution colored according to the legend. The decision boundary for LDA is given by the red line; points above the line are classified as group 1, points below the line are classified as group 2. Note that one point from group 1 lies on the other side of the decision boundary and is incorrectly classified as group 2. One point from group 2 is similarly misclassified. The centroid of each distribution is denoted by a black cross, the distance *d* from the centroid to the decision boundary is denoted by a dashed blue line. The confusion matrix *c* (Eq (5) in S1 Appendix) is shown. (B) 59 points from distributions with the same centroids as (A) but with *σ* = 1.414. The higher value of *σ* leads to a larger number of points being misclassified. (C) The underlying probability density functions for the same distributions as in (B). The distributions of blue and gold points are equal at the red decision boundary line *y* = −*x*. Abbreviations: G1, group 1; G2, group 2.

The centroids of the two distributions, *μ*_1_ and *μ*_2_, are reflected in the origin across the line *y* = −*x*. It is easily shown that the decision boundary must be the perpendicular bisector of the line between the centroids *μ*_1_ and *μ*_2_. In this case, *μ*_1_ and *μ*_2_ both lie on the line *y* = −*x*, and thus *y* = *x* is the bisector. Fig 2 plots 1000 points drawn from each of two distributions, denoted group 1 and group 2. In both cases, *σ* = 0.55 and plays a role in these bivariate distributions that is very similar to the parameter in a conventional univariate normal distribution. The distance between the centroid of either distribution and the decision boundary is *d* = 1.7 = 3.1*σ*. As a result, the concentration of points matches what one would expect of a univariate normal distribution: most of the points are concentrated near the centroid and thus appear on the same side of the decision boundary as the centroid. Such points would be correctly classified by the decision boundary.

Points that fall on the opposite side of the decision boundary are considered *misclassified*. Because these points are part of the training dataset, they are termed training data errors [39]. Because the points are highly concentrated and the decision boundary is relatively far in terms of *σ*, there are only a few of these points in the random sample shown. Fig 2B shows an example with the same distribution centroids, but with 59 points in each group and *σ* = 1.414, such that *d* = 1.2*σ*. The shorter distance in terms of *σ* greatly increases the number of training data errors.

The fundamental idea behind LDA is shown in a plot of the probability density functions for the multivariate normal distributions given by Eqs (1) and (2) (Fig 2C) with the same *σ* = 1.414 used to generate Fig 2B. The two normal distributions intersect at a 3-D curve that falls along the line *y* = −*x* when projected onto the (*x*, *y*) plane. That decision boundary is where the probabilities of membership in both probability density functions are equal. Away from that boundary, one probability is greater than the other.

One can calculate the exact probability that a point will lie on the wrong side of the decision boundary by integrating the probability density function over the half plane defined by the wrong side of the decision boundary to yield Eq (3), where erfc() is the error function and *d* is the distance from the distribution centroid to the decision boundary.

$$P_{wrong}=\frac{1}{2}erfc\left(\frac{d}{\sigma \sqrt{2}}\right)$$ (3)

This relation matches the familiar case of the marginal distribution of points in a normal distribution, expressed in terms of the standard-deviation-adjusted distance (*i*.*e*., the ratio *d*/*σ*). Thus, we expect from Eq (3) that 68.27% of the points would be misclassified if *d* = *σ*, 2.5% of the points to be misclassified if *d* = 1.96*σ*, and 1% if *d* = 2.33*σ*, following usual rules of thumb.

In the example shown in Fig 2B, *P*_wrong_ = 0.115, so we expect that about 11.5% of points in each group will be misclassified if the number of points is very large. For the distributions in Fig 2A, *P*_wrong_ = 0.00955, or roughly 1 in 1000. The one classification error seen in group 1 and one error seen in group 2 thus match expectations. As the number of trials increases, the number of incorrect points converges toward *n*×*P*_wrong_, with some statistical variation.

Eq (3) reveals an important principle: even when we use simulated data drawn from multivariate normal distributions, classification via LDA or FDA can *never be error-free*. That follows from the simple fact that the domain of the multivariate normal distribution ranges across the interval (−∞, ∞) in each independent variable, whereas the distance from the distribution centroids to the decision boundary is finite. Therefore, there can always be valid points from one distribution that lie on the other side of any decision boundary—not as an outlier (which implies an erroneous point) but rather as an entirely valid data point that LDA will misclassify. Note that this effect does *not* depend on the sample size. As the number of data points in the training set grows to infinity, the error converges to Eq (3).

#### Assigning confidence to classifications

Sound statistical practice recognizes that random variations do occur and can lead to false inferences, even when statistical methods are applied correctly. Inferences are thus routinely qualified and evaluated. Results should be qualified by providing quantitative estimates of their statistical quality, such as the *P* value, confidence level, confidence interval, or other measures. Those quality estimates should then be evaluated against widely accepted thresholds for statistical significance, such as the current de facto standard of 95% significance, often expressed as 5% random error, *p≤*0.05, or a 95% confidence interval (CI). Although studies do sometimes employ other standards with justification (ref. [47] and S1 Appendix, section 3), Fabbri *et al*. selected the conventional 95% significance threshold for their PGLS and ANOVA analyses [15: Table 1, Supplementary Tables 3 and 4].

Because LDA and FDA were not designed for hypothesis testing or statistically rigorous inference, they do not natively produce a formal *P* value, confidence interval, or other metric of random effects. These methods are typically used for *ad hoc* applications of statistical learning or machine learning, often on ill-posed problems such as handwriting recognition [37].

The pFDA method inherits this weakness from its predecessor methods. As a consequence, classification by running the pFDA algorithm does not by itself offer a rigorous statistical test. Strictly applied, statistical standards would rule out the use of pFDA as the basis for scientific conclusions until a rigorous theoretical framework has been developed that can assess the quality of pFDA classifications.

In the absence of such a framework, we attempt here to estimate the statistical quality of pFDA with two available tools: posterior probabilities and empirical classification performance on known cases. An invocation of a pFDA classifier returns a list of the predicted probabilities of class membership for each of the test taxa to be classified. We denote as *P*_2_ the pFDA estimate of posterior probability that a point in the datasets of Fabbri *et al*. belongs to the class *D = 2*. Because each test point is classified for 100 random phylogenetic trees, the result for a single taxon is typically a list of *P*_2_ values of length 100. If the median value of the *P*_2_ list is greater than 0.5, Fabbri *et al*. classified the taxon as *D =* 2.

Fabbri *et al*. acknowledged that a 50% probability is an unusually weak criterion for assigning class membership [15: 859]:

We summarised our results by providing the median value of those 100 posterior probabilities and the number of times a particular taxon is predicted as subaqueous forager (median probability of 50% or more). This gives us two proxies of the likelihood of each taxon to be an actual subaqueous forager. For instance, a taxon could be predicted 100 times as subaqueous forager with a median probability of 51% which means the evidence for this extinct species to be an actual subaqueous forager is very weak and this inference has to be considered very unlikely. Median probabilities need to be within the range of 80–100% to be considered as strong evidence of subaqueous forager.

Because there are two classes, a classification probability of 0.5 is the accuracy we would expect from a random guess, such as flipping a coin. Normally, a result that is only infinitesimally better than random would be accorded little probative value. Nevertheless, this weak criterion was used for classification rather than the stronger values of *P*_2_>0.8 or *P*_2_ = 1.0 that are suggested in the passage.

If *P*_2_ were an absolute probability, then *P*_2_ = 1.0 would indicate no possibility of misclassification. But *P*_2_ is *not* an absolute probability—instead it is a classification score that, at best, provides a possibly erroneous estimate of the *relative* probability of being in one class versus the alternative, conditioned on the prerequisite that the classes are multivariate normal distributions [37]. Furthermore, as used by Fabbri *et al*., *P*_2_ is not a single value but rather a list of 100 values from their Monte Carlo trials; it is fundamentally a statistical quantity. In this study, we build on this treatment of *P*_2_ as a statistical quantity by also including bootstrap trials, which explore the error due to finite sample size—*i*.*e*., statistical variation arising from the finite size of the training dataset.

Each *P*_2_ value is derived from the ratio of the probabilities given by the normal distribution describing each class, distributions that should have different centroids but same variance. Test points are often distant from the centroids and thus often fall in the tail of the distribution for one or both of the classes. Tail probability estimates derived from a finite sample of data points can be uncertain, particularly in the case that the points are near or outside the edge of the points in the training dataset. Thus, the computation of *P*_2_ is extremely sensitive to the conformance of the datasets to the stated assumptions of being normal, having different means, and having the same covariance matrix.

Reporting a median value of a Monte Carlo experiment without a confidence interval, as Fabbri *et al*. did, is entirely out of keeping with conventional statistical practice. We report 95% confidence intervals, as is standard in many scientific disciplines.

Due to the statistical uncertainty in the value of *P*_2_, the classification threshold should not be that median *P*_2_≥0.95—the threshold used by Fabbri *et al*.—but rather that the lower bound of the 95% CI on *P*_2_ must be greater than or equal to 0.95. This heuristic effectively requires 95% confidence that the classification is at least 95% correct. The threshold value for this heuristic is “within the range of 80–100% to be considered as strong evidence” that Fabbri *et al*. propose, but it is implemented using the standard technique of the 95% confidence interval rather than the median.

In contrast, a correct interpretation of the criterion that the median *P*_2_≥0.95 is that 50% of the time we should expect that there is more than 5% classification uncertainty. That weaker criterion is not possible to reconcile with conventional standards for statistical significance or confidence. Although one could argue for demanding 100% confidence that the classification is 95% correct, we did not use that approach because we felt that adherence to the commonly used 95% confidence interval is important.

To be clear, this is the criterion for strong evidence, not the baseline classification criterion. It may seem that a higher *P*_2_ classification threshold for all classification (not just the strongest) would be a better choice, but the situation is more nuanced. Increasing the classification threshold does make for a more stringent criterion, but it also results in misclassification of a greater percentage of the training dataset (S1 Appendix, section 3).

*P*_2_ indicates the strength of the prediction for a particular taxon; the values and confidence intervals for *P*_2_ will vary from taxon to taxon. To assess pFDA classification performance overall, it is useful to evaluate how well the classification performs on known cases by assessing training data errors (misclassifications of the training set), a standard technique in the statistical and machine-learning literature. Because unknown data would be expected to result in more misclassification than known data points, training data error is generally considered to be an overly optimistic estimate of performance [39,48,49].

**Fig 3:**
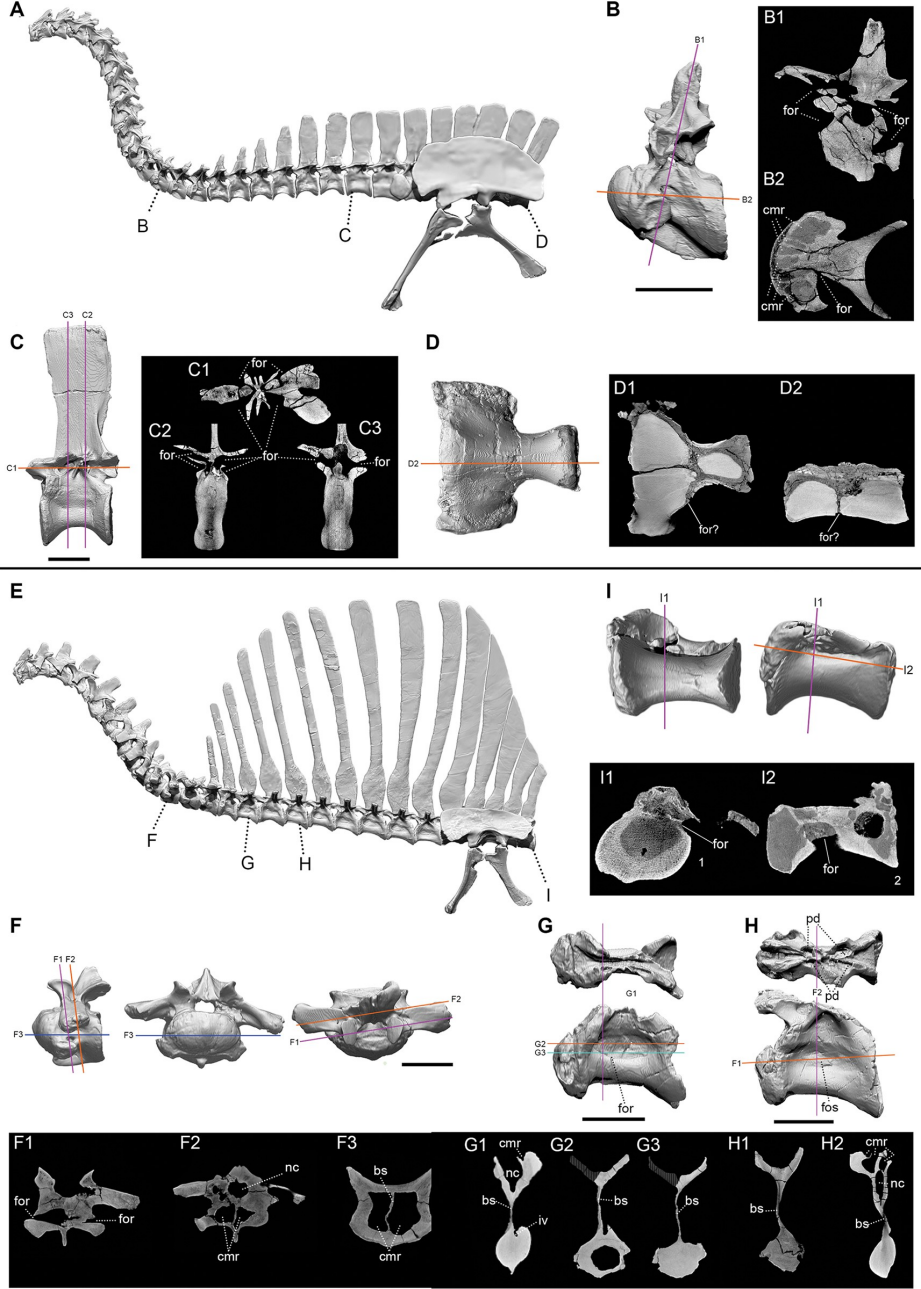
Pneumatic features in the dorsosacral column in spinosaurids. (A) *Suchomimus tenerensis* (MNBH GAD500) precaudal column and pelvic girdle showing pneumatic features in (B) D2 in lateral view with coronal (B1) and axial (B2) CT cross sections, (C) D13 in lateral view with axial (C1) and sagittal (C2, 3) CT cross sections, and (D) S2 in ventral view with axial (D1) and coronal (D2) CT cross sections. (E) *Spinosaurus aegyptiacus* precaudal column and pelvic girdle showing pneumatic features in (F) ~D2 in lateral, anterior, and dorsal views with coronal (F1, 2) and axial (F3) CT cross sections, (G) ~D6 in dorsal and lateral views showing coronal (G1) and axial (G_,_2, 3) CT cross sections, (H) ~D8 in dorsal and lateral views with axial (H1) and coronal (H) CT cross sections, and (I) S3 centrum in ventral and lateral views with coronal (I1) and axial (I2) CT scan sections. Neotypes FSAC-KK-11888 (panels G, H, I) and BSPG-2006-I-54 (panel F). CT section lines are color-coded by orientation (*magenta*, coronal; *blue*, axial-horizontal; *black*, sagittal/parasagittal). Scale bars are 10 cm. Abbreviations: bs, bony septum; c, cervical vertebra; cmr, camera; d, dorsal vertebra; for, foramen; fos, fossa; nc, neural canal.

Fabbri *et al*. mentioned classification performance only in this passage [15: 856]:

The correct classification rates of our phylogenetically flexible discriminant analyses ranges are 84–85% (femora) and 83–84% (ribs) (Figs 2 and 3, Supplementary Materials, Supplementary Tables 7–10). This increases to 90% in both datasets when excluding graviportal and deep diving taxa (Figs 2 and 3, Supplementary Tables 7–10).

The Supplementary Tables 7–10 they cited in the passage do *not* contain correct classification rates, and the definition of “correct classification” is highly ambiguous because the work did not specify which of the multiple classification performance metrics were used (see S1 Appendix, section 4). The referenced tables contain median *P*_2_ values for the dinosaur test taxa, including the spinosaurids, but not for any taxa of known class in the training datasets. They therefore cannot be used as a basis for a correct classification rate. A defined metric of training data errors, known as accuracy and denoted here as *A* (Eq (6) in S1 Appendix), can be derived from output from Schmitz’s and Motani’s pFDA base-layer code [20]. Thus, it is plausible that Fabbri *et al*. used the accuracy metric *A* when they computed the 83–85% correct classification rates, but we cannot rule out the use of some other, undescribed metric.

Correct classification of 83–85% implies a misclassification rate of 15–17%. This reflects performance that seems, on its face, at least three times worse than the usual 5% threshold for random results in statistical methods. Such a result would normally be considered not statistically significant. However, the assessment of the error in classification is complicated by the fact that a classifier that makes a constant guess (*i*.*e*., *P*_2_ = 1.0 for all points, or *P*_2_ = 0 for all points) will be correct 50% of the time if the test taxa are equally distributed between the two classes. So will a classifier that makes random guesses. Yet neither a constant nor a random classifier would have any scientific value.

This effect suggests a useful thought experiment, in which we consider a mathematically equivalent case (with respect to overall classification performance) where the classification is completely random with probability *P*_rand_ and correct with probability 1−*P*_rand_. In such a case can interpret accuracy *A* as an estimate that the classification is correct, *P*_class_, so *A* = *P*_class_ = 0.5*P*_rand_+1.0(1−*P*_rand_), which reduces to

$$P_{\mathrm{rand}}=2(1-P_{\mathrm{class}}).$$ (4)

Applied to the case above with *A* = 0.85—an accuracy of 85%, comparable to that claimed by Fabbri *et al*.—we find that *P*_rand_ = 0.3. Thus an 85% “correct classification rate” means that the classification is mathematically equivalent in performance to the classification being random 30% of the time and correct 70% of the time. This is *six times* the conventional threshold of 5% for the effect to be due to randomness. Such a result would not typically be considered strong enough to warrant a scientific conclusion.

Heuristically, the conventional threshold of 5% can be cast as *P*_rand_≤0.05, which is equivalent to *A*≥97.5% by Eq (4). Because training set *A* is an overly optimistic measure of classification performance, this still is a very loose criterion. Eq (4) provides an important heuristic, which we use in this study to assess the degree of randomness in classification. However, in all cases we present the actual numerical values of the 95% CI on *A* and *P*_rand_, as well as two other classification metrics, *B* and *MCC*, that are defined in S1 Appendix.

The accuracy *A* simply tallies up incorrect classifications and divides by the size of the training set (Eq (6) in S1 Appendix). Many classifiers make a systematic distinction between false-positive errors—which classify class 1 datapoints in the training as class 2—and false-negative errors, which make the inverse mistake. That difference, and many other factors, introduce complications in characterizing classifier performance. The development of classification metrics for statistical classifier algorithms such as pFDA is a very active area of statistical research, with practical applications in areas such as medical diagnostics. Although the topic is beyond the scope of the present work, S1 Appendix sections 2–4 introduce the basics.

Adding an unfortunate complication, we discovered a flaw in the pFDA code that systematically misstates the confusion matrix from which classification performance is measured (S1 Appendix, section 5) by reporting a matrix that is the transpose of the confusion matrix, as it is typically laid out in the literature. Our replication attempts produce classification rates slightly different from those reported by Fabbri *et al*. This issue may be why they do not match exactly.

To judge the performance of classification in this study, we employ two heuristics. One method is to inquire whether the lower bound of the 95% CI for *P*_2_ is above 0.95. That tells us whether the prediction for a single taxon has strong support. This heuristic is predicated on the assumptions of pFDA that the classes are normally distributed with different means and the same variance.

The second approach empirically measures how often the classifier correctly or incorrectly classifies its own training dataset, quantifying its success with metrics such as the accuracy *A* and others (*B* and *MCC*) described in S1 Appendix. We then convert those results to the heuristic metric *P*_rand_ (Eq (4)), the probability that the classifier acts randomly. *P*_rand_ is an overall metric of the classifier, specific not to a particular taxon but to the entire set of taxa in the training set. By characterizing the performance of the classifier on known cases, *P*_rand_ helps calibrate the confidence we should have when using the classifier to extrapolate unknown cases.

Having two different approaches begs a question of how they interact with each other. Unfortunately, the answer awaits further research in statistics. Uncertainties of this kind are the price one pays for attempting to use a statistical method that was never intended to provide the primary statistical evidence for a scientific conclusion.

## Results and discussion

Our examination of the analysis of bone compactness to infer spinosaurid behavior included a critical assessment of several aspects of this methodology. We identified a number of substantive issues that constrain the inferential utility of the method, ranging from logical and statistical problems with regressions based on the *Cg* metric to accounting for uncertainty in those measurements that arises from quantification techniques and biological variation within and among specimens. We describe these issues, along with results from our attempted replication of *Cg* measurements reported by Fabbri *et al*., in the following subsections.

We also identified more general issues with the application of pFDA to data of this kind and to inferences about the behavior of extinct taxa such as dinosaurs. Additional subsections below present our findings on the effects of training-set sample size and selection criteria and demonstrate how researchers can test whether training sets meet the distributional requirements of the pFDA method, again focusing on the recent study of Fabbri *et al*. as a noteworthy example.

### *Cg* and the bone ballast hypothesis

One of the two independent variables in the datasets analyzed by Fabbri *et al*., as well as in this study, is global compactness *Cg*, a longstanding numerical metric of bone microanatomy describing the amount of bone present in a given cross-sectional slice. Because of its effects on buoyancy, bone density is the primary biological parameter of interest, and as *Cg* correlates to bone density, it has been widely used as a proxy for density in the literature [18,50,51]. Fabbri *et al*. used “*Cg*” interchangeably with “bone density” in their study.

It is worth noting that “bone density” in this context refers to the density of whole bones, not to the density of the bone material itself. For example, *Cg* and whole bone density are both low in flying birds and bats, whereas the actual bone material when studied in isolation is quite dense [51].

The *Cg* metric is only one of many available to capture bone microanatomy. Over the last decade, other metrics generated by the Bone Profiler program have been shown to better correlate with lifestyle in studies of extant amniotes than *Cg* does [52,53]. The additional metrics were not used by Fabbri *et al*. and are beyond the scope of the present study.

Fabbri *et al*. signaled their interest in bone density as a marker of lifestyle when they stated that increased bone density “results in increased body density, facilitating buoyancy control during subaqueous immersion related to either submerged aquatic foraging (for example, in underwater pursuit divers), concealment or refuge” [15]. The idea that bone density can act as ballast helpful to certain secondarily semiaquatic taxa is well-studied in the literature, where it is sometimes termed the “bone ballast hypothesis” [54]. However, Fabbri *et al*. misstated and oversimplified the long literature on the bone ballast hypothesis in the quote and elsewhere in their study.

Increase in bone density occurs by pachyostosis, which involves an increase in dense peripheral bone deposits, and/or by osteosclerosis, which involves an increase in bone deposition toward the center of the medullary cavity of long bones [50,54]. The potential advantages for semiaquatic and fully aquatic tetrapods are known to depend greatly on lifestyle: denser bones lead to a denser body, which can facilitate diving and compensate for larger lung capacity, but the increased mass also makes animals less maneuverable [50,54]. As Taylor summarizes [54],

These features are useful for slow swimmers and shallow divers, such as feeders on benthic plants and invertebrates. Examples are sirenians, primitive sauropterygians (“nothosaurs”), placodonts, and the sea otter *Enhydra*.

Taylor and other researchers [50] have found that lifestyles other than those noted in the quote above are *not* compatible with increased bone density, as evidenced by the fact that increased bone density is typically not found in fast swimmers or pursuit predators.

The statement by Fabbri *et al*. conflates behaviors in which increased bone density does offer an advantage—*i*.*e*., “buoyancy control during subaqueous immersion”—and behaviors in which it may or may not apply (“concealment and refuge”) with “underwater pursuit divers,” which the literature makes clear are *not* helped by increased density and indeed are found to have lower density and *Cg*. This conflation is directly contradicted by Taylor, as well as by Houssaye’s review [50], which Fabbri *et al*. erroneously referenced in support of their position.

The relationship between bone density, or its proxy metric *Cg*, and semiaquatic or fully aquatic taxa via the bone ballast hypothesis is thus not simple [44,52,55,56]. The fully aquatic sirenians *Dugong dugong* (*Cg* = 0.994 in ds2) and *Trichechus manatus* (*Cg* = 0.977 in ds2) have very dense bones, which reduce energy expenditure while foraging underwater vegetation. The sea otter *Enhydra lutris* (*Cg* = 0.908 in ds2) must dive for shellfish, which rarely require pursuit. The lower *Cg* of Bryde’s whale *Balaenoptera brydei* (*Cg* = 0.611 in ds2) is consistent with their fast pursuit of prey, and the semiaquatic seal *Phoca vitrulina* has an even lower value (*Cg* = 0.436 in ds2) [44,50,52,54]. Fabbri *et al*. assigned all these example taxa to the *F0D2* class, despite their important lifestyle differences.

#### Regression analysis to explain *Cg*

Fabbri *et al*. performed a PGLS regression analysis to estimate how well values of the categorical variables *F* and *D* explain *Cg* in their datasets [15: Table 1]. One might expect *Cg* to be the independent variable and *D* the dependent variable to interrogate whether *Cg* predicts lifestyle. Indeed, the subsequent pFDA uses both *MD* and *Cg* as the independent variables; lifestyle (*i*.*e*., class membership) and thus *F* and *D* are implicit dependent variables. For reasons that are not explained, the PGLS regression does the opposite: it treats *D* as an independent variable and *Cg* as the dependent variable. Correlations in a linear regression apply in either direction, so we find no substantive impact of this choice.

Fabbri *et al*. reported statistically significant but very weak correlations in both femoral and rib datasets between *Cg* and their subaqueous foraging category *D* = 2. In the femoral data, *Cg* values range from 0.279 to 0.989, a difference of 0.71. With a coefficient of determination *R*^2^ = 0.172, we would expect about 17.2% of the total variation in *Cg*—or an absolute difference of 0.122 in *Cg* across the full range of values—to be attributable to “subaqueous foraging.” In the rib data, *Cg* ranges from 0.242 to 0.998, a difference of 0.756, and *R*^2^ = 0.108, with subaqueous foraging explaining about 10.8% (0.082) of the total variation in *Cg*. One possible reason that the effect is small is that the datasets they used for the analysis were not well chosen to test the bone ballast hypothesis, as both datasets grouped together high-*Cg* and low-*Cg* diving taxa.

Fabbri *et al*. interpreted their regressions results as confirmation that “frequent subaqueous foraging is associated with increased femoral and rib density across amniotes.” Our results show that this greatly overstates the case and contradicts the literature. Prior studies have made it clear that the bone ballast hypothesis is not some sort of universal law of nature across amniotes but has many exceptions, and that a number of other ecological and lifestyle factors may play roles in increased bone density [44,46,50,52,54–56].

In addition to the examples outlined above and in the literature, detailed study within some lineages has shown that the bone ballast hypothesis has its limits [54]. For example, within talpid moles, which include fossorial (burrowing) as well as terrestrial and semiaquatic forms (including semiaquatic desmans in the datasets used by Fabbri *et al*.), no correlation has been found between lifestyle and *Cg* measurements from the humerus [57]. It is thus misleading for Fabbri *et al*. to characterize the regression result as empirical evidence for a general bone ballast rule across amniotes. The dataset is not sufficiently comprehensive, nor are there tests for semiaquatic or aquatic clades that might violate the rule, examples of which Fabbri *et al*. included in their datasets. Simply because an aggregate characteristic of a group (such as the regression result) holds does not imply that one can draw a conclusion about every member of the group—doing so is an example of the ecological fallacy (see S1 Appendix, section 1).

A lesson for future studies is that great care must be taken when drawing sweeping conclusions, particularly if they are contradicted by the available literature or miss large groups that are central to the analysis.

#### Unsupported inferences about subaqueous foraging

The essence of the pFDA method is that each member of a class must share a property, as detailed in Materials and methods. If the members of the class do not actually share the property of the class, valid inference from the classification is limited. Fabbri *et al*. claimed that *F0D2* is the class of nonflying animals that practice subaqueous foraging. Our examination finds that this is not the case.

Their paper does not formally define “subaqueous foraging” but distinguishes it from other aquatic lifestyles [15]:

Secondary adaptations to aquatic lifestyles, such as wading behaviour (shoreline specialist and/or only partially submerged habit), subaqueous foraging (fully submerged behaviour) and deep diving, evolved multiple times in every major amniote group.

In context, the term appears to mean foraging while fully submerged, in contrast to a shoreline-oriented terrestrial or wading species that is only partly submerged while foraging. The submergence, or lack thereof, clearly applies to the forager, rather than to the prey or plants being eaten. A subaqueous forager is thus either a habitually diving predator in pursuit of underwater prey, such as an otter or seal, or a habitually diving herbivore that feeds on underwater plant resources, such as a manatee. Essentially any foraging that occurs fully underwater seems to be included.

Yet the datasets that Fabbri *et al*. presented [15: Table 2] include taxa in the subaqueous forager category that *do not* forage underwater, such as the common hippo (*Hippopotamus amphibius*) [58], pygmy hippo (*Choeropsis liberiensis*) [59], common tapir (*Tapirus terrestris*) [60], Malayan tapir (*Tapirus indicus*) [61], beaver (*Castor fiber*) [62], and European water vole (*Arvicola amphibius*) [63]. Although each of these taxa has secondary semiaquatic adaptations to aquatic habitats, they nevertheless forage substantially—in some cases exclusively—on land and above water. These species habitually enter aquatic habitats as refugia to avoid predators, for thermoregulation, or for other reasons not related to foraging.

In a previous preprint [64], we challenged the classification of hippos and tapirs as subaqueous foragers because they do not forage appreciably underwater. Fabbri *et al*. responded that the term “subaqueous foraging” meant habitual “subaqueous submersion” [16],

Although habitual submersion, as epitomized by the frequent use of subaqueous foraging, is only one functionally important aspect of aquatic behaviour, it is the key aspect that we hypothesized as having a functional relationship to bone density.

This clarifies that the shared property used in their study to assign taxa to the *F0D2* class was frequent full submersion, regardless of diet, predatory behavior, or even foraging at all. One surprising result from our inquiry is therefore that the pFDA analysis of Fabbri *et al*. is unable to infer anything about spinosaurid foraging, and that the conclusions about spinosaurid predatory behavior in that study are unsupported.

In their preprint, Fabbri *et al*. acknowledged that their datasets include taxa that do not forage underwater, but they claimed that “these exceptions are strictly related to a specific diet: herbivory” [16]. However, we found that their training datasets include the American mink (*Neogale vision*) [65] and Pyranean desman (*Galemys pyrenaicus*) [66], both of which have carnivorous diets and eat terrestrial prey—almost exclusively in the case of mink—as well as foraging underwater. Both were included in the *F0D2* femoral and rib datasets. The American alligator (*Alligator mississippiensis*) [67,68] and Nile crocodile (*Crocodylus niloticus*) [69] were also misclassified as subaqueous foragers by Fabbri *et al*., despite ample evidence that adult diets of both species consist of mostly terrestrial prey. Though these large alligators and crocodiles use submersion for concealment while stalking animals on the shore, their lunges above the water to capture prey are clearly not “fully submerged behavior” [67,70–75]. These species take prey both in the water and out of it, to differing degrees, but they are not clear exemplars of “subaqueous foraging.” Meanwhile related crocodilian species that better represent obligate subaqueous foraging, such as the gharial (*Gavialis gangeticus*) [76], were not included by Fabbri *et al*.

Crocodiles also illustrate the complex role of ontogeny in functional assignment, as they grow by orders of magnitude and often exhibit dietary change as they mature [73,77,78]. Crocodilians are not fast pursuit predators and instead tend to be lunging ambush predators [79]. They may be insectivores while very small hatchlings, submerged piscivores at moderate size, and then as large adults transition to a diet that includes terrestrial prey. A scheme that does not specify ontogenetic stage cannot correctly classify such species.

To extend the bone ballast hypothesis broadly, one must understand where different ontogenetic stages fit. Currently it is unclear whether we should expect relevant species to show increased bone density (and thus *Cg*) at all stages of their life history, or only as adults. In the latter case, it will be necessary to verify that data was gathered from specimens at the same ontogenetic stage.

This issue is particularly salient for making inferences about spinosaurids because ontogenetic dietary niche partitioning has also been identified in theropod dinosaurs [80–82]. Like crocodilians, predatory dinosaurs spanned a similar or possibly even larger range of body size from hatchling to adult, and they almost certainly accessed a range of size-appropriate prey [80]. Spinosaurids, a group that includes one of the largest theropod dinosaurs yet discovered, are likely to have sought a sequence of preferred ecological niches during ontogeny [13]. It is also worth noting in this context that the neotype of *Spinosaurus* is thought to be an immature specimen that is substantially smaller than other specimens presumed to be fully grown [14].

Equally important to a discriminant analysis is appropriate selection of a sufficient number of representative taxa for the control group that does not show the behavior of interest—the *F0D0* and *F0D1* classes, in the study of Fabbri *et al*. The *F0D1* group contains just 2 taxa, too few for pFDA or any robust statistical analysis. The *F0D0* group omits many large terrestrial species that capture aquatic prey just under the water surface without being submerged themselves, such as brown bears, black bears, and wolves, all of which prey on swimming salmon [83–86]. Jaguars hunt caiman and capybara both above and below water [87–89]. Taxa such as these would seem a good fit for inclusion as nondiving predators that rely on aquatic prey as a substantial, or even critical, component of their diet [90].

Eagles, ospreys, and other raptors—as well as many other birds such as skimmers and egrets—similarly forage while flying by grabbing fish from under the water surface [91–94]. Herons, storks, egrets, and cranes also forage while standing in shallow water or shoreline perches, plunging their head underwater to capture fish and other aquatic prey [95–97]. This model has been proposed for *Baryonyx* [4] and, more recently, for *Spinosaurus* [13]. A token two examples of taxa that forage in this manner are included in the pFDA training datasets to represent “wading or only partially submerged” foraging behavior.

The *F0D2* dataset, in contrast, includes a wide variety of different foraging styles, including slow-swimming aquatic herbivores, predators of stationary aquatic prey (such as the mollusk-eating sea otter *Enhydra lutis*), fast-swimming pursuit predators, and semiaquatic herbivores and carnivores that do not forage underwater. Though all are classified as frequent divers, that interpretation seems odd in some cases, such as hippos, which do not swim but rather stand in shallow water or walk along the bottom [98].

Fabbri *et al*. offered the following justification for this mix of semiaquatic animals and foraging [15]:

Previous studies applied different categorizations for the characterization of aquatic lifestyles among extant and extinct taxa: ‘aquatic’ and ‘semiaquatic’ were used contra ‘subaqueous foraging’ applied in this study. Our ecomorphological attribution is focused on a specific behaviour linked to an ecology, rather than a categorization of its entirety. We find our categorization to be more accurate: for example, previous studies coded penguins and cetaceans as aquatic, while crocodilians were stated as semiaquatic. Whereas penguins and crocodilians are still ecologically dependent on terrestrial environments (for example, for laying eggs), cetaceans are completely independent from land. On the other hand, all these clades engage in subaqueous foraging. Therefore, our ecological attribution is in agreement with previously applied ecological categories, but do not exclude dependency to terrestrial environments to satisfy autecological requirements, such as reproductive behaviour.

Essentially no evidence was presented to support the utility or “accuracy” of substituting “subaqueous foraging” in place of the more traditional characterizations “semiaquatic” and “aquatic.” The examples we have cited above of animals that dive but do not forage underwater and those that forage underwater without diving show this proposition to be false. Their assertion that “all these clades engage in subaqueous foraging” also overstates the case—the datasets comprise exemplar taxa, not clades. Depending on how broadly one construes the clade for each taxon, most of the clades include members that are terrestrial.

We find that even if the analysis was otherwise correct—and below we present further evidence that it was not—the strongest inference one could draw from the *F0D2* classification with regard to *foraging* is that *Spinosaurus* and *Baryonyx* had a statistical affinity with a group of animals that have semiaquatic or aquatic adaptions and display a wide gamut of foraging styles in and out of the water. One could infer that these spinosaurids fully submerged themselves but may not have been able to swim—although further evidence presented in the next subsection contradicts the possibility of full submersion. Such a vague and tenuous inference seems of little import to the controversy over spinosaurid ecology because it hardly improves on the semiaquatic and piscivorous adaptation that has long been suggested for spinosaurs.

We also find from the application of the bone ballast hypothesis, as described in the literature, that the high *Cg* found in *Baryonyx* and *Spinosaurus* suggests that they probably were not fast-pursuit predators, because such taxa do not typically have high *Cg*.

A key lesson for future studies is that it is of paramount importance that exemplar groups in training datasets actually possess the features that they are claimed to have. Further care must be taken so that the interpretation of the statistical results respects both the dataset composition and the theoretical justification behind it.

#### Bone ballast versus axial pneumaticity

The bone ballast hypothesis focuses on the role that ballast has in certain groups and lifestyles for secondarily semiaquatic and aquatic amniotes. However, the hypothesis is based on analysis of extant taxa that lack skeletal axial pneumaticity, except for birds [99]. In this subsection, we present results of our investigation into the important question of how the bone ballast hypothesis applies to animals that have significant pneumaticity, a question highly relevant to inferences about spinosaurids.

Pneumaticity in theropods, including *Spinosaurus*, has a strong effect on body density because pneumatic invasion replaces soft tissue or bone, which has density ranging from 1.0 to 1.2 g/ml, with air that is a thousand-fold less dense—about 0.0012 g/ml at sea level and 15°C. Cancellous or dense bone infilling, by comparison, replaces soft blood vessels/marrow of density near that of water (1.0 g/ml) with bone of only slightly greater density (~1.2 g/ml). Pneumaticity in the axial and appendicular bones in theropods (including birds) thus *increases* buoyancy by roughly 5 to 6 times more than a comparable volume of “dense” bone *decreases* it.

Studies of pneumaticity in birds as a correlate to lifestyle show that pneumaticity is positively correlated with body mass in flying birds; heavier birds have higher pneumaticity [100]. Pneumaticity has been lost in multiple lineages of diving birds [99]. Smith performed phylogenetic regressions to show that the pneumaticity is strongly correlated with lifestyle among water birds [101]. Pelicans feed primarily at the surface without complete submersion or only shallow diving, but they are not found to be apneumatic in any of the analyses. They are, in fact, highly pneumatized [99]. However, there is a strong correlation between decrease or loss of pneumaticity and pursuit diving in birds, a correlation seen both in flying taxa (loons, grebes, darters) and in flightless species, such as penguins [101].

Loss of pneumaticity, or reduction in its degree, increases body density and acts as ballast, a fundamental biomechanical effect compatible with the bone ballast hypothesis. However, density acquired through loss of pneumaticity may reduce or obviate the need for bone density increase from denser long bones and ribs. The lesson from birds is that the bone ballast hypothesis is most strongly observed in reductions of pneumaticity rather than pachyostosis or osteosclerosis of long bones and ribs.

Pneumaticity in birds is relevant to spinosaurids because spinosaur bone structure provides ample evidence of vertebral pneumaticity, which would supersede any ballast effect from variable infilling of long bones [8,13,14]. Spinosaur fossils also exhibit large medullary cavities (presumably filled with fat during life) that hollow the centra at the base of the tail and would have further reduced bone density [14]. The internal volume of cervical pneumaticity (~25% by volume) is well documented in *Spinosaurus* [102], with evidence that the entire dorsosacral column is pneumatized (Fig 3). In *Suchomimus* and *Baryonyx*, most precaudal vertebrae have internal pneumatic chambers (camerae) within the centra and deep fossae likely for pneumatic diverticulae on the neural arch (Fig 3B–3D).

Precaudal vertebral pneumaticity is present in *Spinosaurus* to an even greater degree than in its baryonychine relatives *Baryonyx* and *Suchomimus*. The pneumatic foramina and camerae in the anterior dorsal vertebrae (Fig 3F) are larger than in *Suchomimus*, and mid-dorsal centra have marked, oval pneumatic fossae that reduce intervening bone to a thin sagittal septum (Fig 3G and 3H). Similarly, midsacrals have large pneumatic foramina and internal camerae (Fig 3I).

The bone ballast hypothesis relies on bone density influencing overall body density. In a mammal or reptile, it may be reasonable to infer a trend from a sample of rib and/or femur density (or a proxy such as *Cg*), if one assumes that the sampled bone’s density is representative of a trend followed by other skeletal elements. In a bird or dinosaur with vertebral pneumaticity, however, this is not the case. The contribution of the skeletal elements to overall body density depends on both their density and their volume. The impact of the air sacs involved in pneumaticity, for example, depends on the total volume of the air sacs. One cannot infer the degree of pneumaticity by sampling skeletal elements in which is not present. Even sampling bones that do exhibit pneumaticity does not allow computation of the buoyancy effect unless the total volume of those bones is also measured or estimated.

The quantitative impact of vertebral pneumaticity in *Spinosaurus* is so strong that calculations of body density from 3-D flesh models have found specimens of this taxon to be unsinkable [8,14]. In water, the buoyancy of the air sacs and pneumatic diverticulae would exert an upward force so strong that not only would it exceed any plausible ballast effect of dense ribs and femurs, but it also could not plausibly have been overcome by thrust generated from the tail and/or limbs [14]. *Spinosaurus* could not have fully submerged.

If the evolutionary pattern found from the analysis of multiple clades of extant diving birds—that fast-swimming pursuit diving is correlated with reduced pneumaticity [101]—holds for theropod dinosaurs as well, then the extensive vertebral pneumaticity in *Spinosaurus* can be seen as evidence to reject the fully aquatic pursuit predator hypothesis.

By focusing solely on *Cg* in femora and ribs, the analysis of Fabbri *et al*. was effectively blind to vertebral pneumaticity, the most important factor for the bone ballast hypothesis in birds and dinosaurs and a key difference that distinguishes these groups from mammals and reptiles. We find that the omission of pneumaticity causes classification by femoral or rib data alone to be misleading, and any inferences drawn from such analysis to be invalid.

Integrating pneumaticity into a future study would be possible, in principle. Quantitative data on pneumaticity is available for many taxa of extant birds [100], and studies have started tracking pneumatic diverticulae via CT scans [103,104]. Integrating pneumaticity into a *Cg*-based study of the bone ballast hypothesis could be difficult in practice, however. If *Cg* has a direct correlation to body density, it can be used as a proxy—but only if other contributing factors to body density (flesh density, lung volume, etc.) do not confound the correlation, as is generally thought to be the case for reptiles and mammals. In any animal that has significant skeletal pneumaticity, the confounding effect of the pneumaticity is strong and not captured by *Cg*, even if *Cg* is measured for the skeletal elements in question. Instead of relying on *Cg* as a proxy for body density, one would need to quantify the relative impact on buoyancy of increased *Cg* in some skeletal elements—including long bones and ribs, but possibly others as well—while also accounting for the total volume of the bones as compared to the total volume of air sacs. Such a calculation is difficult to make because it depends on accurately estimating the volumes of bones, flesh, and air sacs. Rather than simply measuring *Cg* in femurs and ribs, a study that accounts for pneumaticity would need to make detailed, accurate 3-D models of the entire skeletal, flesh, and air volume structures for every taxon in the dataset.

#### Body mass confounds classification based on *Cg*

We examined the possibility that lifestyles and body characteristics other than buoyancy may act as confounding factors in a classification based on bone compactness, biasing the results if they are not properly controlled in the statistical analysis. Our review of relevant literature found several plausible confounders. Burrowing animals may have increased *Cg*, particularly in limbs used for digging [105]. Some arboreal groups, such as sloths, also have increased *Cg* [106]. More relevant to spinosaurids, body mass has also been associated with bone density. Studies of large-bodied terrestrial taxa often have reported increased *Cg* [52,53,55,56,107–111].

The effect of large body size was well considered in the common hippo by Houssaye *et al*. [52]:

However, it is difficult to determine whether the pattern observed in *Hippopotamus* reflects its graviportal limbs or the benefit of a slight increase in bone mass in its legs enabling their use as ballast and offering stability in water. As a result, both adaptations might be mistaken, or even synergistic, and it seems almost pointless to try to unravel their evolutionary integration. Adaptation to a graviportal limb morphology should thus be taken into consideration when analyzing possibly amphibious taxa displaying a terrestrial-like morphology, and thus notably in the study of the early stages of adaptation to an aquatic life in amniotes.

*Spinosaurus* and other spinosaurids are in the top tier of body mass among theropods [14], so the potential for increased *Cg* as a consequence of large body size must be considered as a viable alternative to the bone ballast hypothesis as an explanation of the observational data. Future investigations could add a separate categorical variable for large body mass to see whether that improves the classification of test taxa, for example. But the admonition above to take adaptation to large body size into consideration was explicitly *not* heeded by Fabbri *et al*., as their approach forced large-bodied taxa into either the *F0D0* or *F0D2* classes—*Hippopotamus* was assigned to the latter.

Large-bodied taxa, such as the African elephant *Loxodonta*, which has *Cg* comparable to *Baryonyx*, and other extant (Asian elephant, rhinoceros) and extinct (mammoth, extinct hippos) mammals, were included in ds1 and ds2 but were not separated into a separate class to facilitate comparison or control of the confounding factor. Most large-bodied taxa were purged from the the ds3 and ds4 training datasets under a flawed rationale, which we examine in the next subsection. Large-bodied dinosaurs in their *Cg* datasets were classified as “*D* = unknown,” thereby excluding them from the training set; all non-avian dinosaurs were used only as test taxa.

The analysis may also have been confounded by inclusion in the datasets of many taxa that are small, even minuscule: the smallest have femoral diameters <1 mm and body masses ≤7 g. *Spinosaurus* achieved masses approximately 10^6^ times larger [14]. In ds1, the median femoral diameter is 12.08 mm; half of the dataset has a *smaller* diameter. The ds1 exemplar taxon with the femoral diameter closest to the median is *Taxidea taxus*, the American badger. Typical body mass of this taxon is 6–9 kg; *Spinosaurus* weighed roughly 1000 times more. No argument was provided to justify the use of such small-bodied taxa as biomechanical exemplars for spinosaurs.

Disparities between classes, which can influence classification, are notably large in their study. The median across the femoral *F0D0* class is 11.5 mm (*Meles meles*, the European badger); that is only about 60% of the median of the *F0D2* class, which is 19.1 mm (*Neusticosaurus*, an extinct pachypleurosaur). In an LDA analysis, such a disparity would strongly bias the decision boundary toward lower *Cg* values for taxa that have *MD* greater than the centroid values. We examined whether the adjustment for phylogenetic bias by pFDA mitigates this bias and found that it does not, as detailed in the next subsection.

Another possible confounding factor for the bone ballast hypothesis is ballast by other means—such as ingesting gastroliths, a behavior known to occur in crocodilians, plesiosaurs, and possibly others [54,112–114]. If swallowed stones provide ballast, skeletal modifications may not be adaptive for diving predators. Evidence exists that some clades of nonavian dinosaurs, including theropods, used gastroliths [115,116]. But the more salient confounding influence would be the presence of gastrolith-dependent taxa in the training sets [116]. Further consideration of gastroliths is beyond the scope of the present study.

We find that the combined impact of the limitations described above leaves increased *Cg* in *Spinosaurus* unexplained: it may be a secondary semiaquatic adaptation (under the bone ballast hypothesis), a consequence of its large body size and/or body mass, or conceivably a combination of the two, as with hippos. In limiting their analysis to the classes “subaqueous foraging” versus not and ignoring plausible confounders such as pneumaticity, body mass, and bone strength or stiffness—considered a biomechanical correlate to bone density in the literature for other taxa [117–119]—Fabbri *et al*. failed to account for other possibilities.

Any future study seeking to use the bone ballast hypothesis for clades of dinosaurs which have pneumaticity must address the confounding effect of body size on *Cg*. Other possible confounding factors should also be investigated and tested via statistical methods to confirm that they are not the cause. The taxa chosen should not be widely disparate between classes in key biomechanical attributes, including body size—especially if a proxy for body size, such as *MD*, is one of the variables.

### Issues with dataset composition

Separate from the data selection issues that involve the bone ballast hypothesis, our examination identified several problems related to dataset composition. We show below that one of the variables used by Fabbri *et al*. should have been omitted, as it reduces the statistical power of their analysis. We also found that removal of deep-diving and graviportal taxa from the training datasets was performed inconsistently and using subjective judgments that appear unjustified. This subsection addresses these issues, and their consequences, in turn.

#### Unnecessary inclusion of *MD* in the analysis

The justification for using *Cg* as an independent variable is based on the bone ballast hypothesis explored above. We turn now to the use of *MD* (in the form of log_10_(*MD*)) and why it is included. One possible reason would be to explicate the role of body mass, using femoral diameter as a proxy. This reasoning is undercut by several factors: the lack of suitable large body mass exemplars in the ds1 and ds2 training sets; the removal of most of the exemplars that could serve that purpose in the ds3 and ds4 training sets; and the fact that the training sets are biased to include taxa that have much lower body mass than the test taxa do.

In their initial round of analysis, Fabbri *et al*. used PGLS to test whether the categorical variable for “subaqueous foraging” predicts *Cg*. As a follow up, they performed a phylogenetic ANOVA with all possible pairs of variables, including “subaqueous foraging” and *MD*, as well as “subaqueous foraging” and flight, to see which combinations predict *Cg* best (details in Materials and methods).

The results of including *MD* and *Cg* in the regression were described, and values of the Akaike Information Criterion (AIC) were compared with a regression that included *Cg* alone [15]:

Models that include flight or shaft diameter as additional covariates receive less support from AIC (Table 1, Supplementary Tables 3 and 4). This indicates that evidence for an amniote-wide common allometry in bone density, or for association of flight with decreased skeletal density aquatic adaptation (see Table 1, S8 and S9 Figs, Supplementary Tables 3 and 4).

A model that has less support from AIC is one that offers lower explanatory power. Their calculations show that, for the femur dataset, the model that includes subaqueous foraging and *Cg* and *MD* has (under the assumptions behind AIC weights) about *49 times less* explanatory power than a model that includes subaqueous foraging and *Cg* without *MD*. For the rib dataset, the model including *Cg* and *MD* similarly has AIC weights 34 times smaller than those of the model that includes *Cg* without *MD*. (Their position on evidence for “common allometry” remains unclear because the sentence seems to have been truncated in publication.)

Conventional statistical analysis would not further consider *MD* after results such as these that show that adding *MD* dramatically *reduces the model’s explanatory power*. Fabbri *et al*. proceeded to use the inferior model nonetheless, possibly because pFDA requires at least two independent variables. After *Cg*, *MD* is the best-performing of the remaining variates. We find that the analysis should have switched to one of the many statistical methods that could be used to analyze *Cg* alone, recognizing that pFDA is not an appropriate choice, for this reason as well as others we have noted above.

The AIC results also show that the variable *F* also decreases the explanatory power of the model, which should have constrained the class comparison more tightly to *F0D0* versus *F0D2*. Instead, Fabbri *et al*. used the statistically inferior comparison between “subaqueous foraging or not,” leaving the flying taxa (*i*.*e*., *F* = 1 and *F* = 2) in the analysis, as is clear from the data files listed in Table 2 and provided in our Supporting information.

Statistical arguments aside, it is puzzling that a pFDA analysis classifying spinosaurs and other nonavian dinosaurs included flying taxa in its training datasets. None of the dinosaurs in the test taxa has been proposed as being able to fly, so flying taxa are not reasonable biological analogs for *Spinosaurus*. The inclusion of irrelevant taxa risks adding both random and possibly non-random variation that confound the correlations—indeed, the weak AIC results show that it did have that effect. If this variation is unequally distributed across the two classes in the classification, this could bias the classification.

Like many of the other issues raised in this study, the use of models proven to have less statistical evidentiary power is sufficient on its own to call the whole analysis into question. The lesson for future studies is to follow the evidence. If including a variable in the analysis reduces explanatory power, do not use it. The point of using AIC or related criteria to compare models is to identify counterproductive variables so that they can be avoided.

#### Removal of “deep diving” taxa

We found that Fabbri *et al*. misstated that the bone ballast hypothesis holds for all amniotes. A later section of their paper concedes this point [15]:

Deep diving animals, such as ichthyosaurs, mosasaurs, living cetaceans and seals, are characterized by lower bone density when compared to shallow-water subaqueous foragers: the compact bone cortex of deep divers is replaced by cancellous bone characterized by extensive trabeculae and vascularization.

They attempted to address the inconsistency by manually removing such taxa from dataset ds1 and ds2 to create the smaller datasets ds3 and ds4, which they analyzed separately [15]:

High bone density is therefore an excellent indicator for the initial stages of aquatic adaptation, but poorly distinguishes between wading, deep diving, and terrestrial habits. These limitations can be overcome using anatomical observations because deep diving shows other transformations of the body plan, such as presence of fins and flippers.

Fabbri *et al*. did not code their datasets to include stages of aquatic adaptation. We therefore find that the data cannot be used to infer a correlation with *Cg* that is limited to some stages of aquatic adaptation but not others. Although in principle such a study could be done, they neither performed it themselves nor referenced such a study by others, leaving their statement without any support. Their statement ignores complexities that could confound the approach they suggest, such as the fact that sirenians have high *Cg* but would be difficult to characterize as in the “initial adaptation” to aquatic life, considering that they have already lost their legs. If high bone density (and thus *Cg*) cannot reliably discern subaqueous foragers from wading, deep-diving, and terrestrial taxa, then it is unclear how it could serve as an “excellent indicator” of the early stages of adaptation because wading, diving, and terrestrial taxa are the primary alternatives to compare against.

We find that *any* finned or flippered taxa are poor choices as exemplars for comparison to spinosaurids, which manifestly *do* have terrestrial limbs, and should not have been included in the dataset. Even if one supposes that spinosaurids were on an evolutionary trajectory to become fully aquatic (a highly speculative idea, as no fully aquatic descendants have been discovered), the best points of comparison would still be other taxa that have terrestrially useful limbs and are at an early stage of secondary aquatic adaptation. The datasets that Fabbri *et al*. assembled do not feature such taxa.

The literature on the bone ballast hypothesis does not support deep diving as the sole or primary correlate of low *Cg* in secondarily aquatic taxa. Instead the focus is on low *Cg* among taxa that are fast-swimming and pursuit predators and on high *Cg* among slow swimmers, herbivores, and bottom walkers [50,54].

The vague criteria for removal described by Fabbri *et al*. are not mutually exclusive; air-breathing deep divers are often fast swimmers in order to reach depth while holding their breath, and they are often (but not always) pursuit predators as well. Examples include most cetaceans and extinct ichthyosaurs, plesiosaurs, and their kin. Moreover, not all taxa that show the anatomical features identified were removed by Fabbri *et al*. from their analysis. We compared their unpublished data files (S3 and S4 Files) to the tables that list taxa that were eliminated as deep divers [15: Supplementary Tables 5 and 6]. We found that one of the published tables is incomplete, omitting two taxa that were removed from the rib dataset (see Materials and methods). Our replication study confirmed that Fabbri *et al*. used the data files, not the published tables, to produce their results.

We also found that some taxa listed as deep divers in the data files do not meet the anatomical criteria that Fabbri *et al*. specified: transformations of the body plan or the presence of fins and flippers. The extant cetaceans Bryde’s whale *Balaenoptera brydei* and orca *Orcinus orca* were classified as deep divers and thus removed from ds3 and ds4, whereas the flippered extinct whale *Basilosaurus* was not listed as a deep diver and was thus retained in the analysis. The extinct seal *Callophoca obscura* was removed, yet the extinct seal *Nanophoca vitulinoides* was retained. The plesiosaur *Cryptoclidus* was retained, despite its flippers and recent work suggesting an open-ocean lifestyle for many plesiosaurs [120]. More generally, we found that data for sirenians, plesiosaurs, ichthyosaurs, and nothosaurs were retained, despite their flippers, fins, and flukes. So were penguins, even though they have flippers and are deep divers—*Aptenodytes* being known to routinely dive deeper than 400 meters if required for foraging [121,122]. On the other hand, the extinct *Desmostylus hesperus* was removed, despite being a quadruped, with no fins or flippers. We found no suggestions in the literature that this species engaged in deep diving.

We found that the taxa flagged as deep divers by Fabbri *et al*. do all share a feature that, were it used as a deselection criterion, would have biased the analysis: they are the members of the *F0D2* group that have the lowest *Cg* values. We also found that taxa that should have been removed by their criteria but were retained without explanation have high *Cg* values. *Cryptoclidus*, for example, has *Cg* = 0.97 and *MD* = 84.08, almost the same values as *Spinosaurus* (*Cg* = 0.968, *MD* = 81.52).

We find that the removal of low *Cg* taxa from the *F0D2* group, and the retention of high *Cg* taxa that meet the anatomical criteria for removal, increased the contrast between *F0D0* and *F0D2* and biased the classification of spinosaurs toward *F0D2*. Removing data points simply to improve the appearance of correlations is a form of data manipulation that violates standards of statistical practice.

Fully aquatic taxa with fins and flippers should not be used as points of comparison for spinosaurs, which had functional legs and feet. The bone ballast hypothesis does not suspend normal critical judgement about anatomy. It is not a universal rule across amniotes; instead its correct interpretation depends on knowing much more than just *Cg* and *MD*. In particular, taxa that do not use bone as ballast are not simply deep divers—they also include fast swimmers and pursuit predators.

#### Removal of “graviportal” taxa

In addition to removing deep-diving taxa from the ds3 and ds4 datasets, Fabbri *et al*. removed graviportal taxa, which they claimed to select by applying the following criterion [15]:

Graviportal animals can be distinguished from aquatic species by the presence of columnar limbs, an anatomical trait which is generally missing among subaqueous foragers.

They acknowledged in their paper that high body mass may also lead to elevated *Cg*, as we discussed in a previous subsection, but the paper did not explore that likely confounder with statistical tests or other methods.

The term “graviportal” has no universal definition and has been used variously to indicate particular bone length ratios, posture, locomotive mode, and limb articulation [107,111,123,124]. Originally it referred to the relative length of upper and lower limb bones [125], but it is also referred to as a posture or mode of terrestrial locomotion. More recent studies, however, have shown that both posture and locomotor mode lie on a continuum, with position better captured by osteological indices, such as ratios of length to width in long bones [107,108,123,126,127]. Some reserve the term “graviportal” exclusively for a mode of quadrupedal locomotion, whereas others outline criteria for “graviportal bipeds” [110,128–130].

*Spinosaurus* meets those bipedal criteria for being graviportal, consistent with findings that it was bipedal [14]—findings that overturned earlier suggestions that it was quadrupedal [5]. But even if *Spinosaurus* were quadrupedal, limb-ratio tests would classify it as graviportal. *Baryonyx* and *Suchomimus* are both considered bipeds and qualify under the bipedal criteria. Other than their novel and unsupported criterion, Fabbri *et al*. provided no justification for removing graviportal exemplar taxa from a study of graviportal spinosaurids.

Fabbri *et al*. asserted that “graviportality does not affect rib compactness” [15], but our literature review identified two recent studies suggesting that bone density in these two skeletal components are often correlated [46,131].

We find the precise definition of graviportal to be irrelevant to the question of *Spinosaurus* ecology because all large-bodied animals tend to have higher bone density and higher *Cg*, irrespective of their posture or mode of locomotion [53,108,109,132].

As with their removal of deep-diving taxa, Fabbri *et al*. presented a succinct anatomical criterion for removal of graviportal taxa but failed to apply it consistently. Three rhinoceroses (*Ceratotherium simum*, *Rhinoceros sondaicus*, *Rhinoceros unicornis*) were removed from datasets ds3 and ds4, despite having flexed rather than columnar limbs, the ability to gallop, and other nongraviportal characteristics. They do have high *Cg* for a terrestrial animal [52], however. The extinct hippopotamus *Hexaprotodon garyam* was culled, despite distinctly flexed limb postures. The common hippo *Hippopotamus amphibius* was retained (and assigned to the habitual diving group *F0D2*), yet the pygmy hippo *Choeropsis liberiensis* was eliminated as graviportal. The ichthyosaur *Mollesaurus* was also eliminated as graviportal, somehow meeting the criterion for columnar limbs despite having no legs.

If the goal in removing a swathe of taxa, under the dubious rationales of graviportal body type and deep-diving behavior, was to improve the apparent accuracy of the pFDA analysis, the approach had the intended effect. Fabbri *et al*. reported that the correct classification rate improved from around 84% with the complete datasets to 90% [15] for the selectively culled datasets ds3 and ds4. Confidence intervals for the latter analysis widened, however, as a result of the smaller sample size, as we show below in a subsection reporting results from our analysis of the effects of training set size.

We found that the removal of graviportal taxa from the terrestrial *F0D0* class had the effect of removing large-body-mass exemplars from the comparison. That choice of method effectively precluded a proper consideration of a highly plausible alternative hypothesis: that spinosaurs had high *Cg* because they were heavy. The fact that the spinosaurs themselves would be classified as graviportal by current metrics for the condition—and thus eliminated from the analysis—clearly renders the results of that analysis unusable.

#### *Cg* disparity between extinct and extant taxa

Fabbri *et al*. used *Cg* data from a combination of extinct and extant taxa, and they chose an analytical method that implicitly assumes that the statistical distributions of *Cg* are the same for extinct taxa and extant taxa. If the data violate that assumption, that could bias the classification results directly, so one could not draw valid conclusions from the analytical results (see Materials and methods).

In the original pFDA study [21], Montani and Schmitz included only extant taxa in the training dataset that they used to classify extinct test taxa. In that study, however, the test variates were eye-socket dimensions, which have a strong basis in optics, so there was little concern on the matter. In a review of the literature through 2022, we found that all other pFDA studies also made exclusive use of extant taxa for training. Classification of extinct species with an algorithm trained on measurements of extant species is vulnerable to a systematic difference in distribution between the two groups. There is less of an impact on classification if *all* members of each class in the training set have the same status as extinct or extant, however.

In the mixture of extant and extinct exemplar taxa assembled by Fabbri *et al*., the ratio of extinct to extant taxa varies considerably among the training subsets *F0D0*, *F0D2*, etc. We compared the statistical distributions of *Cg* between the two groups and found striking differences in bone compactness between extinct and extant taxa of similar lifestyle in some of the datasets (Table 3). The femoral *F0D2* dataset, for example, shows a strong bias toward higher values of *Cg* among extinct taxa. We ranked the *F0D2* group by *Cg* and found that 20 of the top 21 taxa are extinct (Table 3). The two extant taxa having the highest *Cg* rank 16 and 22 in this dataset (Table 3). The disparity is particularly worrisome because spinosaurids were clearly nonfliers and therefore must either have been nonflying divers (*F0D2*) or terrestrial (*F0D0*).

**Table 3:** Nonflying, diving subset of taxa (*F0D2*) based on femoral data are ranked by bone compactness (*Cg*).

Rank	Taxon	Femoral diameter (mm)	*Cg*	Extant (E)	Extant subaqueous forager
1	*Serpianosaurus*	4.8	0.989	—	—
2	Large Eocene stem penguin	16.744	0.988	—	—
3	*Maiacetus*	30.43	0.985	—	—
4	*Nanophoca vitulinoides*	20.3	0.973	—	—
5	*Cryptoclidus*	84.08	0.97	—	—
6	*Champsosaurus_*	7.85	0.968		
7	*Neusticosaurus*	19.1	0.968	—	—
8	*Phocanella pumila*	29.5	0.966	—	—
9	*Placondontia* indet.	23.38	0.959	—	—
10	***Nothosaurus*_102**	5.168	0.955	—	—
11	*Champsosaurus*	12.389	0.952	—	—
12	Small Eocene penguin	9.457	0.942	—	—
13	*Paraplacodus*	9.05	0.939	—	—
14	***Nothosaurus*_150**	8.125	0.938	—	—
15	*Rhaeticosaurus*	36	0.936	—	—
16	*Caiman yacare*	12.623	0.929	E	yes
17	*Basilosaurus*	21.96	0.926	—	—
18	***Nothosaurus*_568**	5.464	0.909	—	—
19	*Anarosaurus*	10	0.901	—	—
20	*Plesiosaurus*	41	0.90	—	—
21	*Rodhocetus*	26.863	0.893	—	—
22	*Desmana moschata*	5.1	0.89	E	yes
23	*Alligator*	18	0.884	E	no [67,68]
24	*Cricosaurus*	16.265	0.874	—	—
25	*Spheniscus humboldti*	8.06	0.872	E	yes
26	*Ornithorhynchus anatinus*	5.21	0.871	E	yes
27	*Indohyus*	7.44	0.867	—	—
28	*Simosaurus*	22.97	0.865	—	
29	*Aptenodytes*	16.395	0.864	E	yes
30	*Placodontia* indet._1	20.97	0.859	—	—
31	*Lutra vulgaris*	10.02	0.85	E	yes
32	*Chironectes minimus*	4.78	0.849	—	—
33	*Pistosaurus*	27.56	0.845	—	—
34	*Micropotamogale euwenzorii*	2.31	0.844	E	yes
35	*Psephoderma*	9.37	0.843	—	—
36	*Metryorhynchus*	27.384	0.828	—	—
37	***Nothosaurus mirabilis***	16.09	0.828	—	—
38	*Hippopotamus amphibius*	59.34	0.828	E	no [133]
39	*Otaria byronia*	22.28	0.821	E	yes
40	*Palaeospheniscus*	8.52	0.792	—	—
41	*Ichthyosaur sp*.	165.44	0.776	—	—
42	***Nothosaurus mirabilis*_1**	21.7	0.776	—	—
43	*Choeropsis liberiensis*	29.78	0.767	E	no [59]
44	*Remingtonocetus*	35.72	0.765	—	—
45	*Simosaurus*_1	22.9	0.764	—	—
46	*Castor fiber*	29	0.749	E	no [134]
47	***Nothosaurus giganteus***	26.819	0.738	—	—
48	*Callophoca obscura*	25.86	0.733	—	—
49	*Leptophoca proxima*	28.9	0.729	—	—
50	*Neomys fodiens*	0.969	0.729	E	yes
51	*Hexaprotodon garyam*	69.4	0.726	—	no [132]
52	*Hesperornis*	22.914	0.725	—	—
53	*Hydromys chrysogaster*	5.42	0.689	E	yes
54	*Tapirus terrestris*	33.2	0.687	E	no [56]
55	*Protochampsidae*	10.17	0.673	—	—
56	*Ichthyosaurus*	86.48	0.659	—	—
57	*Dyrosaurid*	12.54	0.635	—	—
58	*Phalacrocorax harrisi*	9.26	0.623	E	yes
59	*Desmostylus hesperus*	38	0.596	—	—

The femoral dataset (ds1 of Table 2) used in the analysis of bone density by Fabbri *et al*. [15] includes 59 specimens listed here that are categorized as *F = 0* and *D = 2*. Among the extant species represented (shaded grey), five do not feed underwater exclusively (shaded red). The dataset includes six specimens of *Nothosaurus* (bold), which range in rank from 10 to 47. The top 21 ranking taxa by *Cg* include 20 that are extinct and only one extant taxon.

*Cg* might potentially be higher among extinct taxa as a result of several factors: secondary mineral deposition/precipitation in porous bone during fossilization; difficulty in measuring *Cg* in cases where the rock matrix is hard to distinguish from bone; repair to damage in fossil specimens; the specific choices of extinct taxa included; or other reasons. Whatever the causes, the effect is very strong and clearly presents a potentially confounding factor for both the bone ballast hypothesis and classification via pFDA.

Among the 59 specimens in *F0D2*, many more represent extinct (43 or 72.9%) than extant species (16 or 27.1%). We evaluated whether the observed imbalance could be due to random chance by first applying a permutation test of *Cg* rank, with the null hypothesis being no difference between the *Cg* values of extinct versus extant taxa (see Materials and methods). We calculated *P* values for the null hypothesis that the rank distributions of extinct and extant are the same (Table 4). We then performed a second, “coin-flip” test, using a binomial distribution to determine coin-flip *P* values for an alternative null hypothesis that the counts of extinct and extant specimens in each group resulted from random chance. These tests were performed on all four dataset variations (Table 1) for both *F0D0* and *F0D2* subsets of femur and rib data.

**Table 4:** Permutation and coin-flip tests of rib and femoral datasets.

	All	Extinct		Extant		Permutation test		Minority group	Rank		Coin-flip *P* value
Dataset	*n*	*n*	%	*n*	%	Trials (×10^6^)	*P*		1st	2nd	
**Femur *F0D2* from ds1**	59	43	72.9%	16	27.1%	16	0.0011	Extant	16	22	0.00019
**Femur *F0D2* from ds3**	51	36	70.6%	15	29.4%	16	0.00028	Extant	16	22	0.00142
**Femur *F0D0* from ds1**	59	5	8.5%	54	91.5%	16	0.556	Extinct	6	13	8.7 × 10^−12^
**Femur *F0D0* from ds3**	50	35	70.0%	15	30.0%	16	0.142	Extinct	6	13	1.7 × 10^−11^
**Rib *F0D2* from ds2**	49	25	51.0%	24	49.0%	16	0.385	Extant	2	4	0.112
**Rib *F0D2* from ds4**	34	15	44.1%	19	55.9%	16	0.813	Extinct	2	4	0.108
**Rib *F0D0* from ds2**	63	2	3.2%	61	96.8%	100	<10^−8^	Extinct	3	41	2.1 × 10^−16^
**Rib *F0D0* from ds4**	58	1	1.7%	57	98.3%	16	n.a.	Extinct	2	n.a.	2.1 × 10^−16^

The key datasets used for comparison purposes are summarized by the count of points belonging to extinct or extant taxa, along with their percentages. Each dataset contains a minority of either the extinct or extant taxa. The top two ranks of the minority group for each dataset are listed. See text for details of the permutation and coin-flip tests, which assess the probability that each dataset is a representative sample. Abbreviations: 1^st^ and 2^nd^, rank numbers of highest-ranking and second-highest-ranking specimens in the minority group, respectively; n.a., not applicable. Shading indicates *P* < 0.05.

The permutation tests on *F0D2* femoral data from ds1 and ds3 have *p* ≤ 0.0011; we therefore reject the null hypothesis that the distribution of *Cg* values is the same for extinct and extant taxa (Table 4, shaded). The test result for the *F0D0* rib data from ds2 similarly rejects the null hypothesis with high probability (Table 4, shaded). Overall, these statistical tests and *P* values demonstrate that the distributions from which *Cg* is drawn differ for extinct and extant taxa, at least for these datasets.

We find that the foundational assumption that *Cg* can be used as a marker for both groups is violated and that the resulting classifications are biased by differences in the distributions of *Cg*. In the ds1 (femoral) dataset, pFDA assesses the statistical affinity of test taxa with the “subaqueous foraging” class *F0D2* (shown in Table 3) and with the terrestrial *F0D0* class. *F0D2* is 72.9% extinct and 27.1% extant, but for *F0D0* the imbalance is reversed: 8.5% extinct and 91.5% extant. The large imbalance in extinct versus extant, coupled with the fact that the *Cg* distributions are not the same (*i*.*e*., the null hypothesis of the permutation test is rejected) makes classification using these datasets suspect.

The results of the coin-flip tests on all four *F0D0* datasets further indicates that it is extraordinarily unlikely that the pronounced imbalances between extinct and extant taxa in these datasets are the result of random chance (Table 4, shaded results). In the rib datasets ds2 and ds4, it appears plausible that the *F0D2* data were randomly selected from both extinct and extant taxa. That is also the only subset for which the permutation test does not reject the *Cg* distribution. However, pFDA compares this seemingly balanced set to the rib *F0D0* subset, which has an extreme extinct/extant imbalance (3.2% to 96.8%) and highly significant rejection of the null hypothesis that the *Cg* rank distribution is equal. Since both class datasets must be valid for the pFDA test to be valid, we find the results of this classification also to be suspect.

The results of our statistical analysis cast grave doubt on the validity of classifications made with the datasets employed by Fabbri *et al*. Whether the marked discordance between the distributions of *Cg* for extinct and extant taxa arises due to biological differences or measurement or selection bias is immaterial to its impact in undermining confidence in the classification of the spinosaurids. Our additional finding that the imbalance between extinct and extant taxa is strongly biased in opposite ways for the two classes raises further concerns about that classification result.

Any future pFDA study that mixes extinct and extant taxa, or any other distinct groups, should explicitly list assumptions about the distribution of variates across the groups and then test those assumptions to ensure that there is no possible bias in the result.

#### Ignored and redundant taxa

When constructing datasets for comparative analysis, investigators must inevitably make choices and handle pragmatic issues, such as the availability of specimens from the literature or from collections. Fabbri *et al*. incorporated 78 taxa in the ds2 rib dataset from Canoville *et al*. [46] but ignored an additional 43 extant species from that study that are potentially relevant from a comparative basis and would merit inclusion, including varanids that range from semiaquatic (*Varanus salvator*, the water monitor) to large-bodied terrestrial (*Varanus komodoensis*, the Komodo dragon). We found that 15 taxa that Fabbri *et al*. did use from that prior study have *MD*<2 mm, which makes them much less relevant for comparison to enormous spinosaurs.

In contrast, the Triassic aquatic reptile *Nothosaurus* and its close relatives (three genera) are overrepresented, accounting for ~15% of the femoral (*F0D2*) dataset and 21% of the extinct taxa (Table 3). *Nothosaurus* is represented by six specimens. We found that the value of *Cg* in *Nothosaurus* is significantly negatively correlated with *MD* (S1 Fig; *R*^2^ = 0.84). The bone density data for this taxon would thus not scale to the body size of a spinosaurid (because *Cg* would drop to near zero). Some studies have suggested that larger nothosaurs may have adapted to ecosystems and active swimming lifestyles; if so, that might be related to this phenomenon [135,136].

Whatever the cause, the strong negative allometry of *Cg* with *MD* suggests that this is yet another confounding factor complicating the use of *Cg*. We find that the clade should have been dropped from the training dataset; instead, they are overrepresented. Negative allometry of *Cg* with *MD* tends to bias the decision boundary downward. As the spinosaurids are near the top of the distribution of *MD*, this effect could bias their classification toward the *F0D2* class.

Fabbri *et al*. offered no rationale in their paper to justify the choices they made to include and exclude taxa. Future studies of this kind should set reasonable inclusion criteria based on sound biological and statistical reasoning and evidence, and then apply those standards objectively.

#### Variation in *Cg*

Fabbri *et al*. used measurements made on just two skeletal elements, and in most cases they represented an entire taxon by a single value of *Cg*. Their analysis failed to account for uncertainty in the *Cg* measurements but, perhaps even more problematically, tacitly assumed that it is valid to draw quantitative conclusions about a taxon from one measurement made on a single specimen. This subsection discusses results from our examination of the roles of uncertainty in *Cg* measurement and variation in bone compactness, both within and between specimens.

Prior research has now established that significant variation exists in *Cg* values unrelated to ecology or behavior. Biological factors that might affect *Cg* in dinosaurs as well as relevant extant taxa include: developmental variations among individuals of a species; the sex of the individual; changes in bone compactness that occur during normal ontogeny; variations in *Cg* among skeletal elements; and even variations among different locations along the shaft of a single bone [132,134,137–139]. Diagenetic and taphonomic factors—including fracturing, deformation, infilling, and external erosion—can also introduce variations in *Cg* measurements.

Measurement error is present in any biological parameter and may similarly accrue from several sources, such as the calculation of *Cg* from thin sections or CT scans, decisions taken in thresholding images, and the degree of repair of cracks and missing bone in damaged or incomplete specimens.

We searched the literature for systematic studies that have examined how *Cg* varies across the various possible sources of biological variation or measurement error. Finding none, we asked workers in the field of bone microanatomy if they were aware of studies that have quantified such variations. We were told that there have been no such systematic quantitative studies published to date.

Our search identified just one report that included more than a handful of *Cg* values for a single taxon. That paper focused on the manatee *Trichechus manatus latirostris* [140]. Domning and de Buffrénil measured *Cg* values in ribs from thin sections in 12 individuals that included males and females, as well as growth stages from 50 to 1057 kg. *Cg* values ranged from 0.8389 to 0.9962, with a mean of 0.9109 and a standard deviation of 0.0417, a relative range of 17.3% (relative range: high minus low values, divided by the mean and then multiplied by 100). Excluding the youngest (and smallest) three individuals reduces the size range (161 to 1057 kg) but has a trivial impact on the range of *Cg* variation (13.2%).

As a step toward a multitaxon study, we compiled multiple measurements of *Cg* from multiple individuals within or across studies for all taxa present in the datasets used by Fabbri *et al*. (Table 5 and references therein). We did the same for taxa in a recent study of flightless birds that included multiple individuals of the same species (Table 6) [110]. We find that multiple measurements of *Cg* in the same bone of the same species often exhibit relative ranges exceeding 10%. The median relative range among the entries in Tables 5 and 6 is 18.6%.

**Table 5:** Examples of individual variation in *Cg* measurements across sources used by Fabbri *et al*. [15].

Taxon	Bone	References	*MD* (mm)	*Cg*	*Cg* range
*Phoca vitrulina*	Rib	[43]	7.8	0.436	22.0%
		[46]	11.49	0.544	
*Sphenicus humboldti*	Rib	[15]	4.08	0.908	24.3%
		[46]	4.98	0.711	
*Giraffa camelopardalis*	Rib	[46]	21.4	0.544	1.6%
			18.16	0.553	
*Metriorhynchus*	Femur	[52]	27.38	0.828	46.1%
		[141]	n.a.	0.518	
*Ceratherium simum*	Femur	[52]	78.6	0.669	20.5%
			92.6	0.819	
			70.2	0.827	
*Mammuthus*	Femur	[52]	102.9	0.846	14.9%
			139.5	0.898	
			172	0.773	
*Nothosaurus mirabilis*	Femur	[142]	16.09	0.828	6.5%
			21.7	0.776	
*Nothosaurus*	Femur	[143]	5.168	0.955	4.9%
			8.125	0.938	
			5.464	0.909	
*Simosaurus*	Femur	[144]	22.97	0.865	12.4%
			22.9	0.764	
*Diceros bicornis*	Humerus	[52]	79.6	0.866	7.9%
			70.2	0.937	
*Ceratherium simum*	Humerus	[52]	90.7	0.771	12.1%
			89.3	0.87	
*Dicerorhinus sumatrensis*	Humerus	[52]	50.6	0.941	17.5%
			52.8	0.7895	
*Scutellosaurus lawleri*	Humerus	[52]	6.1	0.767	18.6%
			6.2	0.748	
			139.5	0.898	

Data compiled for taxa in Fabbri *et al*. that have multiple *Cg* and *MD* measurements from the same bone. *Cg* variation (largest to smallest) ranges from 1.6–46.1% relative to respective means. (*Cg* relative range = (max − min) / mean × 100). Most are based on only two or three specimens. Abbreviations: *MD*, maximum bone diameter; *Cg*, global bone compactness; max, maximum; min, minimum; n.a., not available.

**Table 6:** Examples of individual variation in *Cg* measurements of flightless birds from Canoville *et al*. [110].

Taxon	Bone	*MD* (mm)	*Cg*	*Cg* range
*Dromaius novaehollandae*	Tarsometatarsus	32.7	0.655	15.6%
		28.5	0.560	
*Rhea americana*	Femur	n.a.	0.656	47.5%
		22.6	0.404	
	Tibia	17	0.459	33.5%
		21.6	0.644	
	Tarsometatarsus	20.3	0.826	28.2%
		16.5	0.618	
*Struthio camelus*	Femur	46.3	0.392	30.2%
		55.1	0.289	
*Aepyornithidae*	Femur	96.5	0.512	28.1%
		82.4	0.386	

Variation in bone diameter and compactness among multiple specimens of four flightless birds (not found in the datasets used by Fabbri *et al*. [15]). *Cg* ranges are relative to respective means. (*Cg* relative range = (max − min) / mean × 100). Abbreviation: *MD*, maximum bone diameter; *Cg*, global bone compactness; n.a., not available.

Median variation of 18.6% is a very large percentage, given the limited range of *Cg*. For example, the mean value of *Cg* in the ds1 *F0D0* subgroup is 0.610, whereas for ds1 *F0D2* it is 0.840—a relative range of 31.8%. The mean for ds2 *F0D0* is 0.653, and for ds2 *F0D2* it is 0.827—a relative range of 23.6%. To place this in context, the variation among individuals is more than half (58.6%) of the variation between the *F0D0* and *F0D2* groups for femora, and it is more than three-quarters (79.0%) of the intergroup variation for ribs.

As noted above, Fabbri *et al*. reported *R*^2^ coefficients from their PGLS regression on the taxa with *D* = 2 (“subaqueous foraging”) that indicate that *D* explains about 17.2% of the total variation in *Cg* for the femoral dataset and 10.8% for ribs. The 18.6% median value for *Cg* variation between individuals exceeds both of those *R*^2^ values. We thus find that the variation among individuals could be larger than the differences in *Cg* that Fabbri *et al*. found between subaqueous foragers versus not.

Most of the taxa in Tables 5 and 6 are represented by only two or three specimens. Better assessment of variation in *Cg* will require larger sample sizes for *Cg* among conspecifics and across a greater range of taxa.

It is possible that larger samples of individual variation and studies on more species would show the median variation found here to be atypical. But even a few taxa that exhibit large relative ranges—such as the maximum range in *Cg* observed in flightless birds (47.5% in femora of *Rhea americana*, Table 6)—could bias a discriminant analysis, whether LDA or pFDA. The available dataset of cases in Tables 5 and 6 is too small to accurately characterize variation, and at present we cannot determine the sources of variation. We do have enough information, however, to caution researchers about the degree to which individual variation could bias or invalidate statistical analyses.

Until the variation in *Cg* is better characterized, extra care must be used in any analysis that attempts to use *Cg* to classify taxa—whether by pFDA, LDA, or other statistical methods. Qualitative descriptions or broad observations of increased *Cg* in some taxa versus others may not be impacted, but statistical methods that hinge on the precise value of *Cg* are very much at risk of being affected.

### Variable *Cg* and infilling

**Fig 4:**
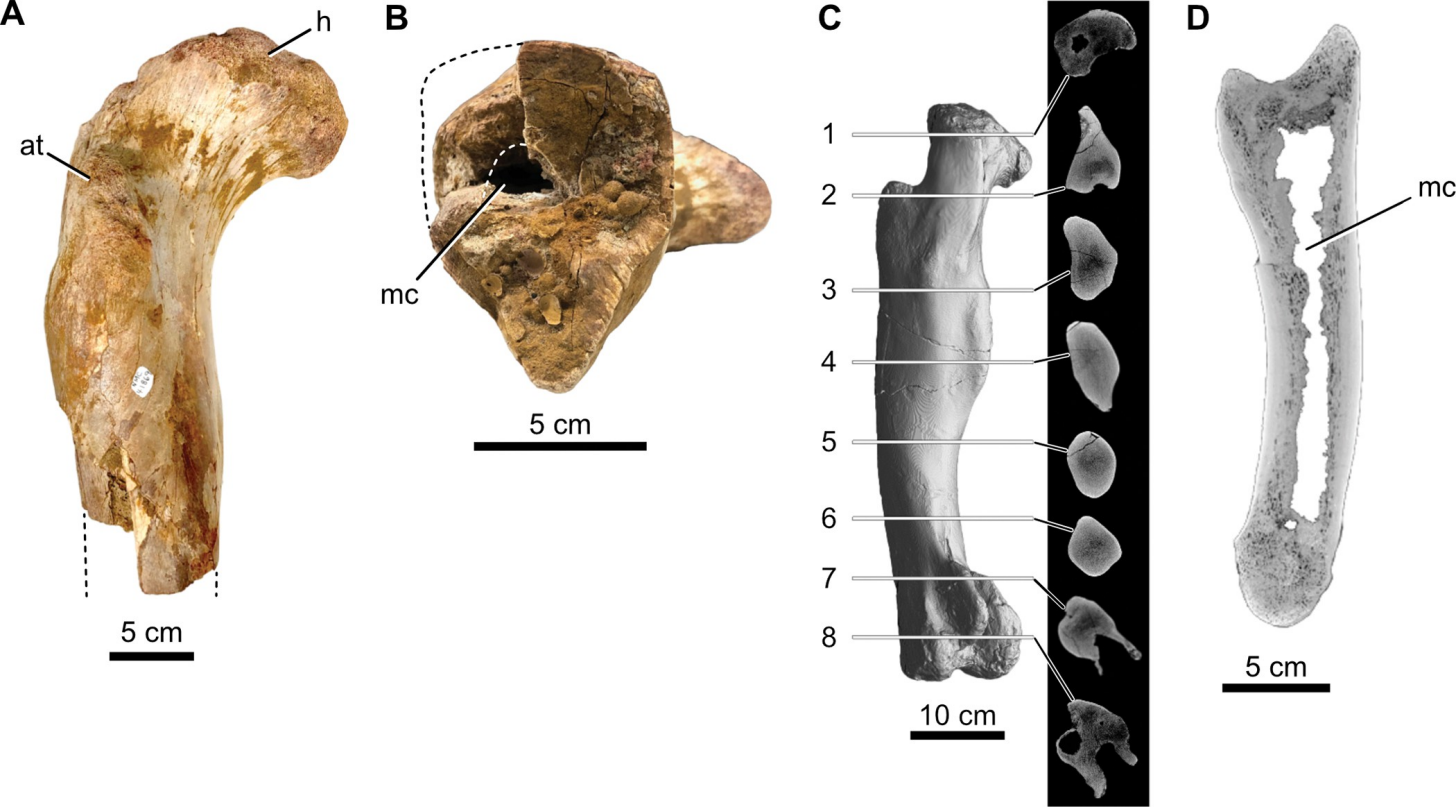
Femora and a manual phalanx of cf. *Spinosaurus aegyptiacus* from the Kem Kem Group in Morocco. (A, B) proximal one-half of an isolated right femur in anterior view and distal midshaft cross-sectional views (CMN 41869); (C) CT scan of the left femur of the neotype with eight cross sections (FSAC-KK 11888); (D) CT scan of an isolated right phalanx I-1 in sagittal cross section (UCRC PV8). Abbreviations: at, anterior trochanter; h, head; mc, medullary cavity. CMN 41869 images provided by Jordan Mallon.

Medullary cavities in long bones of the fore and hind limbs of *Spinosaurus* are variably infilled (Fig 4B and 4C). Fabbri *et al*. based their estimated *Cg* for *Spinosaurus* on one thin section taken from one fully infilled subadult femur (Fig 4D). A second femur of *Spinosaurus* [145] (Fig 4A and 4B) is slightly larger than the infilled neotypic femur (Fig 4C) but has a significant medullary cavity lined with cancellous bone that would register as significantly less dense in thin section at midshaft. Only two subadult femora are available for *Spinosaurus*—too few to generalize whether such variation is common or rare. In extant birds, intraspecific variation has also been recorded in the volume and location of medullary cavities [146]. These examples underscore the need to sample species more broadly rather than to accept a single measurement of bone compactness as representative of a given species.

**Fig 5:**
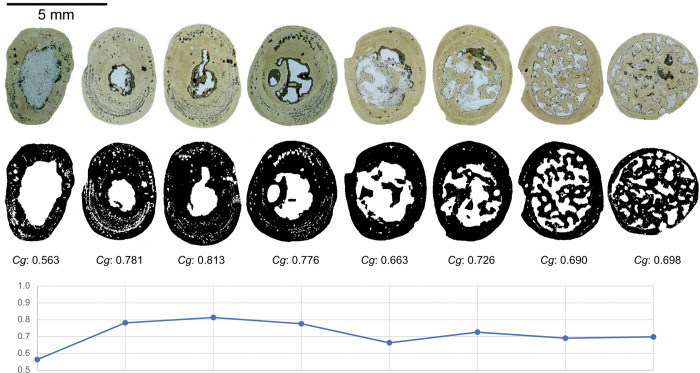
Variation of *Cg* at different points along a single *Nothosaurus* dorsal rib. Images from Klein *et al*. **[147: Fig 3, panels B1–B8]** were used to measure variation in *Cg* (~35%) along the rib shaft. Images supplied by Nicole Klein.

Some evidence shows that *Cg* can vary significantly with position along the shaft of long bones or ribs as bone diameter changes and cross sections encounter external trochanters or condylar ends, or for other reasons such as a variable medullary cavity. Klein *et al*. made a sequence of thin sections along the shaft of a dorsal rib of the marine reptile *Nothosaurus* [147] and found that *Cg* varied by ~35% within the rib (Fig 5). Although Fabbri *et al*. did not include this particular specimen in their study, nothosaurs make up a significant part of their dataset. *Cg* variation along the shaft of femora or ribs of other taxa has not been documented. While femoral sections are often taken at mid-shaft, no such standard exists for ribs.

This intraspecimen variation of 35% in a *Nothosaurus* rib is nearly double the median variation of 18.6% measured above between individuals. Though it is based on only a single rib specimen, which may not representative, we found in our analysis above that *Nothosaurus* taxa have variation in femoral *Cg* that is much smaller than the median (Table 5).

Several recent studies have examined bone microanatomy variation within a long bone by making 3-D scans of the entire bone. Previous studies have usually assumed that amniote long bones have a relatively uniform, usually tubular, structure along the diaphysis, in which case a sample taken mid-diaphysis would fairly represent the majority of the bone [41,148–150]. However when Nakajima *et al*. studied the humerus of 52 species of turtle, they found considerable variation in 3-D bone microanatomy along the diaphysis—suggestive that there could be corresponding variations in *Cg*—but unfortunately reported only mid-diaphyseal *Cg* values [137]. Similarly, Houssaye and Botton-Divet imaged the humerus and femur from eight species of otter, found considerable internal 3-D differences in bone microanatomy along the diaphysis, but reported only mid-diaphyseal *Cg* [134]. Amson scanned the humerus of specimens of 164 taxa of extant and extinct therian mammals and helpfully reported *Cg* values at multiple points along the proximodistal axis [138]. To facilitate comparison of bones of different lengths, the study rescaled positions along the axis to fall within the range 0 (proximal end) to 1 (distal end). *Cg* values were reported for the range from 0.3 (*i*.*e*., a distance 30% of the length of the bone from the proximal end) to 0.7. Amson found that in many taxa, but not all, *Cg* is not quasi-constant along a tubular structure but instead tends to increase from the proximal to distal portions of long bones, suggesting a linear gradient of bone infilling. The slope of the gradient differs for aerial, aquatic, subterranean, and terrestrial mammals, suggesting that bone microanatomy details across a bone may have greater potential for lifestyle inference than a single point measure of *Cg* does. Amson reported the mean *Cg* values at the 0.3 and 0.7 scaled distance from the proximal end the humerus for each lifestyle group [138: Table 1].

From these average values, we calculated the variation in *Cg* (*i*.*e*., (max−min)/mean) between those two points in the same bone: aerial is 17.9%, aquatic is 15.6%, subterranean is 32.1%, and terrestrial 32.8%. With few exceptions, *Cg* varied considerably across different points of the same humeral specimen [138: Fig 2]. These results support Amson’s conclusion that “there is a rather consistent increase in global compactness along the diaphysis of therian mammals.” This effect would explain variation in different positions along the same specimen. It also suggests that using the *Cg* measured at a single point may not capture the bone ballast effect of the bone very well. If there is a linear gradient in *Cg* with different slopes for each lifestyle, the integrated effect of *Cg* on the whole bone mass would be systematically biased.

However, this study was limited both to mammals and to the humerus. Although the results are suggestive, we cannot confidently extrapolate them to other groups or other skeletal elements. The variable infilling of long bones, as well as variation along different locations in the same skeletal element, still present sources of uncertainty. Until these effects are quantified, caution is required for any analysis that relies on precise values of *Cg*.

#### Attempted replication of *Cg* values reported for spinosaurids

Fabbri *et al*. reported new *Cg* values for spinosaurid taxa from measurements they made for their study [15]. We attempted to replicate those measurements by applying methodology from the literature (see Materials and methods). We also made a new measurement of *Cg* in a *Spinosaurus* specimen that was not included in ref. [15], but that they subsequently analyzed [16]. The results of our replication attempts provide useful examples of the extent to which variation in measurement contributes to the uncertainty of reported *Cg* values.

In addition to the biological, diagenetic, and taphonomic sources of variation described in the previous subsection, methodological differences in bone density determination can introduce variations in *Cg*. Relevant factors include the source type of bone section analyzed (CT digital scan, mounted thin section); the threshold value used to binarize a section image; and contour, masking, or repair steps taken prior to measurement of *Cg*. The many sources of variation increase the likelihood that independent researchers will obtain different quantitative values for *Cg* when deriving measurements from the same specimen or even the same cross section.

Fabbri *et al*. reported a very high *Cg* of 0.968 for *Spinosaurus*, a value that they calculated from a binarized image based on an image taken of a two-part thin section from the femoral shaft of the neotype skeleton [5]. That thin section, which was made by one of us (PCS), was taken on the narrow portion of the femoral shaft below the fourth trochanter (Fig 4C, section 5) and shows complete infilling of the medullary cavity. Fabbri *et al*. calculated a *Cg* value from a binarized image of this section that showed a small oval core of low density and an open (white) crack separating a portion of the cortex [15: Fig 1B,16: Fig 1A].

**Fig 6:**
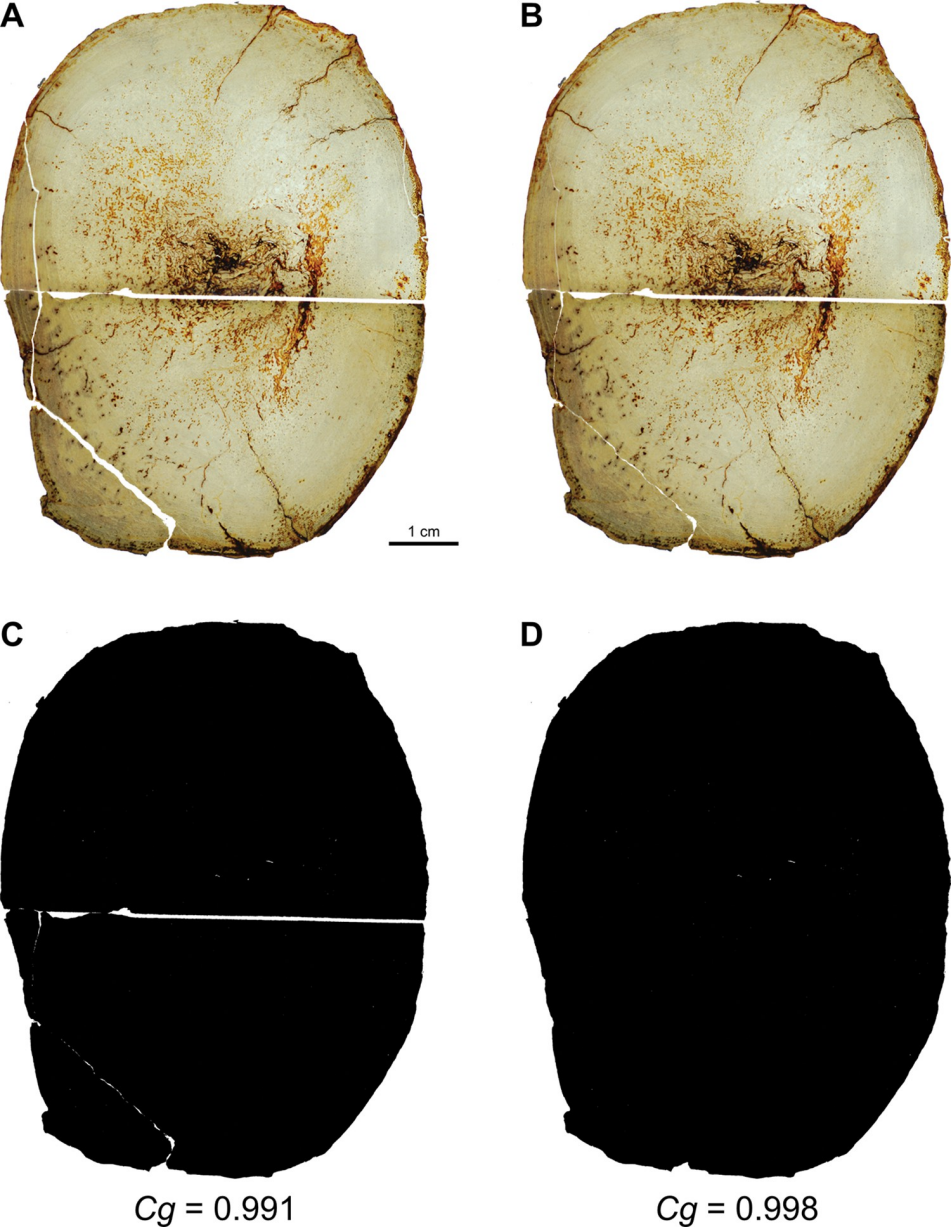
Reevaluation of *Cg* in a two-part thin section from the left femur of the neotype specimen of *Spinosaurus aegyptiacus* (FSAC-KK 11888). (A) Transmitted light image of a two-part thin slice from the mid shaft. (B) Thin slice image modified to close gaps created by natural breaks. (C) Binary image and associated *Cg* value without filling the cracks and the gap between section halves. (D) Binary image and associated *Cg* after filling the cracks and the gap between section halves.

Inspection of the thin section under magnification reveals several details that are otherwise impossible to discern from whole or half thin-section images. First, there is no medullary cavity, despite a dark-stained region in the center of the bone shaft (Fig 6). The central core is entirely filled in with bone that is slightly more cancellous. Second, a vertically oriented dark-red zone to the right of the core, which shows up as a less-dense zone in the binarized image published by Fabbri *et al*., is an artifact of hematitic stain. We found no difference in the bone texture or density of this zone when we viewed it under magnification. Third, a crack separating a portion of the lower-left thin section occurred during production and mounting of the thin section. The gap created by the crack should be closed digitally prior to *Cg* measurement. The section was also cut into two pieces, creating the horizontal gap, which should also be filled before analysis.

We found that accounting for these factors slightly elevates the *Cg* of the neotype femoral section to 0.998, our best estimate. This section shows an essentially fully infilled condition, whereas the binarized image reported by Fabbri *et al*. shows what appears to be an ovoid, less-dense core that results in their *Cg* estimate being approximately 3% lower than ours.

In response to our critique of Fabbri *et al*. [64], they incorrectly cited our response and introduced misinformation [16]:

Additionally, based on CT scan imaging, Myhrvold *et al*.^1^ accuse us of ignoring a medullary cavity in the femur of the neotypic specimen of *Spinosaurus* and that we are incorrectly oversampling bone tissue based on a thin section of the femur. As shown in Fig 1, cross sections obtained from the CT scan presented by Myhrvold *et al*.^1^ lack adequate contrast and resolution, obscuring any details of its internal structure, contrary to the thin section used in our study.

We have not suggested at any point that there is a medullary cavity in the neotypic femur (FSAC-KK 11888), neither when the thin section was first published [5] nor as later discussed in our critique [64]. The femoral CT scan of FSAC-KK 11888 figured here (Fig 4C, section 5) shows a slightly lower density toward the core but no medullary cavity.

**Fig 7:**
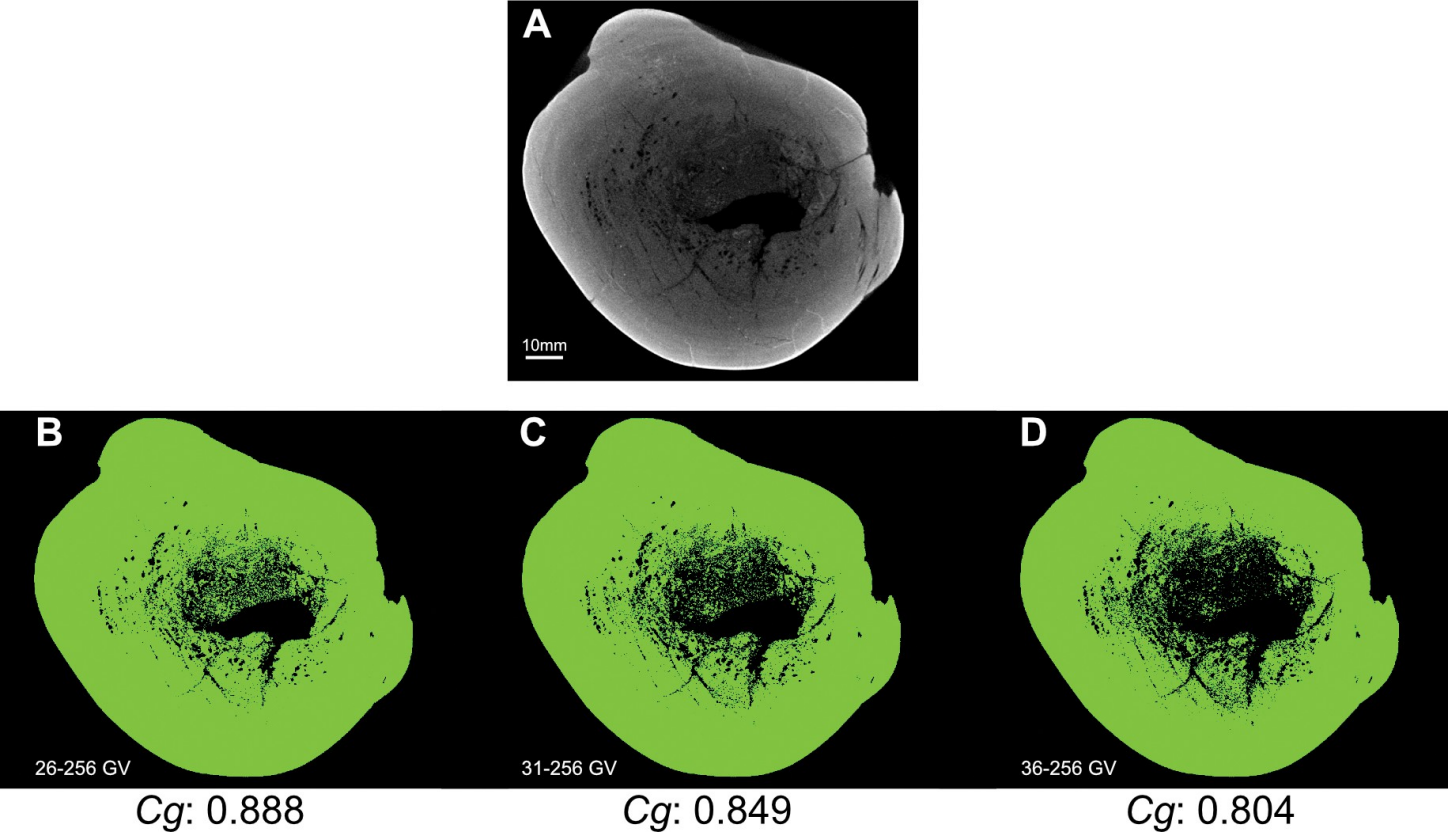
Impact of threshold choice on *Cg* in a cross section of the femoral shaft in a second subadult specimen of cf. *Spinosaurus aegyptiacus* (CMN 41869). (A) CT section from the proximal end of the shaft. (B–D) Section images and corresponding *Cg* values after processing with gray-value (GV) lower thresholds ranging from 26 to 36 GV on a 256 GV gradient. Threshold values determine which pixels are regarded as bone versus nonbone; higher thresholds yield lower *Cg* values.

The more salient finding is that infilling of the medullary cavity of the femur in *Spinosaurus* is variable, as shown by a second specimen (CMN 41869) of similar body size from the same beds in Morocco [64]. A persistent reduced medullary cavity is exposed by fracturing of the shaft (Fig 4A and 4B) and has been visualized with a CT scan proximal to the break (Fig 7). The absence of matrix infilling of cracks or external erosion obviates the need for digital repair prior to measurement of bone compactness. To calculate *Cg*, we used Mimics to threshold and transform the 8-bit grayscale pixels (values from 1 to 256) in the original CT image (Fig 7A) to binary (value 0 or 1). We explored the choice of threshold value as a factor by generating *Cg* values from three images made using thresholding with lower-end gray values of 26, 31, and 36, which yielded *Cg* values of 0.888, 0.849, and 0.804 respectively, a relative range of 9.9% (Fig 7B–7D). As anticipated, the *Cg* values from CMN 41869 are significantly lower than the values of 0.998 and 0.968 that we and Fabbri *et al*. measured for the nearly solid neotypic femur (FSAC-KK 11888).

We selected the middle image (Fig 7C) as the best binary visualization because it registers the less-dense cancellous bone near the medullary cavity without also obliterating what appear to be vascular canals in adjacent cortex on the left and lower sides of the medullary cavity. Our *Cg* result of 0.849 is very close to the mean (0.847) of the three values obtained from our thresholding range. In this case, there is no physical thin section to examine under magnification in polarized light to verify what is bone or mineralized infill.

Although this CT-based femoral section (Fig 7A) was not available to Fabbri *et al*. for their initial analysis [15], they later reported its *Cg* as 0.941 [16] but did not present the binarized image that was used for *Cg* measurement. We are unable to replicate that value, even approximately. Apparently they employed more extreme thresholding than the maximum we considered reasonable (Fig 7B). Extreme thresholding would raise *Cg* by obliterating some of the smaller spaces in the binarized section. In this case, our *Cg* measurements on the same section differed within a relative range of 9.9%, due to subjective procedures used in preparation of binarized images, whereas the relative range between our measured *Cg* values and the value reported in [16] is 15.7%.

Nonetheless, it is clear from available specimens of *Spinosaurus aegyptiacus* that some individuals nearing maturity maintained a reduced medullary cavity with a femoral-shaft *Cg* under 0.900. We reported accurately on this variable condition of medullary cavities in the long bones and their presence in certain vertebral centra in *Spinosaurus* [64]:

A second femur of *Spinosaurus*^2^ (Fig 1A and 1B), which is nearly identical in size to the infilled neotypic femur^3^ in their study (Fig 1C), has a significant medullary cavity lined with cancellous bone that would register as significantly less dense as a thin section at mid shaft. Medullary cavities are also variably present in forelimb bones of *Spinosaurus* (Fig 1D) resembling those in the long bones of *Suchomimus*, a fully “terrestrial” spinosaurid by their account. Fabbri *et al*.^1:ED,^ Fig 10 state that *Spinosaurus* and *Baryonyx* “possess dense, compact bone throughout the postcranial skeleton,” yet all three have pneumatic spaces in their cervical column^4^ that exceed in volume the variable long bone infilling, as well as large medullary cavities hollowing the centra at the base of the tail. Neither of these features are present in any secondarily aquatic vertebrate divers that employ bone density as ballast.

Commenting on this new information on variability, Fabbri *et al*. introduced several errors [16]:

Myhrvold *et al*.^1^ state that a single phalanx of the neotype of *Spinosaurus* possess a medullary cavity, invalidating our inference of widespread osteosclerosis across the postcranium of this animal; we show here that a cross section of the phalanx lacks any medullary cavity, as previously described in Ibrahim *et al*.^13–14^

and later:

Caudal vertebrae 1 and 4 of the neotype of *Spinosaurus*: contrary to what suggested by Myhrvold *et al*.^1^, no pneumatization is present in the caudal region of this taxon.

We clearly described the *variable* presence of the medullary cavity in both fore and hind limb long bones in *Spinosaurus*, figuring the medullary cavity along the length of a manual phalanx from an adult individual as opposed to the subadult neotype (Fig 4D). We were aware of the infilled manual phalanges of the neotype. The image they republished of this infilled shaft condition was taken by one of us (PCS in [5]) from a break in the proximal shaft of a proximal manual phalanx, not at midshaft as they indicated [16: Fig 1C]. Medullary cavities are variably present in CT scans of a broader sampling of manual phalanges referable to *Spinosaurus aegyptiacus* from the Kem Kem Group. The centra of anterior caudal vertebrae in *Spinosaurus* and other spinosaurids likewise have a capacious medullary space that hollows the interior of the centrum, as we reported [14]. Contrary to Fabbri *et al*. [16], no one has claimed that the hollowed anterior caudal centra in various spinosaurids are pneumatic.

**Fig 8:**
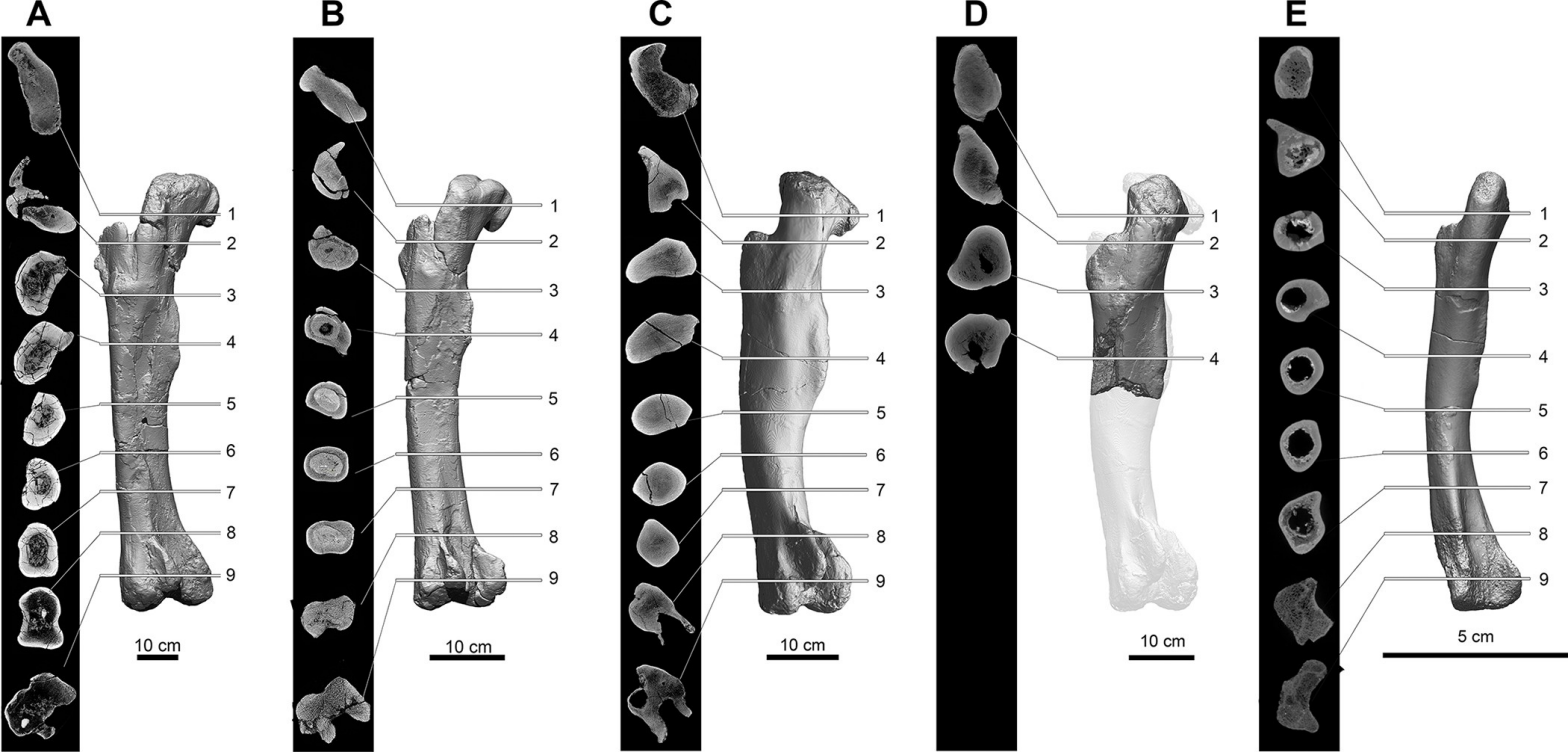
Transverse CT cross sections of the femoral shaft in two spinosaurids adjusted to the same side (left) and length. (A) *Suchomimus tenerensis*, adult (holotype), length 107.5 cm (MNBH GAD500). (B) *Suchomimus tenerensis*, juvenile, length 54.6 cm (MNBH GAD72, reversed). (C) *Spinosaurus aegyptiacus*, subadult (holotype), length 61.0 cm (FSAC KK-11888). (D) *Spinosaurus aegyptiacus*, subadult, estimated length 61.0 cm (CMN 41869, reversed), (E) *Spinosaurus aegyptiacus*, juvenile, length 11.8 cm (CMN 50382, reversed).

**Fig 9:**
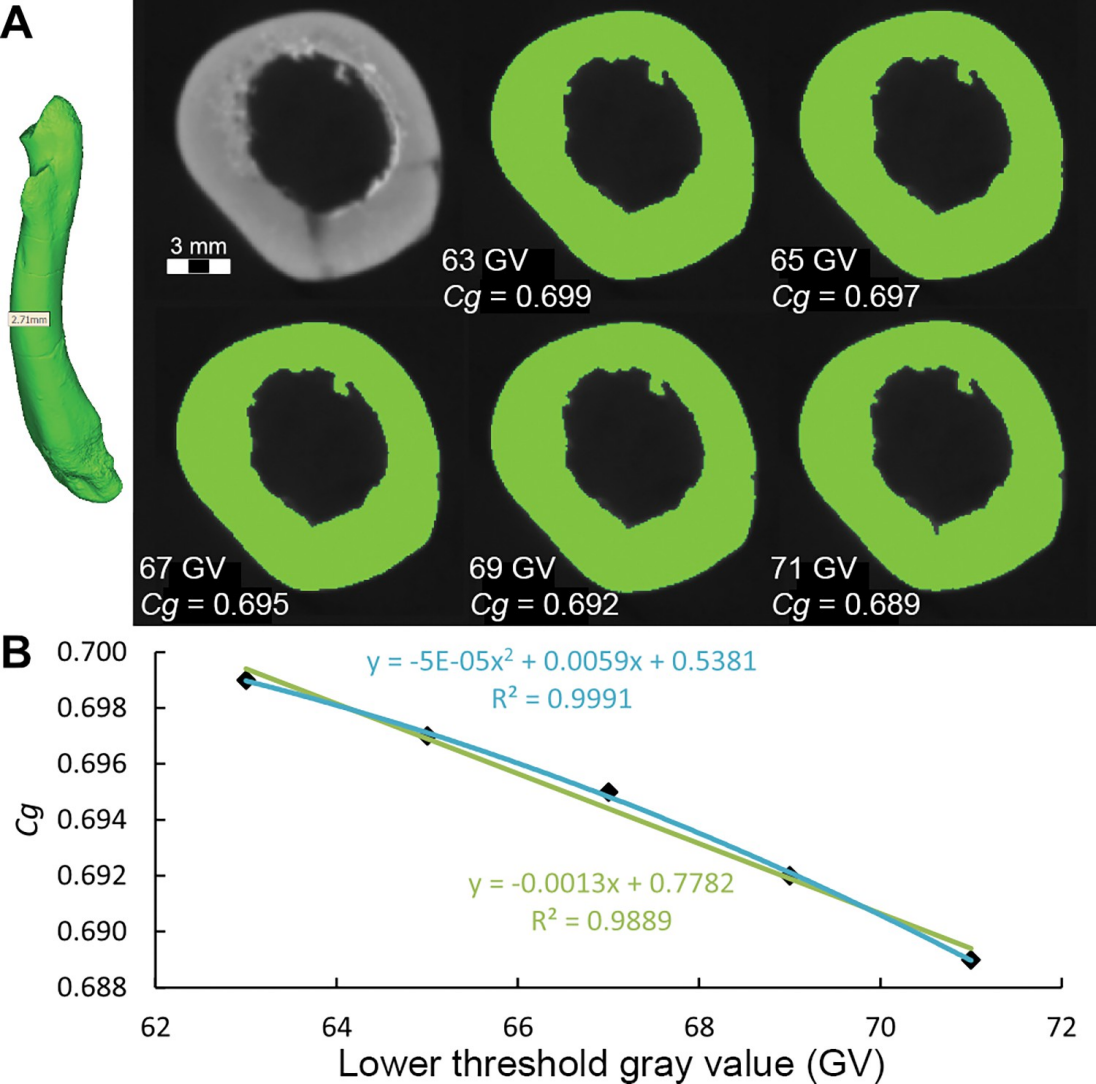
Bone compactness derived from CT scan of a juvenile femur of cf. *Spinosaurus aegyptiacus* (CMN 50382). (A) 3-D rendering and midshaft cross section generated from a CT scan, with binarized images (green) differing in their lower threshold gray-value setting. (B) Plot showing linear change of about 10% in *Cg* over the threshold range.

We examined CT cross sections from a third femur of *Spinosaurus aegyptiacus* from a very young individual, CMN 50382. The bone was collected in the same beds in Morocco as the first two specimens (Fig 8E). This femur, which measures only 11.8 cm in length [145], has a large medullary cavity extending along the length of its shaft and would indicate that the individual had a body length of approximately ~2.0 m. Ontogenetic infilling of the medullary cavity does not appear to have been initiated, with a midshaft *Cg* of approximately 0.695 (Fig 9).

The cross sections in Figs 8 and 9 show that there is considerable variation in the morphology of the femora. While Fabbri *et al*. focused their analysis on a single *Cg* measurement from *Spinosaurus*, the biomechanical parameter relevant to the bone ballast hypothesis is whole bone density. In some taxa, it may be necessary to use a bone microanatomy metric for the whole bone, or from multiple sections, but to our knowledge these are not available in the literature and would need to be developed.

In the case of *Baryonyx walkeri*, only the distal one-third of the right femur of the holotype is preserved [4]. There is crushing inward of anterior and posterior intercondylar areas, leaving only a small section of the shaft available for estimating bone compactness. This portion of the shaft was CT-scanned. Fabbri *et al*. used three closely spaced cross sections across ~6 cm of the shaft to generate three estimates of *Cg* ranging from 0.826 to 0.876 (relative range of 5.9%). The two most complete sections generated the minimum and maximum *Cg* values [15: S3E and S3F Fig]. For the section generating the maximum value, the cracks had been infilled with solid bone and used for *Baryonyx* in their femoral datasets [15: Fig 1B].

**Fig 10:**
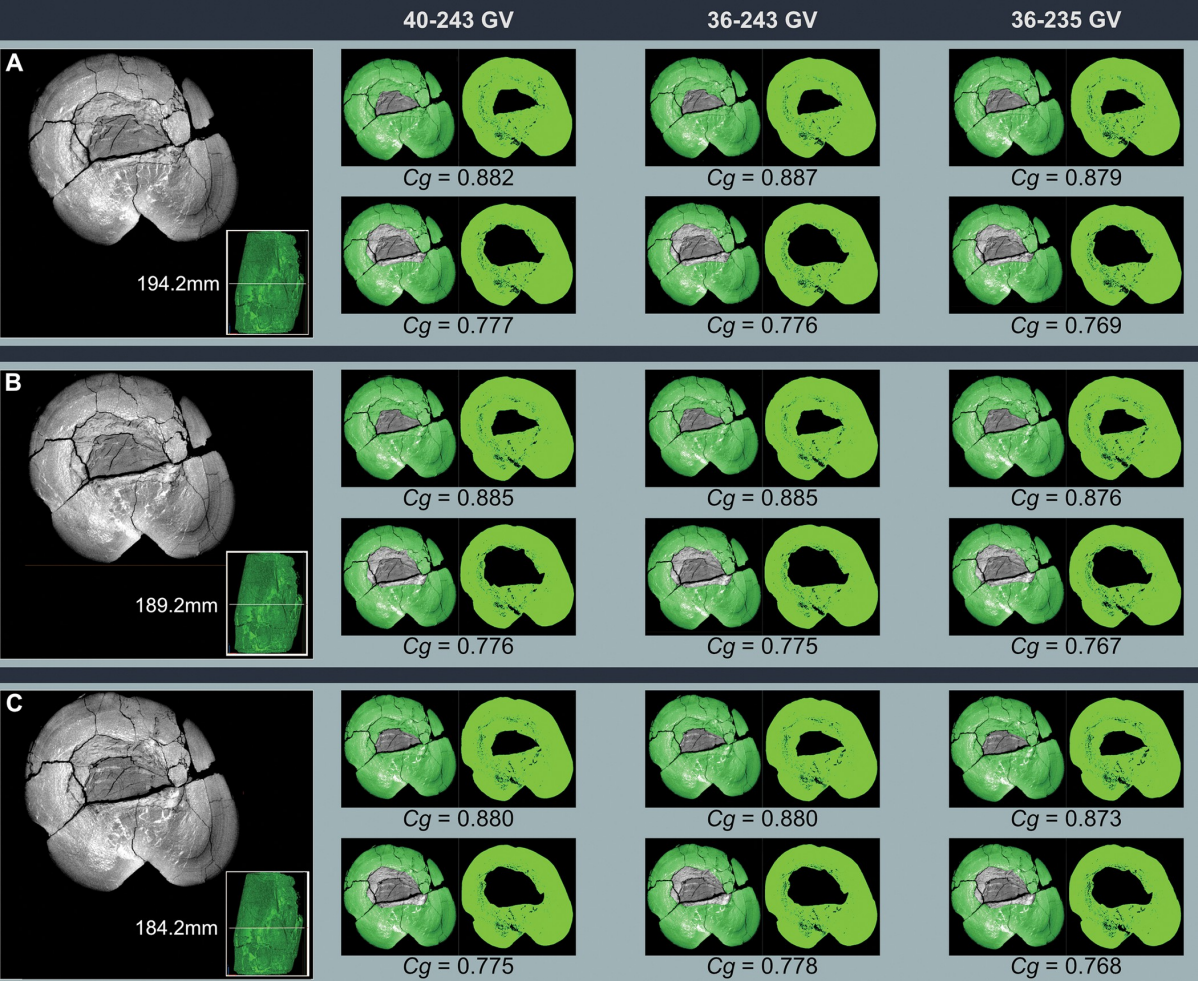
*Cg* measured in three adjacent CT cross sections through the distal shaft of the right femur of the spinosaurid *Baryonyx walkeri* (NHMUK 9951). CT sections (A–C, posterior aspect of femur oriented toward bottom) were taken in successively in more distal positions across 1 cm of the distal shaft of the femur in the portion of the shaft used by Fabbri *et al*. [15: S3E Fig] for their best estimate of *Cg*. The small inset view shows distal end of the femur in medial view with mm distance from the bottom of the radiograph provided by Fabbri *et al*. To the right are three gray-value (GV) thresholds (left to right: 40–243, 36–243, 36–235) capturing a reasonable range of values that might be selected by researchers to binarize the radiograph. For each threshold, masking of the matrix infilling of the medullary space is shown in transparent (left) and binarized (right) views. Option 1 (top row) attempts to replicate medullary masking as published by Fabbri *et al*. Option 2 (bottom row) eliminates additional medullary material that we confirmed from the CT scan as matrix infill rather than cancellous medullary bone. Fabbri *et al*. reported a *Cg* of 0.876. The *Cg* range for our three slices in the vicinity of their preferred CT section using their masking is 0.873–0.887 (mean 0.880); their *Cg* measure is near the low end of that range. The *Cg* range with our masking is 0.767–0.778 (mean 0.773), considerably lower than their *Cg* measure.

We attempted to replicate the *Cg* estimate of 0.876 that Fabbri *et al*. reported for *Baryonyx*, using the scan of NHMUK 9951 available on Morphosource to create new sections near one of the CT scan sections they published [15: S3E Fig]. We prepared three CT sections across 1 cm of shaft (Fig 10) in the region of their preferred section. We also infilled the cracks with solid bone density. We prepared two options for removal of matrix from the medullary cavity, each binarized with three different threshold values. The first option attempted to replicate the exact shape of the medullary cavity they defined and removed (Fig 10A–10C, top row of each panel). As a second option, we examined the CT section and made an independent evaluation of the limits of fossilized bone, adjusting the medullary cavity boundary outward to include more material that did not show bone texture (Fig 10A–10C, bottom row of each panel). We see no positive evidence in the scan for cancellous or cortical bone in those areas; they look more like mineral infill. Both times we filled in the cracks, in an attempt to repair taphonomic damage to the specimen.

We replicated their medullary cavity masking and found that their reported measure of 0.876 is near the low end of the range of *Cg* we obtained for our three sections (0.873–0.887). However, when we chose our own (slightly larger) masking for the medullary cavity, the range of values obtained (0.767–0.778) excludes their higher estimate for *Baryonyx*. Our mean *Cg* value for the distal shaft of *Baryonyx* (0.773) remains higher than the value reported by Fabbri *et al*. for *Suchomimus* (0.682), but that value seems artificially low. What seems clear at this point is that *Baryonyx*, like *Suchomimus*, retained an average-sized medullary cavity for a large theropod, the distal shaft of which generates a *Cg* less than 0.800.

Fabbri *et al*. figured two magnified thin sections for *Suchomimus tenerensis* identified as “G51” and “G94,” which are field numbers for the holotype (MNBH GAD500) and a referred subadult individual (MNBH GAD70), respectively [15: S2D and S2E Fig]. Neither of these specimens were sectioned, however, and MBNH GAD70 does not preserve more than the proximal end of one femur. We do not believe these thin sections pertain to *Suchomimus*.

**Fig 11:**
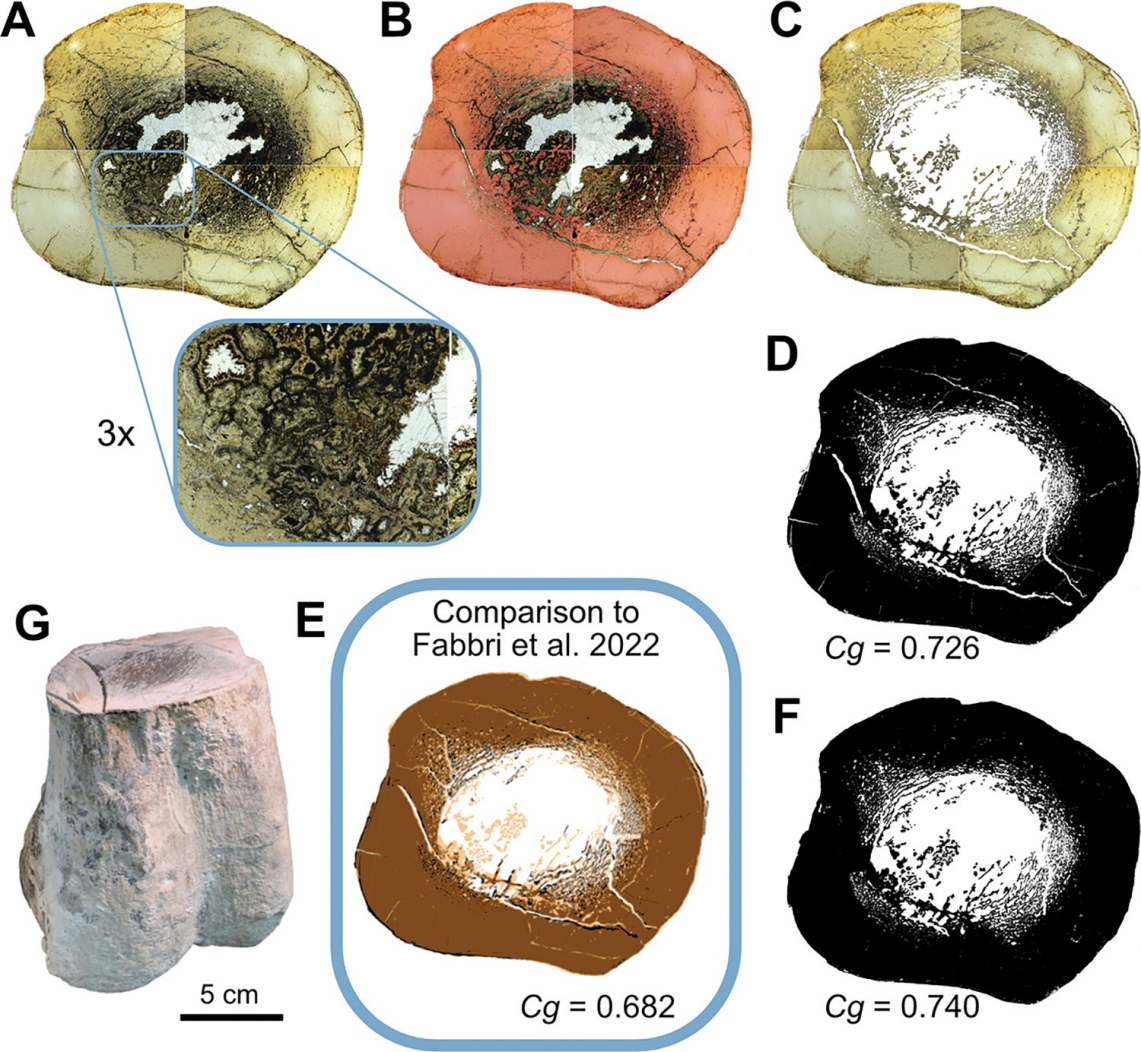
*Cg* derived from a thin section from the distal femoral shaft of an adult specimen of *Suchomimus tenerensis* (MNBH GAD99). (A) Composite image of the four-part thin section with an enlargement showing the complex relation between cancellous bone and dark-stained mineral infilling. (B) Cancellous bone (red) adjacent to dark-stained matrix in the core of the femoral shaft. (C) Digital removal of matrix adjacent to cancellous bone. (D) *Cg* from binarized image shown in (C). (E) Comparison of our binarized image (orange) to the *Cg* and binarized image published by Fabbri *et al*. (black) [15: S3D Fig]. (F) Final *Cg* after filling in matrix-filled cracks. (G) Distal femur showing position of thin section.

One of us (PCS) made a four-part thin section from the distal end of an adult femur of *Suchomimus tenerensis* (MNBH GAD99, Fig 11), which has a length (107.5 cm) and distal condylar width (23 cm) identical to that of the holotype. The position of the section on the distal shaft is similar to that taken in *Baryonyx*. An image of this thin section was refigured as a binarized image by Fabbri *et al*. [15: S3D Fig], who reported a *Cg* of 0.682. We commented, after reexamining the bone, original thin section, and high-resolution images of the section, that there was additional cancellous bone not shown in their binarized image that likely lowered their reported *Cg* value [64].

In their response to our preprint, they introduced misinformation without examining either the thin section or host bone [16]:

Myhrvold *et al*.^1^ suggest that we underestimated bone density in *Suchomimus* during the conversion of the femoral thin section into a black & white figure (the curating step prior to estimation of bone compactness), causing us to mis-identify bone as rock matrix. However, we did not apply our techniques blindly, but instead used careful observation to quantify bone compactness. As shown in Fig 1, the bone tissue in this specimen has a distinct white hue: Myhrvold *et al*.^1^ conflate the mineral infilling surrounding the trabecular bone and bone tissue.

Color variations prevent proper evaluation of many thin sections, including those examined here, without stereoscopic or at least magnified examination. Our examination of the *Suchomimus* section in the original high-resolution images found clear evidence of differentially distributed cancellous bone invading the medullary cavity, especially in the lower two thin-section quadrants (Fig 11A and inset), contrary to Fabbri *et al*. [16]. We differentiated cancellous bone from adjacent dark-stained mineral deposits under stereoscopic magnification of the thin section (Fig 11B). After digital removal of mineral deposits (Fig 11C) and binarization of the image (Fig 11D), a *Cg* of 0.726 was obtained, which is 6% greater than that reported by Fabbri *et al*. (Fig 11E). We made that measurement to be fully comparable with the estimate reported by Fabbri *et al*. without repair of matrix-filled cracks, which also effectively lower *Cg*. We then repaired the cracks to measure our final best estimate of the *Cg* of this specimen of *Suchomimus*, which is 0.740 (Fig 11F), approximately 8% higher than reported by Fabbri *et al*. and only 4% less than our best estimate of *Cg* in *Baryonyx*. Distal femoral shaft sections in *Suchomimus* (Fig 11G) appear to have *Cg* greater than 0.700.

We also took a thin section from the midshaft of a femur from a juvenile *Suchomimus tenerensis* (MNBH GAD72) with femur length approximately half that of the adult. Our examination of that section finds a relatively large medullary cavity (Fig 8B, S2 and S3 Figs). *Cg* in the juvenile (MNBH GAD72) was found to be 0.689 to 0.699 (high and low thresholds).

Our replication experiments produced several notable results. We found that, depending on the specimen, subjective effects arising from the threshold selected to binarize the image, from the removal of matrix, and from repair of cracks and erosion can contribute sizable measurements variations in estimates of *Cg*, up to a relative range of nearly 10% in one specimen. Variations were lower for other specimens, and the nearly solid *Spinosaurus* neotype showed very little variation. Future studies should be mindful of subjective factors such as these because they introduce variation in *Cg* that can confound quantitative analyses that rely on precise *Cg* values.

### Issues with the application of pFDA

The sections above have analyzed the theoretical and statistical justification for using *Cg*, as well as some problematic issues that arise with its use by Fabbri *et al*. In this subsection we present results from our examination of a variety of aspects to the statistical calculations involved in pFDA that directly affect the quality and validity of results.

#### Effects of training-set sample size on classification

A tacit assumption made by Fabbri *et al*. in their analysis, common to most statistical analyses in biology, is that underlying biological factors produce a true statistical distribution of the variates, and that the dataset classes reflect that distribution. The pFDA method, in particular, assumes that the true distribution for each class conforms to a multivariate normal distribution, or a close approximation thereof. But the parameters of those true distributions are *unknown*. The pFDA method must estimate the parameters from the finite sample in the training dataset. Although this situation is common to virtually all statistical analyses, it strangely seems to have been overlooked in the literature on pFDA, as well as in most biological applications of LDA, aside from a few exceptional examples [151].

Sample size, the number of distinct data points in the training dataset, is a key element determining the statistical power and precision of a pFDA analysis because it controls how well the finite sample approximates the underlying biological distribution. Neither Fabbri *et al*. nor any other pFDA study of which we are aware has offered any analysis of how the size of the training dataset affects classification accuracy.

The accuracy of binary classification has been long studied, and many mathematical metrics have been developed to measure it, including the metrics known as accuracy *A*, balanced accuracy *B*, the Matthews correlation coefficient *MCC*, and the true-positive and false-positive rates, all of which are defined and discussed in S1 Appendix, section 4.

**Fig 12:**
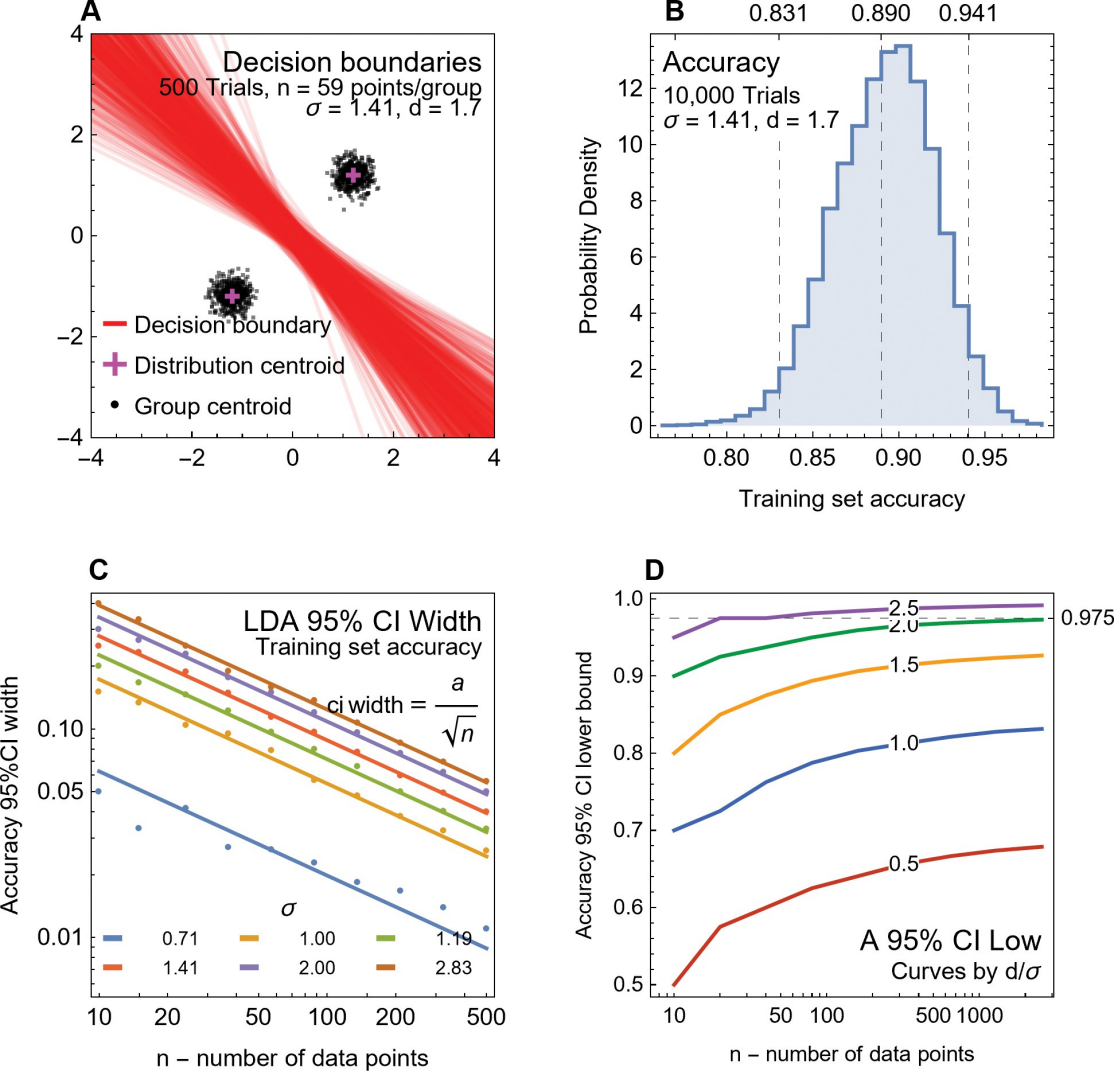
Sample-size effects in LDA/FDA. (A) Decision boundaries and centroids of point groups for 500 trials of 59 points drawn from a multivariate normal distribution of Eqs (1) and (2) with specified values of *σ* and *d*. The centroids of the distributions are shown by the magenta crosses; empirical centroids of each group of 59 points are black dots. The decision boundaries are red lines. Groups of 59 points are insufficient to accurately estimate the distribution centroid. The estimation error leads to variations in both the empirical-group centroids and the decision boundaries across Monte Carlo runs. (B) A histogram of the training-dataset classification accuracy is shown for 10,000 trials with the same parameters as (A). See Materials and methods for definitions of standard error *σ* and distance *d*. The theoretical accuracy for the values of *σ* and *d shown* is 0.885, but the 95% confidence interval extends from 0.831 to 0.941, a width of 12%. (C) Monte Carlo simulations of classification accuracy for point groups with *d* = 1.7 and varying values of *σ* and *n* points per group. Lines show an empirically derived relationship for the width of the 95% confidence interval in classification accuracy: CI width = *a*/*n*^1/2^, where is *a* is a fitting constant determined for each value of *σ*. (D) Monte Carlo simulations of 10,000 trials of LDA plot the lower bound *A*_LB_ of the 95% CI for accuracy *A*. Each curve has a different value of the ratio *d/σ*, from 0.5 to 2.5, and illustrates how the lower bound changes as a function of the number of points in each group, which range from 10 to 2500. Abbreviations: CI, confidence interval.

We performed Monte Carlo simulations of an LDA classifier to explore sample-size effects for the case of symmetric multivariate distributions of the form given by Eqs (1) and (2). The results, shown in Fig 12, display noticeable scatter among the empirical centroids derived from groups of 59 pseudorandom data points (chosen to approximately match the count in ds1) (Fig 12A). The empirical centroids only roughly approximate the true centroids of the distributions from which they were drawn. The decision boundaries separating these groups also show considerable scatter in both midpoint and slope. Assessing the classification accuracy of 10,000 trials of two groups of 59 points yields a histogram, which peaks at the theoretical classification accuracy of 0.915 and shows considerable scatter (Fig 12B), with a 95% confidence interval spanning 0.831 to 0.941 (12% of the accuracy).

Repeating this 10,000-run Monte Carlo experiment for multiple points per group 10≤*n*≤500 and for values of the standard-error parameter 0.707≤*σ*≤2.83, we find that the width of the 95% confidence interval on classification accuracy closely follows an empirically derived relation (Fig 12C). The general behavior is that the width of the confidence interval scales proportionately to 1/*n*^1/2^ for sample size *n*, as is typical for the normal distribution (and consistent with Eq (3)).

The lower bound of the confidence interval thus depends on both the ratio *d/σ* and the number of points in each group, as shown in Fig 12D. The dashed horizontal black line indicates the lower bound of the 95% CI *A*_LB_ = 0.975, which is the value of accuracy associated with *P*_rand_ = 0.05, a heuristic criterion for no more than 5% random error in classification (see Eq (7) of S1 Appendix, section 4). Eq (3) and the relationship between classification error and *A* (Materials and methods) can be inverted to show that *A* = 0.975 when *d/σ* = 1.96, which is the theoretical accuracy value in the infinite limit of group size, at which point the width of the 95% CI would be zero, so the estimate and lower bound are the same. If *d/σ*<1.96, then even an infinite number of points will not achieve *A*_LB_ = 0.975.

The curves in Fig 12D show that the number of points in each group used for LDA has a strong dependence on both the point count and *d/σ*. The curve representing *d/σ* = 2.0 (green curve in Fig 12D) asymptotically approaches *A*_LB_ = 0.975. The rightmost point plotted in this curve shows that for *n* = 2560 points per group, it has reached *A*_LB_ = 0.973. A greater ratio of *d/σ* = 2.5 dramatically changes the sample size necessary; *A*_LB_ = 0.975 for as few as 20 points per group (purple curve in Fig 12D).

Although these examples illustrate the threshold *A*_LB_ = 0.975, qualitatively similar behavior occurs for any *A*_LB_ threshold. Eqs (3) and (7) allows us to solve for the value of *d/σ* that reaches a selected threshold value of *A* in the limit of an infinite point count. For values of *d/σ* slightly above the value predicted by Eqs (3) and (7), we may need a large (but finite) number of datapoints. However, for values of *d/σ* that are larger than that value, a much smaller number of datapoints is needed.

This pattern is an example of the “ecological fallacy,” a common pitfall in statistical inference. Briefly stated, one generally cannot accurately classify a point by comparing it to its statistical distribution or to the average and variance derived from the distribution. In specific cases, the classification may succeed, but only if the variance of the distributions is sufficiently small. The ecological fallacy is discussed further in S1 Appendix, section 1. LDA, FDA, and pFDA are all generally subject to the ecological fallacy; the methods do not guarantee that the classifications they produce will be meaningful, no matter how large the datasets used. Only if *d*/*σ* is sufficiently large *and* the sample is sufficiently large will classification performance be adequate. These two factors interact to produce the behavior seen in Fig 12C.

These results pertain to classification accuracy of the training dataset, but a similar phenomenon occurs for any metric of classification performance. Classification accuracy is linear in the confusion-matrix components, whereas some metrics, such as *MCC*, are nonlinear in the components (S1 Appendix, section 4). The exact form of the relation between 95% CI width and the distribution parameters will thus change, but we expect the overall behavior to be qualitatively similar. In actual practice, we do not know the exact distribution and instead have only the finite sample to work with. Another complication is that pFDA, as used in Fabbri *et al*., has an additional source of random variation due to the creation of randomly generated phylogenetic trees.

We applied a bootstrap approach [27] to the datasets used by Fabbri *et al*. as a way to estimate the finite sample-size effects on pFDA. Implementation details are presented in Materials and methods.

**Fig 13:**
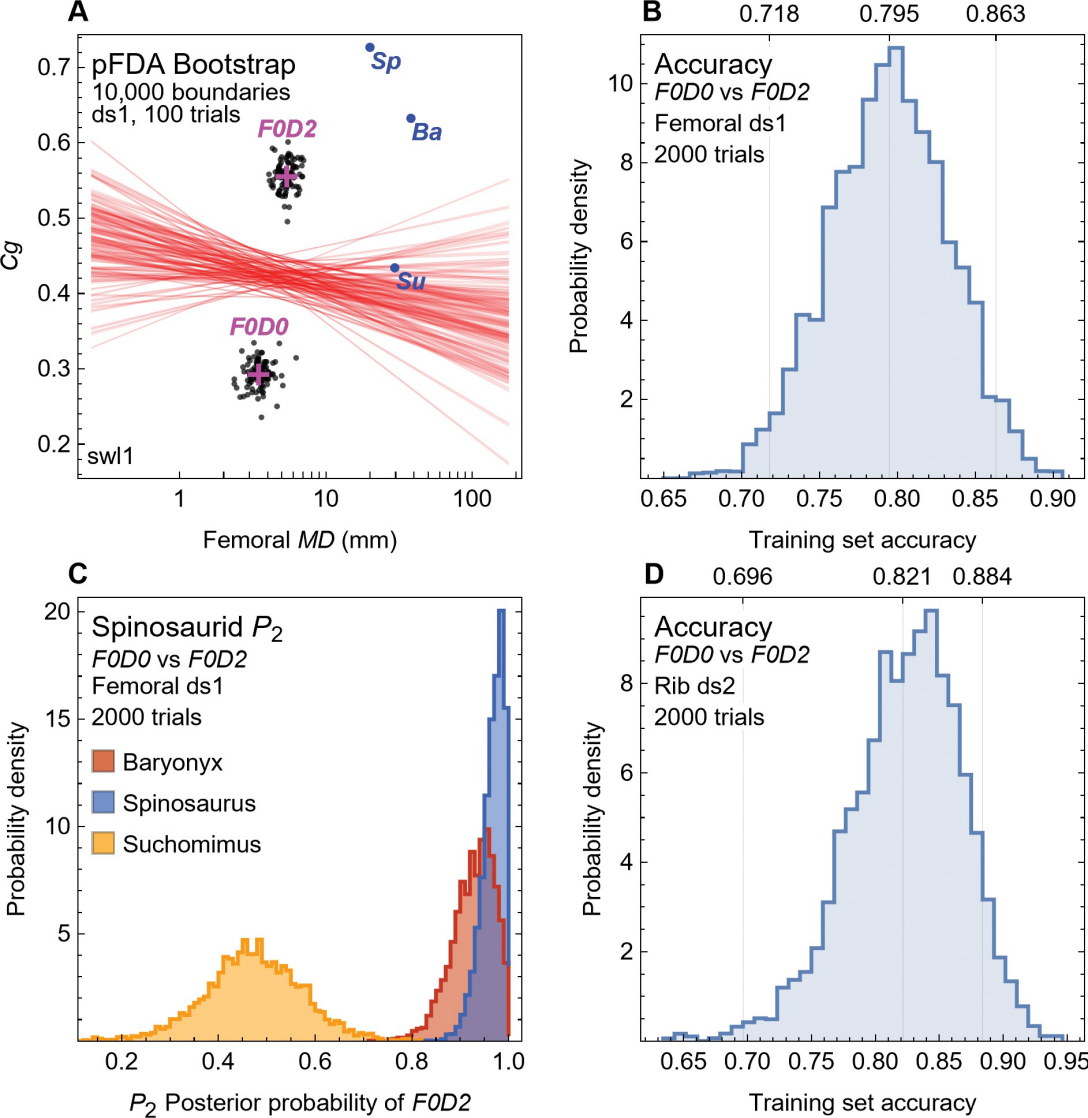
Sample-size effects for pFDA with *F0D0* and *F0D2* data from Fabbri *et al*. [15]. (A) Decision boundaries (red lines) and point-group centroids (black dots) for 100 trials created using a bootstrap method described in the text, operating on the *F0D0* and *F0D2* subsets of the Fabbri *et al*. femoral dataset ds1. Each bootstrap trial draws 100 trees at random, each with its own decision boundary. Even more than in Fig 12, considerable scatter is evident in both the centroid positions and decision boundaries. Data points for *Spinosaurus*, *Baryonyx*, and *Suchomimus* are plotted in blue. The downward slopes of most of the decision boundaries, as well as the leftward offset of the centroids of the *F0D0* subset versus that for *F0D2*, show the effect of lower *MD* for *F0D0*. (B) A histogram of classification accuracy of the training dataset is shown for 2000 trials of a parametric bootstrap. The median training-set classification accuracy is 0.795, and the 95% confidence interval is 0.718 to 0.863, a width of 0.163. (C) Histograms of *P*_2_, the posterior probability of belonging to group *D = 2*, for spinosaurid taxa across 2000 trials of the same dataset as (A) and (B). (D) Histogram of training-set classification accuracy similar to (B), but for rib data. The median training-set classification accuracy is 0.821, and the 95% confidence interval is 0.696 to 0.884, a width of 0.188. Abbreviations: *Sp*, *Spinosaurus*; *Ba*, *Baryonyx*; *Su*, *Suchomimus*; CI, confidence interval; BCa, bias-corrected-and-accelerated method.

Fig 13A presents the results of this process for 10,000 trials (100 trials, each replicated with 100 random trees). Each decision boundary has a corresponding classification of the points, yielding a confusion matrix, from which we then calculated the classification accuracy for the training dataset. Fig 13B plots the distribution of accuracy values as a histogram. The bootstrap samples also affect the posterior classification probabilities *P*_2_ (Fig 13C) for the *F0D2* group. *P*_2_ is the predicted probability that the taxon should be classified as a “subaqueous forager.” Our results from the bootstrap analysis are qualitatively unsurprising, in view of our results on the simplified synthetic dataset (Fig 12). The effect of small sample size leads to scatter in both the group centroids and the decision boundaries.

To estimate 95% confidence intervals, we performed 2000 bootstrap trials for each dataset and tabulated the training dataset errors. The bias-corrected-and-accelerated (BCa) method was used to assure good results on the confidence interval [27]. Because each case also has 100 random trees, 200,000 results were used for the creation of the confidence intervals (Table 7). The training-set error rate is widely considered to be overoptimistic, and our use of it is thus very conservative.

**Table 7:** Bootstrap-estimated 95% confidence intervals for training-set classification performance metrics.

Dataset	Metric	*F0D0* vs. *F0D2*			Many vs. *F0D2*		
		Median	95% CI		Median	95% CI	
**ds1**	*A*	0.795	0.718	0.863	0.836	0.771	0.894
	*P*_rand_	0.410	0.564	0.274	0.327	0.458	0.212
	*B*	0.794	0.717	0.863	0.841	0.781	0.899
	*MCC*	0.607	0.470	0.752	0.655	0.528	0.770
**ds2**	*A*	0.821	0.696	0.884	0.772	0.648	0.846
	*P*_rand_	0.357	0.607	0.232	0.457	0.704	0.309
	*B*	0.819	0.706	0.883	0.778	0.677	0.849
	*MCC*	0.637	0.415	0.767	0.521	0.332	0.670
**ds3**	*A*	0.833	0.725	0.892	0.895	0.839	0.950
	*P*_rand_	0.333	0.549	0.216	0.211	0.323	0.099
	*B*	0.833	0.725	0.892	0.897	0.840	0.945
	*MCC*	0.673	0.464	0.792	0.769	0.652	0.874
**ds4**	*A*	0.891	0.739	0.946	0.882	0.772	0.941
	*P*_rand_	0.217	0.522	0.109	0.235	0.456	0.118
	*B*	0.877	0.749	0.939	0.863	0.740	0.922
	*MCC*	0.765	0.467	0.883	0.700	0.472	0.841

To determine how well taxa in the training dataset are correctly classified by pFDA, we measured three classification performance metrics: *A*, *B*, and *MCC* (described in S1 Appendix, section 4), as well as the probability *P*_rand_ that the classification is completely random, which was calculated from *A* using Eq (4). For each of the four datasets used by Fabbri *et al*. [15], we performed 2000 bootstrap trials, each with 100 random trees, for a total of 200,000 data points. The BCa bootstrap method was used to construct accurate 95% confidence intervals for both the comparison classes used by Fabbri *et al*. [15] (columns “Many vs. *F0D2*”), which retain taxa with *F* = *0*,*1*,*2* and *D* = *0*,*1* as the alternative to the *F0D2* group, and also for the better-supported *F0D0* versus *F0D2* approach. The lower bound on the accuracy metrics formed by the minimum of the 95% CIs shows that the training-data classification with these datasets has typical errors of approximately 20% to 40%—higher in some cases. Abbreviations: CI, confidence interval; BCa, bias-corrected-and-accelerated method.

The primary effect of sample size is that the 95% confidence interval is much broader than the point estimates. The importance to the interpretation of pFDA classification results is that they are even more uncertain than one would expect from simply evaluating the training-set error from a single run. Consider dataset ds1: using the classification scheme of Fabbri *et al*. (Table 7 columns “Many vs. *F0D2*”), the median value of the accuracy metric *A* (Eq (6) in S1 Appendix, section 4) is 0.836 (83.6%), which is roughly consistent with their claim that the correct classification rate is “84–85% (femora)” [15]. However, the 95% CI for this value ranges from 77.1% to 89.4%. When we used the better-supported method of comparing the *F0D2* subset to just the *F0D0* group, we found that the median accuracy drops slightly to 79.5%, with the lower bound of the 95% CI falling to 71.8%.

Because half of the time the classifier performs worse than the median accuracy, a better way to characterize the minimum performance is to use the lower bound of the 95% CI, *i*.*e*., the minimum accuracy that allows us to be certain (to 95% confidence) that random effects will be no greater than 5%. Using the method of calculating the equivalent percentage of random trials *P*_rand_ (Materials and methods, Eq (4)), we find that an accuracy of 71.8% is equivalent to saying that the classification accuracy is correct 43.6% of the time, and completely random 56.4% of the time (*i*.*e*., *P*_rand_ = 0.564). That is certainly better than a random guess all of the time. However, a method that produced the correct result only about half the time and random results the other half would not normally be considered as sufficient scientific evidence to draw a valid conclusion—it would be too contaminated by random effects. Typically used statistical thresholds for random effects are 5%, an order of magnitude lower.

None of the 95% intervals in Table 7 would result in a value of *P*_rand_<0.05, which is the heuristic value that would correspond to about 5% random errors (see Assigning confidence to classifications in the Methods and materials section). Indeed, the highest median value found for *A* in the *F0D0* versus *F0D2* case is ds4; the lower bound on the 95% CI in that case is 0.877, corresponding to *P*_rand_ = 0.246, *i*.*e*., equivalent to a situation in which the classification is random 24.6% of the time. We must caution however that, as discussed in Materials and methods, *P*_rand_ is not equivalent to a formal *P* value. Instead, it is a heuristic that tells us that the training-set accuracy is equivalent to a case where the result is random with probability *P*_rand_ and correct the rest of the time.

The *P*_2_ metric of classification performance shown in Fig 13 is a metric of classification strength. Whereas *P*_rand_ measures overall classification performance, *P*_2_ is specific to an individual taxon. The bootstrap approach we used generates a distribution of *P*_2_ values (Fig 13C), along with its associated 95% confidence.

To assess the impact of variations in the data for the spinosaurid test taxa, we performed a sensitivity analysis, using hypothetical variants of the data points used by Fabbri *et al*. (Table 8 and S4 Fig). To be clear, we do not propose that these modified values are necessarily more correct or believable; the aim of the sensitivity analysis is to see how *P*_2_ for each spinosaurid taxon is affected by changes of various magnitudes to its test data point. Sensitivity analysis of this kind is a routine way to evaluate statistical classifiers.

**Table 8:** Alternative test-data points for spinosaurids.

Dataset	Taxon	*MD*	*Cg*	*MD* source	*Cg* source
**ds1** **(Femur)**	*Baryonyx 0*	154	0.876	ref. [15]	ref. [15]
	*Baryonyx 1*	154	0.887	ref. [15]	Fig 10, high
	*Baryonyx 2*	154	0.826	ref. [15]	Fig 10, median
	*Baryonyx 3*	154	0.767	ref. [15]	Fig 10, low
	*Spinosaurus 0*	81.52	0.968	ref. [15]	ref. [15]
	*Spinosaurus 1*	81.52	0.804	ref. [15]	Fig 7, low
	*Spinosaurus 2*	81.52	0.849	ref. [15]	Fig 7, med
	*Spinosaurus 3*	81.52	0.888	ref. [15]	Fig 7, high
	*Spinosaurus 4*	81.52	0.914	ref. [15]	ref. [16]
	*Spinosaurus 5*	133.434	0.968	Scaled by 1.64	ref. [15]
	*Spinosaurus 6*	133.434	0.804	Scaled by 1.64	Fig 7, low
	*Spinosaurus 7*	133.434	0.849	Scaled by 1.64	Fig 7, med
	*Spinosaurus 8*	133.434	0.888	Scaled by 1.64	Fig 7, high
	*Spinosaurus 9*	133.434	0.914	Scaled by 1.64	ref. [16]
	*Suchomimus 0*	120.6	0.682	ref. [15]	ref. [15]
	*Suchomimus 1*	120.6	0.628	ref. [15]	Scaled by 0.9
	*Suchomimus 2*	146.4	0.682	Actual max GAD500	ref. [15]
	*Suchomimus 3*	146.4	0.628	Actual min GAD500	Scaled by 0.9
**ds2** **(Rib)**	*Baryonyx 0*	42.2	0.921	ref. [15]	ref. [15]
	*Baryonyx 1*	42.2	0.8289	ref. [15]	Scaled by 0.9
	*Spinosaurus 0*	35.10	0.931	ref. [15]	ref. [15]
	*Spinosaurus 1*	35.10	0.838	ref. [15]	Scaled by 0.9
	*Spinosaurus 2*	57.45	0.931	Scaled by 1.64	ref. [15]
	*Spinosaurus 3*	57.45	0.838	Scaled by 1.64	Scaled by 0.9

Fabbri *et al*. [15] used one data point for each of the spinosaurid taxa *Baryonyx*, *Spinosaurus*, and *Suchomimus*, and these points are denoted by 0 after the name (*e*.*g*., *Baryonyx 0*). Higher-numbered variations explore the possibility of different values for either *MD* or *Cg*. For example, the *Spinosaurus 1* data point uses the same *MD* as *Spinosaurus 0*, but the value of *Cg* for that point is taken from our attempt to replicate the *Cg* values found by Fabbri *et al*. in Fig 7. The point *Spinosaurus 5* has the same *Cg* as *Spinosaurus 0*, but *MD* is scaled by a factor of 1.64 to reflect allometric scaling from the neotype, which is considered to be 72% of adult size (linear dimensions). The purpose of these alternative test points is to assess the sensitivity of the pFDA results to variations in the test-taxa data. S4 Fig plots these points in parameter space. Abbreviations: *MD*, maximum bone diameter; *Cg*, global bone compactness.

The variations cover three principal approaches. The first covers the maximum diameter *MD*. The *Spinosaurus* neotype has been estimated at 72% of full size. On allometric grounds, one would expect that *MD* would therefore scale by a factor of 1.64 = (1/0.72)^1.5^. This factor follows from the assumption that body mass scales as the cube of linear size (*i*.*e*., isometrically), while *MD* scales as the square root of load. This scaling is not relevant for the other spinosaurids in the analysis, many of which are subadults short of maximum size but not juveniles. However, our scanning of an adult *Suchomimus* femur MNBH GAD500 (S3 Fig) did reveal a quite different maximum diameter (146.4 mm) than that reported by Fabbri *et al*. (120.6 mm), so we use our value as a variation.

The values of *Cg* are also varied based on our attempts to replicate the measurements of Fabbri *et al*. using CT scans, as discussed above and shown in Figs 7, 10 and 11. These hypothetical points are of clear relevance—they are the data points for the taxa that would occur if Fabbri *et al*. measured the *Cg* values the same way we did, or if the specimen had a different but plausible value of *MD*.

In the case of the rib data, we did not have alternative measurements and instead considered a hypothetical scaling of *Cg* by 0.9 (equivalent to a 10% reduction). Seeing as the median percent difference in *Cg* found for multiple specimens of the same taxon (Tables 5 and 6) is 18.6%, we consider this 10% variation to be quite conservative. The 25th percentile of Tables 5 and 6 together is 12.1%, so the hypothetical value of 10% is less than three-quarters of the individual variations. The results of our analysis on the effects of finite sample size on *P*_2_ are presented in Table 9. Example plots of the bootstrap distribution and confidence intervals for the *Spinosaurus* cases are shown in S5–S7 Figs.

**Table 9:** 95% confidence intervals for posterior probability prediction *P*_2_ for spinosaurids.

Dataset	Variant	*F0D0* vs. *F0D2*			Many vs. *F0D2*		
		Median	95% CI		Median	95% CI	
**ds1 (Femur)**	*Baryonyx 0*	0.94	0.83	0.99	0.97	0.90	1.00
	*Baryonyx 1*	0.95	0.85	0.99	0.98	0.92	1.00
	*Baryonyx 2*	0.89	0.76	0.98	0.91	0.82	1.00
	*Baryonyx 3*	0.77	0.59	0.93	0.71	0.57	0.95
	*Spinosaurus 0*	0.97	0.93	1.00	1.00	0.97	1.00
	*Spinosaurus 1*	0.81	0.69	0.94	0.85	0.74	1.00
	*Spinosaurus 2*	0.89	0.79	0.97	0.94	0.85	1.00
	*Spinosaurus 3*	0.93	0.84	0.99	0.97	0.93	1.00
	*Spinosaurus 4*	0.95	0.87	0.99	0.99	0.95	1.00
	*Spinosaurus 5*	0.98	0.92	1.00	0.99	0.98	1.00
	*Spinosaurus 6*	0.83	0.69	0.96	0.83	0.72	1.00
	*Spinosaurus 7*	0.90	0.78	0.98	0.93	0.83	1.00
	*Spinosaurus 8*	0.94	0.85	0.99	0.97	0.90	1.00
	*Spinosaurus 9*	0.95	0.88	1.00	0.98	0.95	1.00
	*Suchomimus 0*	0.49	0.31	0.71	0.25	0.04	0.41
	*Suchomimus 1*	0.31	0.15	0.51	0.09	0.00	0.19
	*Suchomimus 2*	0.50	0.30	0.73	0.25	0.03	0.42
	*Suchomimus 3*	0.32	0.15	0.53	0.09	0.00	0.20
**ds2** **(Rib)**	*Baryonyx 0*	1.00	0.97	1.00	0.97	0.93	1.00
	*Baryonyx 1*	0.97	0.86	1.00	0.89	0.79	0.98
	*Spinosaurus 0*	0.99	0.97	1.00	0.97	0.91	1.00
	*Spinosaurus 1*	0.97	0.89	1.00	0.88	0.79	0.98
	*Spinosaurus 2*	1.00	0.97	1.00	0.97	0.89	1.00
	*Spinosaurus 3*	0.98	0.90	1.00	0.90	0.78	0.98

Using the alternative test-data points of Table 8 and 2000 bootstrap trials, each with 100 randomly generated phylogenetic trees, the posterior probability of membership in class 2 (*F0D2*) was calculated from 200,000 samples. The BCa bootstrap method was used to construct accurate 95% confidence intervals on *P*_2_ for each hypothetical test taxon. Values less than 0.95 are highlighted in yellow. In these cases, using our heuristic approach to classification results (see Materials and methods), we cannot conclude with 95% confidence that the error rate in membership in *F0D2* is 5% or less; membership is thus not supported at the 95% confidence level.

The *P*_2_ results for the original Fabbri *et al*. specimen data for *Baryonyx* (*Baryonyx 0* in ds1, *F0D0* vs. *F0D2* and *Many* vs. *F0D2*, *Baryonyx 0* in ds2, *Many* vs. *F0D2*), and for *Spinosaurus* (*Spinosaurus 0* in ds1, *F0D0* vs *F0D2* and *Spinosaurus 0* in ds2, *Many* vs. *F0D2*) each fail to meet our heuristic criteria that the lower bound of the 95% CI for *P*_2_ is greater than 0.95. Finite-size effects thus undermine the conclusion that there is strong support predicting “subaqueous foraging” for these taxa. As found in the LDA cases of Fig 12, this could be due to intrinsic variation (*i*.*e*., *d/σ* too small), too few data points, or a combination of both.

The sensitivity analysis of hypothetical variations for the spinosaurids results also show that *P*_2_ is highly sensitive to small changes in the values of data points. In the case of *Baryonyx* and *F0D0* vs. *F0D2*, reducing *Cg* to the median value found in Fig 10 (*i*.*e*., *Baryonyx 2* ds1) causes the lower bound on *P*_2_ to drop from 0.83 to 0.76. Using the low *Cg* value from Fig 10 (*i*.*e*., *Baryonyx 3* ds1) results in that lower bound falling further to 0.59. In the *Many* vs. *F0D2* comparison, the latter case results in a still lower *P*_2_ bound of 0.57.

Similar effects are seen in the ds2 (rib) datasets: *Baryonyx 0* has a lower bound of 0.97 for *F0D0* vs. *F0D2*, but this drops to 0.86 in *Baryonyx 1*, in which *Cg* differs by only about 10%. These results show that relatively small variations in *Cg* can shift the expected value of *P*_2_ from significant to dubious.

Qualitatively similar results hold for the variations in *Spinosaurus* and *Suchomimus*. None of the *Spinosaurus* or *Suchomimus* data points, original or variants, ds1 or ds2, have lower bound on *P*_2_ ≥ 0.95 in both *F0D0* vs. *F0D2* and *Many* vs. *F0D2* comparisons. The results for *Spinosaurus* show that the seemingly stringent test of the lower bound of the 95% CI for *P*_2_ ≥ 0.95 can be met, but only for ds2 data and the *F0D0* vs. *F0D2* comparison in the cases of *Spinosaurus 0* and *Spinosaurus 2*, or for ds1 data and *Many* vs. *F0D2* comparison in the cases of *Spinosaurus 0* and the variants numbered 4, 5 and 9.

These sensitivity results show the risk inherent in classifying an entire taxon on a single datapoint (here, either *MD* or *Cg*). The sensitivity analysis shows that the outcome of pFDA can hinge on the precision to which one or both of the data values are known.

The overall result of our analysis of finite-size effects, which occur due to the relatively small number of training datapoints (relative to the variance in those data), greatly reduces our confidence in the key parameters of training-set classification performance, such as *A* and *P*_2_. Our sensitivity analysis shows that even small variations in *Cg* (10%, or less in the case of some of the values from our attempted replication) can have a decisive effect on *P*_2_, and thus on classification.

#### Verification of distribution assumptions for pFDA

Statistical methods have validity only if they are applied to datasets that match the assumptions used in developing the method. Normal statistical practice is to test those assumptions, but Fabbri *et al*. did not report such tests. Here we perform several simple tests of their data distributions.

The pFDA method is based on FDA and LDA, which were originally derived for multivariate normal distributions (Materials and methods). However, we find that the distributions of (log_10_(*MD*), *Cg*) points cannot closely follow a normal distribution in the *Cg* axis because normal distributions are defined on the open interval (−∞, ∞), whereas *Cg* is restricted to the closed interval (0, 1]. The ds1 and ds2 datasets include many values near the top of the range 0.9≤*Cg*<1. As a result, a normal distribution fit to those data will inevitably have a fictitious tail in which the probability density is nonzero for impossible points that have a *Cg*>1. Allocating probability density to illegal values inevitably harms the fit to the distribution elsewhere, even after adjustment for phylogenetic bias.

**Fig 14:**
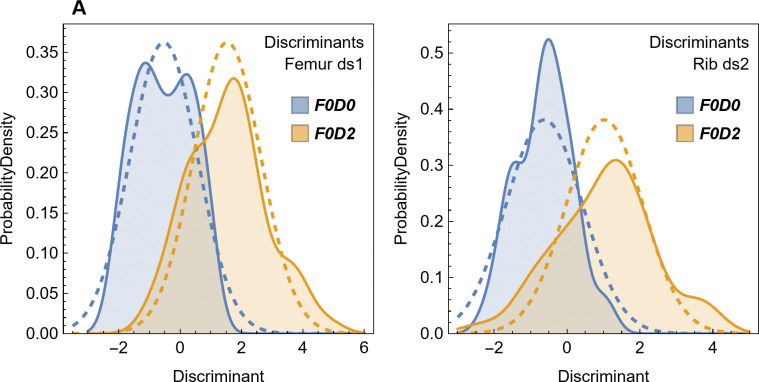
Smoothed kernel distributions for the pFDA discriminants vs. pFDA normal distributions from the (A) femur ds1 and (B) rib ds2 datasets of Fabbri *et al*. [15]. For both datasets, distributions of the discriminants from the *F0D0* and *F0D2* subsets (filled areas) differ from the normal distributions imposed by pFDA (dashed curves), which have different means but the same variance. The distributions from the *F0D0* and *F0D2* groups also overlap considerably in both datasets.

We tested the assumptions about distributions directly by examining the discriminant values generated by the pFDA algorithm, as described in Materials and methods. The discriminant values, which have already been corrected for phylogenetic bias correction and reduced in dimensionality, are directly used to calculate posterior probabilities. It is therefore an important requirement of the method that their distribution is normal. Fig 14 plots the smoothed kernel distributions that we derived from the discriminant values.

Our results show that the discriminants do *not* closely match a normal distribution, particularly when compared to the normal distributions used by pFDA (dashed lines in Fig 14), which are fit to the variance of both classes simultaneously, rather than the best fit to each class. Fig 14 also shows that the discriminants for two groups show considerable overlap, indicating high classification error in the training sets. The overlap in distributions we find here is consistent with the high degree of overlap we demonstrated in the original datasets (Fig 1C and 1D), and with additional analysis we performed using simple effect-size statistics (S8–S10 Figs). We find that correction for phylogenetic bias does not eliminate the overlap between groups, which is to be expected given the very low values of Pagel’s λ found by Fabbri *et al*. The overlap between the *F0D0* and *F0D2* classes is a clear example of the ecological fallacy (S1 Appendix, section 1).

**Fig 15:**
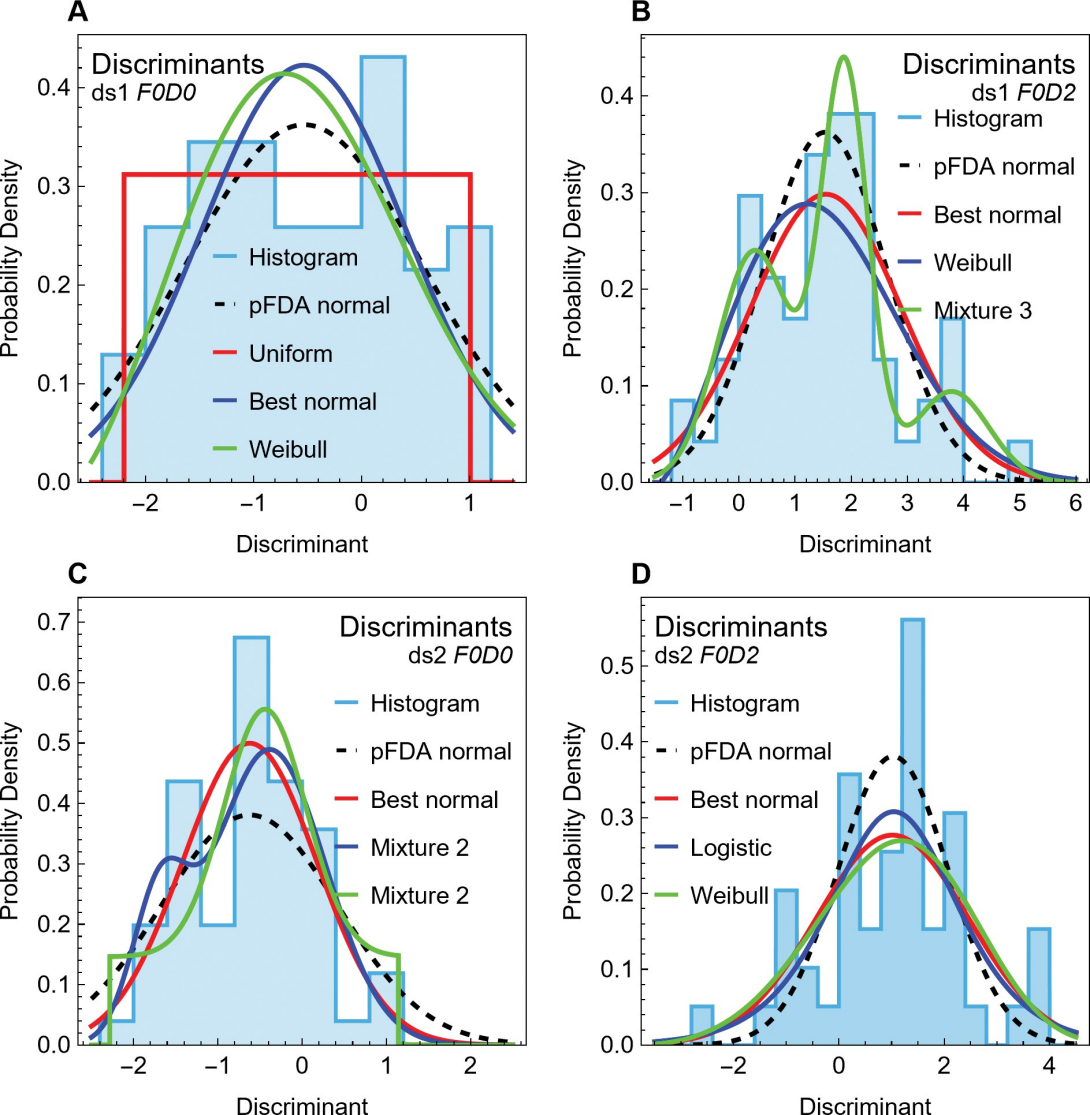
Histograms and fitted distributions for pFDA discriminants. (A, B) Histograms (light blue) of the discriminants for the *F0D0* and *F0D2* subsets of ds1. The pFDA normal distributions (black dashed curves) were computed with a standard deviation parameter pooled across *F0D0* and *F0D2*. The three best-fitting distributions (Table 10) are shown in red, dark blue, and green. (C, D) Comparable plots for ds2.

To quantify the deviation of the discriminants from normality, we made maximum-likelihood estimates of the best-fitting distributions, including standard continuous statistical distributions as well as mixtures of them. Table 10 presents the parameters of the four best-fitting distributions, as well as the normal distributions assumed by pFDA. For a given dataset, pFDA assumes a normal distribution with a different mean for the *F0D0* and *F0D2*, but a standard deviation parameter that is pooled between them. These distributions are plotted in Fig 15.

**Table 10:** Parameters of best-fitting distributions to pFDA discriminants.

Dataset	Dist. type	Weight	Distribution	Param1	Param2	Param3
**ds1** ***F0D0***	Single	n.a.	pFDA normal	−0.531	1.101	
	Single	n.a.	Uniform	−2.198	1.008	
	Single	n.a.	Best normal	−0.533	0.944	
	Single	n.a.	Weibull	2.476	2.42	−2.676
**ds1** ***F0D2***	Single	n.a.	pFDA normal	1.536	1.101	
	Single	n.a.	Best normal	1.55	1.338	
	Single	n.a.	Weibull	2.342	3.331	−1.403
	Mixture 3	0.395	Normal	0.283	0.658	
		0.447	Normal	1.884	0.419	
		0.157	Normal	3.785	0.667	
**ds2** ***F0D0***	Single	n.a.	pFDA normal	−0.623	1.047	
	Single	n.a.	Best normal	−0.622	0.798	
	Mixture 2	0.182	Normal	−1.692	0.324	
		0.818	Normal	−0.384	0.667	
	Mixture 2	0.501	Uniform	−2.282	1.143	
		0.499	Normal	−0.441	0.486	
**ds2** ***F0D2***	Single	n.a.	pFDA normal	1.028	1.047	
	Single	n.a.	Best normal	1.016	1.44	
	Single	n.a.	Logistic	1.042	0.812	
	Single	n.a.	Weibull	4.234	5.954	−4.401

Continuous distributions, as well as mixtures of distributions, were fit via maximum likelihood to the pFDA discriminant data (see text) for each behavioral class (*F0D0* and *F0D2*), using datasets ds1 (femur) and ds2 (rib). Fit parameters, distribution names, and mixture weights (where applicable) are listed for the normal distribution used by pFDA as well as the three other distributions that best fit each dataset. Abbreviations: dist. type: distribution mixture type; param*x*: parameter *x* of the continuous distribution; n.a.: not applicable; pFDA normal: normal distribution with mean calculated separately for *F0D0* and *F0D2* and standard deviation pooled across both classes for that dataset; best normal: best-fitting normal distribution with separate means and standard deviations for the two classes.

Table 11 compares the fits for the distributions of Table 10 to the data by displaying the corrected Akaike information criterion AICc weights of each fit. The Akaike weights *W* can be interpreted as the relative likelihood of each model being best fit [34,152]. We also normalized the weights to generate the relative probability *P*_dist_ of each distribution fitting the data.

**Table 11:** Comparison of distribution fits by AICc for pFDA discriminants.

Dataset	*F0D0*				*F0D2*			
	Distribution	ΔAICc	*W*	*P*_dist_	Distribution	ΔAICc	*W*	*P*_dist_
**ds1 (femur)**	pFDA normal	21.40	2.26 × 10^−5^	2.26 × 10^−5^	pFDA normal	0.49	0.78	0.29
	Uniform	0.00	1.00	1.00	Best normal	0.00	1.00	0.37
	Best normal	19.00	7.50 × 10^−5^	7.50 × 10^−5^	Weibull	0.15	0.93	0.34
	Weibull	18.54	9.40 × 10^−5^	9.40 × 10^−5^	Mixture 3	9.02	0.01	0.00
**ds2 (rib)**	pFDA normal	9.74	0.01	0.01	pFDA normal	4.70	0.10	0.04
	Best normal	0.00	1.00	0.89	Best normal	0.00	1.00	0.44
	Mixture 2	6.25	0.04	0.04	Logistic	0.23	0.89	0.39
	Mixture 2	5.33	0.07	0.06	Weibull	2.36	0.31	0.13

Fits of the distributions and mixtures of Table 10 to the pFDA discriminant data were compared by computing values of the corrected Akaike information criterion (AICc). See Table 10 for descriptions of the distributions. AICc values measure the quality of distribution fits to each data subset; the lowest value of AICc for each subset was subtracted from the other values to calculate the ΔAICc values shown. The distribution having ΔAICc = 0 is the best-fitting distribution. Distributions with ΔAICc < 2 are considered to have strong support; those having ΔAICc ≥ 2 (red shading) are not supported. Akaike weights *W* were normalized to calculate relative probabilities *P*_dist_ of each distribution fitting the specified data subset. Abbreviations: AICc, corrected Akaike information criterion.

Using the standard criteria that ΔAICc<2 indicates strong support, we find that a fit to the uniform distribution is the only choice among the four tested that is strongly supported for ds1 *F0D0*. The normal distribution used by pFDA is not supported and has a low Akaike weight and a *P*_dist_ = 2.26×10^−5^. For ds1 *F0D2*, we found that the pFDA normal, best-fit normal, and uniform distributions all receive strong support under AICc, with the best-fit normal having the strongest support and the pFDA normal the lowest, with *P*_dist_ = 0.29.

The ds2 dataset results show that for the *F0D0* subset, the best-fit normal distribution is the only choice that exhibits strong support under AICc. There is no support for the pFDA normal distribution, which has the lowest *P*_dist_ = 0.01. The ds2 *F0D2* subset is best fit by the best-fit normal, with *P*_dist_ = 0.44, and next the logistic distribution, with *P*_dist_ = 0.39; no other distributions receive strong support, and for the pFDA normal distribution *P*_dist_ = 0.04.

Because pFDA requires both classes in a dataset to fit a normal distribution, the probability that a dataset meets the criteria for comparing classes composed of *F0D0* versus *F0D2* is the product of the *P*_dist_ values for the pFDA normal distributions of each class. For the ds1 dataset, that overall probability is (2.26×10^−5^)×0.29 = 6.50×10^−6^, which is very low. For the ds2 dataset, the probability is 2.84×10^−4^. These results show that both datasets have a low probability of being best fit by the pFDA normal distributions.

Our conservative interpretation is that the distributions do not clearly match the distributional assumptions of pFDA. The results also support a less conservative interpretation: that the datasets are insufficient to clearly and convincingly meet the normal distribution requirement of pFDA, but normality equally cannot be ruled out for some cases. Although a normal distribution fit to each class does have some support in ds1, there is no strong support for ds2 (Table 11).

If the *F0D0* and *F0D2* subsets do not have the same variance—a fundamental prerequisite of both LDA and the subset of FDA used by pFDA—then a final determination of the outcome of normality becomes a moot point. We performed three conventional variance equivalence tests, taking care to choose tests that are robust to deviations from a normal distribution. The test results show that we cannot reject the null hypothesis of equal variances for ds1, but we can reject it for ds2 (Table 12).

**Table 12:** Results of variance-equivalence tests for pFDA discriminants.

Test	ds1 *F0D0* vs. *F0D2*		ds2 *F0D0* vs. *F0D2*	
	Statistic	*P*	Statistic	*P*
**Brown-Forsythe**	2.55	0.11	11.12	0.0012
**Conover**	−1.48	0.14	−3.02	0.0026
**Levene**	2.59	0.11	12.51	0.0006

Three tests of variance equivalence, an assumption of pFDA, were performed on the discriminants of the *F0D0* and *F0D2* groups being compared. These tests were chosen because they are considered robust to deviations from normality in the distributions. The null hypothesis for each test is that variances are equal. This hypothesis can be rejected for both datasets with *P* < 0.05 by all three tests for ds2, but it cannot be rejected for ds1.

In reviewing the results of the distribution fit tests in Tables 10 and 11, we were surprised to find that the uniform distribution is the only distribution for the ds1 *F0D0* subset that has support under AICc. A uniform distribution represents a fundamental challenge to the pFDA paradigm because, like FDA and LDA before it, the pFDA method is based on the assumption that each class has a centroid that is the most probable location for the datapoints of that class. A point is classified by its relative distance from the centroid of each class, as weighted by the normal distribution.

If instead the datapoints have a uniform distribution, then there can be no classification at all because the probability of class membership for a uniform distribution is independent of distance from the centroid. Thus, if a uniform distribution accurately describes any one or more of the classes in a pFDA analysis, classification is impossible. The performance of the uniform distribution in the *F0D0* class of the ds1 femoral dataset indicates that this may be true of that dataset (Tables 10 and 11).

To investigate this question further, we followed standard statistical practice in clustering and classification problems and used the Hopkins statistic to assess whether the points exhibit genuine clustering [36,48,49,153]. Unlike the distribution tests for the discriminants, the Hopkins statistic can be directly computed on the original 2-D data points.

The null hypothesis under this test is that the data points are distributed with a uniform random distribution. Failure to reject the null hypothesis implies that any apparent clustering is likely illusory and attributable to random chance. Table 13 shows the results of our application of the standard Hopkins statistic, as well as two variations by Lawson and Jurs [36] and Fernández Pierna and Massart [35], to the *F0D0* and *F0D2* subsets of both ds1 and ds2 (Materials and methods).

**Table 13:** Results of Hopkins statistic tests and two variations on datasets from Fabbri *et al*. [15].

Dataset	Class subset	Original		FPM		LJ	
		*H*	*P*	*H*	*P*	*H*	*P*
**ds1 (femur)**	*F0D0*	0.459	0.981	0.418	0.890	0.432	0.947
	*F0D2*	0.461	0.830	0.420	0.975	0.391	0.637
	*F0D0*: λ = 0.06	0.465	0.844	0.426	0.978	0.433	0.964
	*F0D2*, λ = 0.06	0.473	0.849	0.405	0.771	0.373	0.389
**ds2 (rib)**	*F0D0*	0.468	0.839	0.419	0.910	0.424	0.938
	*F0D2*	0.447	0.926	0.414	0.993	0.403	0.792
	*F0D0*: λ = 0.07	0.462	0.971	0.420	0.885	0.430	0.927
	*F0D2*, λ = 0.07	0.437	0.925	0.400	0.815	0.399	0.706

Three versions of the Hopkins statistical test for clustering were applied to the *F0D0* and *F0D2* subsets of ds1 and ds2, for both the original datasets and phylogenetically adjusted datasets that are corrected with the same *N* matrix used within pFDA (see Materials and methods), at the optimal values of Pagel’s λ found by Fabbri *et al*. [15]. In each case, the *H* statistic produced by the Hopkins test variant is shown with associated *P* values. Each Hopkins trial was performed on a random sample of 20% of points in the dataset; the *H* statistic is the mean of 100 such trials. The *P* value is calculated for the null hypothesis that the dataset is indistinguishable from a uniform random distribution, evaluated using a Monte Carlo distribution of 10,000 trials drawn from a synthetic uniform distribution. The null hypothesis of a uniform random distribution cannot be rejected in any of the cases. Abbreviations: FPM, Fernández Pierna and Massart variant [35]; LJ, Lawson and Jurs variant [36].

**Fig 16:**
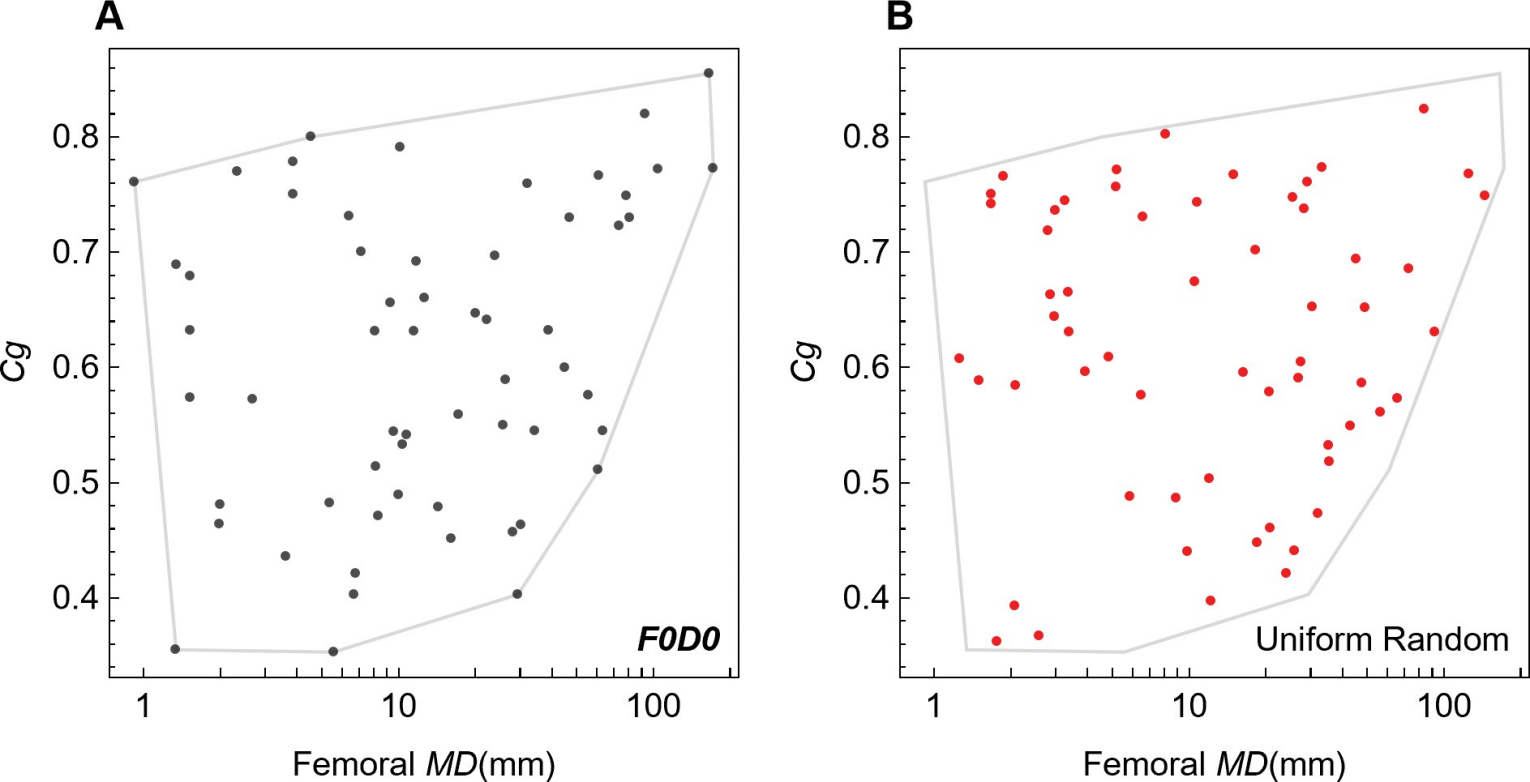
Comparison of data from Fabbri *et al*. [15] to uniform random points. (A) Data points for the terrestrial (*F0D0*) group (black dots) in the femoral dataset of Fabbri *et al*. are plotted along with (B) uniform random points (red dots), both clipped to the convex hull enclosing the *F0D0* data. The apparent absence of any nonrandom concentration or clustering of the *F0D0* data is confirmed by statistical tests (Table 13).

In each case, and for each variation of the Hopkins statistic test, we find that we cannot reject the null hypothesis. The datasets are thus *statistically indistinguishable from a uniform random distribution* in the (log_10_(*MD*), *Cg*) space under the various Hopkins statistic tests. This is true both for the original, untransformed datasets as well as for those that have been phylogenetically corrected using the same optimal values of Pagel’s λ found by Fabbri *et al*. This result is visualized in Fig 16, which shows as one example a plot of *F0D0* from ds1 compared to a uniformly random distribution that has been clipped to the same convex hull.

The relatively strong performance of mixture distributions in Fig 15 and Table 10 suggests that, for some datasets, the distributions might be bimodal. However, the Hopkins-statistic results suggest (but do not prove) that apparent bimodal behavior in the discriminants may be an artifact of the low data count; strongly bimodal distributions would show clustering under the Hopkins statistic.

A uniformly random distribution of data points may seem strange, but biologically this corresponds to the points in (log_10_(*MD*), *Cg*) space being equally likely, at least within some range of values in each parameter. This finding does not falsify the bone ballast hypothesis, which holds that some secondarily semiaquatic adapted taxa will have increased *Cg*. That hypothesis does not specify that the absolute increase must be the same for all aquatically adapted taxa. On the contrary, we would expect the increase in *Cg* to depend on multiple ecological constraints, so the increase should be judged relative to terrestrial sister taxa. Different clades of terrestrial taxa may display a diversity of “typical” *Cg* values [54].

Although increased bone density affects buoyancy, *a priori* we would expect that the optimal body buoyancy depends on taxon-specific factors, such as the typical depth at which an animal operates when underwater [50,54]. The range of depths for which a given taxon is optimized depends on their local environment; it need not be the same for all secondarily aquatic taxa.

Thus, the assumption that the distribution of *Cg* must have a peak value and drop off like a normal distribution from that peak value is arbitrary and not part of the bone ballast hypothesis. Our review of the literature found no prior work suggesting any specific features or properties of the statistical distribution of *Cg* across a broad set of clades. It therefore seems entirely possible that the bone ballast hypothesis holds, even though *Cg* is not normally distributed within each class.

Uniformity in the distribution could have arisen accidentally or been enhanced by choices during dataset construction. Various confounding factors might have led to the subsets mixing taxa that do and do not have increased *Cg*, for example. An attempt to sample a diversity of values of *Cg* and *MD*, covering a range a body sizes, could unintentionally bias selection of taxa for the dataset toward greater spread and less clustering, thereby making a uniform distribution more likely.

The method used to correct phylogenetic bias might also have come into play. If clustering of values in (log_10_(*MD*), *Cg*) space naturally occurs among closely related taxa, then the phylogenetic correction could deemphasize those clusters. While that is the desired effect of removal of phylogenetic bias, it could have the unintended consequence of pushing the dataset toward a uniform random distribution.

Arguably the simplest explanation for the results shown in Table 13 and Fig 16 is low sample size. Datasets that use 49 to 62 points across many clades may simply be too small to show evidence of clustering. Though we offer these general observations, it is beyond the scope of the present study to quantify in detail how the factors above might apply to the datasets under examination.

### Interpretation of lifestyles of extinct “subaqueous foragers” and “nondivers”

When the aim is to discern lifestyle in extinct species, researchers have in the past restricted pFDA training datasets to extant taxa whose lifestyles have been observed. The recent study by Fabbri *et al*. is, to our knowledge, the sole exception to that approach. Their training datasets specify the lifestyle of many extinct species, scoring them as nondiving or as rarely or frequently diving “subaqueous foragers.” For taxa with flippers, such as *Plesiosaurus*, we find this interpretation a reasonable extrapolation based on morphology and paleoenvironment of fossilization. For other taxa, however, such as the extinct hippopotamus *Hexaprotodon garyam*, considerable uncertainty remains regarding its habits in water, as it has fewer secondary aquatic adaptations than the common hippo [132], which forages in terrestrial environments.

Our examination of the datasets found that they do not similarly extrapolate the lifestyle of extinct species that have been long interpreted as fully terrestrial. A large subset of such taxa, 37 nonavian dinosaurs, were scored as nonflying reptiles with “unknown” diving capacity (*F =* 0, *D =* unknown). All nonavian dinosaurs in the analysis were thus treated as “unknown” as to diving status, including *Stegosaurus*, despite its elephantine feet [154]; *Oviraptor*, which is known to have lived and nested in xeric habitats far from any shoreline [155]; and *Alamosaurus*, which had columnar limbs discovered in inland terrestrial deposits [156].

We find the scoring method to be arbitrary, as it scored in advance nearly all subaqueous foragers yet remained blind to well-supported habits of nonspinosaurid nonavian dinosaurs, all of which have long been regarded as fully terrestrial [157]. We find the categorization of these taxa as “unknown” for diving to be a major reason that the terrestrial subset *F0D0* consists almost entirely of extant species.

## Conclusions

The purpose of our study was twofold. First, to contribute to the ongoing debate about the lifestyle of spinosaurids by carefully reexamining the data and methods employed by Fabbri *et al*. in their recent study of the question [15]; we did so at multiple levels and also attempted to replicate some of the measurements and results they published. Second, and perhaps more important, we aimed to identify general issues with the use of pFDA and bone microanatomy metrics such as *Cg* in paleobiology in order to guide future applications of this method and research into ways to improve its utility.

### Conclusions about the results reported by Fabbri *et al.*

The results of our reexamination show that the data and methods of Fabbri *et al*. do not support their conclusion that *Spinosaurus aegyptiacus* and *Baryonyx walkeri* were fully submerged “subaqueous foragers,” whereas *Suchomimus tenerensis* was not. We find that the datasets, groups, and classes they used to compare habitual fully submerged predation to all other lifestyles were constructed in such a way that they cannot be used for accurate classification. The classes show extensive overlap with no division boundary (Fig 1), mix different kinds of foraging behavior, reflect imbalanced choices of extant and extinct taxa (Table 4), include redundant specimens for a few selected taxa, and show a bias toward inclusion of small-bodied exemplars and omission of large-bodied terrestrial taxa more comparable to spinosaurids. We show that in their secondary analysis, Fabbri *et al*. used anatomical criteria to cull “graviportal” and “deep-diving” taxa and then applied those criteria inconsistently, thereby introducing a selection bias in *Cg*.

We identified numerous problems with their choice and use of *Cg* and maximum bone diameter *MD* as the sole variables in a pFDA analysis. We show that *MD* should not have been included as a variable because the ANOVA results reported by Fabbri *et al*. shows that *MD* substantially reduces the explanatory power of the model. We find a worrisome disparity in *Cg* between extinct and extant taxa in their datasets (Table 3), which—coupled with extreme differences in the number of extinct and extant taxa in each class—could bias classification and undermine an assumption of the study. We document many examples of individual variation in *Cg* measured from both extinct (Table 5 and Fig 5) and extant (Table 6) taxa and find that the degree of such variation could account for a majority of the differences Fabbri *et al*. reported between their classes. We describe several biological and taphonomic factors that could lead to such variations (Figs 8 and 9), not only among individual animals but even within single bones (Fig 10). Our attempt to replicate specific measurements of *Cg* from spinosaurid fossils reported by Fabbri *et al*. demonstrates that measurement variation (Figs 7 and 10) and error (Fig 11) also contribute uncertainty that they did not account for in their analysis.

Our examination of how Fabbri *et al*. applied pFDA also reveals several serious statistical problems. Samples sizes are of crucial importance to any statistical analysis, and we find that the datasets and subsets in their study were too small, given the observed differences in *Cg*, to demonstrate results that meet broadly accepted standards of statistical significance (Fig 14 and Table 7), especially when considering issues of biological and measurement variation noted above (Tables 8 and 9). Finally, but perhaps most conclusively, we analyzed the statistical distributions of the data used by Fabbri *et al*., which must conform to normal distributions of equal variance to meet the prerequisites of the pFDA method. We demonstrate that the best-fit distributions to the datasets are not all normal (Figs 14 and 15, Tables 10 and 11), and that their variances are not equal (Table 12). Remarkably, our tests reveal that the datasets are not statistically distinguishable from a uniform random distribution (Table 13 and Fig 16), and we consider a number of plausible factors that could have caused the data to exhibit such scatter.

Many of the results above would be sufficient grounds on their own to question the validity of the conclusions Fabbri *et al*. made about spinosaurid behavior. The unusual constellation of so many different problems allows us to confidently dismiss those findings.

### Conclusions about spinosaurid ecology and lifestyle

Our study did not aim to determine the ecology and lifestyle of *Spinosaurus* and its relatives *Suchomimus* and *Baryonyx*, and our results by themselves do not settle the debate or add new independent lines of evidence about this question. Fabbri *et al*. have highlighted the high *Cg* values in *Spinosaurus*, consistent with the 2014 observation that the Kem Kem specimen has dense, “nearly solid” femora [148]. They have shown that *Baryonyx* also has moderately high *Cg*. We show that in both cases there is some uncertainty; an isolated femur fragment attributed to *Spinosaurus* has a medullary cavity and a much lower *Cg*. With so few specimens of these taxa discovered, conclusions about what is typical are speculative at best.

We find it very unlikely that the high *Cg* values observed in these taxa result from the bone ballast hypothesis. Multiple independent lines of evidence have shown that *Spinosaurus* was unsinkable and too unstable to swim or float, due to its extensive axial pneumaticity [8,14]. The very large body mass of this species offers one obvious alternative explanation, as the correlation of *Cg* to body mass has been well documented in the literature. But the variable infilling observed in the few available specimens greatly limits our ability to draw broad conclusions. Additional lines of evidence [8,13,14] independent from those covered in this study contradict the aquatic pursuit predator hypothesis for *Spinosaurus* [11].

In the present work, we document several ways in which the hypothesis is inconsistent with literature on the bone ballast hypothesis, which tells us that high skeletal *Cg* is more common in slow swimmers or bottom walkers [50,54], including sirenians, sea otters, and hippos. The aquatic pursuit predator hypothesis is also inconsistent with the finding that diving birds, which include fast pursuit predators both with and without flight, tend to exhibit reduced or nonexistent postcranial pneumaticity [101], neither of which have been observed in *Spinosaurus* [14]. But those points are only suggestive—it is the biomechanical evidence of buoyancy, drag, and stability [8,13,14] that together make the strongest case again the aquatic pursuit predator hypothesis.

In the absence of new ideas or new specimens, we conclude that the best current evidence for *Spinosaurus* ecology and lifestyle is marshalled by the most recent papers [8,13,14] that promote the idea of *Spinosaurus* as a semiaquatic piscivore but not an aquatic pursuit predator, as reviewed above in the overview of prior studies on *Spinosaurus* lifestyle. Similarly, the lifestyle of *Baryonyx* [2] and *Suchomimus* [3] is best covered by the earlier work on *Baryonyx* [4].

### Conclusions about the use of *Cg* and pFDA in paleobiology

The bone ballast hypothesis has been considered for many decades, and it remains an important anatomical observation about skeletal adaptation to lifestyle. We found nothing in our study to contradict the idea and evidence that increased bone density (and its proxy, increased *Cg*) are found in taxa with certain specific semiaquatic or aquatic lifestyles, especially slow-swimming taxa such as aquatic herbivores, predators of shellfish, or similarly sessile prey [50,54]. We find the evidence that fast swimmers and active pursuit predators have generally lower bone density and *Cg* [50,54] to be consistent with the bone ballast hypothesis as well.

However, the relationship between *Cg* and semiaquatic or fully aquatic taxa via the bone ballast hypothesis is complex, and the hypothesis has its limits [52]. Bone density and *Cg* are potentially increased by other attributes and lifestyles of a taxon. Of the many potential confounding factors, the most relevant to spinosaurids is large body size, which has been associated with high *Cg* independent of semiaquatic adaptations [50,52,54,107,132]. The example of extant hippos shows that we may not be able to distinguish between these two effects [132].

Bone ballast measured via *Cg* sampled only from long bones or ribs may be sufficiently diagnostic for reptiles or mammals, but such data are not sufficient for classifying birds or nonavian dinosaurs, which have extensive pneumaticity [99]. In these taxa, the negative buoyancy effect of dense ribs or femora is at least partly offset by the positive buoyancy of air sacs found in bird’s paraxial pneumaticity. New techniques, including 3-D models that include flesh and air sacs, may be needed to supplement data on bone microanatomy metrics.

Our review of the literature investigating the bone ballast hypothesis found that this has *not* previously been proposed as a universal rule across amniotes. Prior studies instead qualify it is as a phenomenon found in only in specific niches [50,54]. Despite this, Fabbri *et al*. made a tantalizing assertion in multiple places in their study that their findings do hold across all amniotes—or alternatively among all amniotes except graviportal and deep-diving taxa. Their attempt to marshal statistical support for such a near-universal rule may have resulted in some of the problematic choices of data and technique that we found in this work, including the selection of taxa that are clearly inappropriate for direct comparison to spinosaurids, such as taxa without legs or of tiny body size.

Outside of a few well-known scaling relationships in macroecology [158–160], relationships this broad are rare. Moreover, the extensive literature on the bone ballast hypothesis, including studies [50] referenced by Fabbri *et al*., clearly describes many exceptions and alternatives that confound the formulation of a single universal rule. A lesson for future studies is that great care must be taken when drawing sweeping conclusions, particularly if they are contradicted by the available literature or miss large groups that are central to the analysis. Another lesson is that comparative statistical analysis aimed a specific group (“carnivorous dinosaurs” in the case of Fabbri *et al*.) cannot be done properly by using broad amniote-wide databases. Instead, datasets must be tailored to the details of the questions being asked in the analysis. This includes having direct biomechanical relevance to the question at hand; tiny shrews and voles, animals that lack legs, and herbivores arguably have little in common with a giant predatory dinosaur.

In our study, we considered whether the analytical approach adopted by Fabbri *et al*. could be improved by certain changes that would render its results valid. We conclude that it cannot, for multiple reasons. Perhaps the most important of these is that bone microanatomy data on ribs and femora is fundamentally not able to capture the buoyancy effect of the pneumaticity found in the vertebral column in the spinosaurids. Put another way, the bone ballast hypothesis was not formulated for dinosaurs in which the ballast effect is dominated by pneumaticity, which cannot be assessed using rib and femoral data alone.

Including pneumatized taxa in an analysis of the bone ballast hypothesis is not simple and should be the topic of future research. To understand the net influence of different buoyancy effects, it is not enough to analyze bone density (*Cg*) via a simple regression; one also needs to perform a detailed study of the flesh, bone, and air-sac mass in each taxon—ideally employing a full digital model—as opposing factors that must be quantitatively compared. Such a study has now been published for *Spinosaurus*, and the results are overwhelmingly incompatible with an ability to submerge or swim underwater [14]. To repeat a study of this kind for *Baryonyx* or *Suchomimus*—let alone broad datasets of other taxa for comparison—is beyond the scope of the present study but would be valuable, though in the case of *Baryonyx* it might require more complete skeletal material than currently exists.

While the search for new specimens continues, future research is needed to determine whether, or how much, *Cg* varies among individual specimens, through ontogeny, across different skeletal elements, or even between different cross sections of the same bone. We were unable to find any foundational studies that have collected sufficient data to accurately characterize the expected variation in all of these dimensions. However, the variations we did find in the literature and in our own replication study show that variation poses a significant risk to studies that depend on quantitative differences in *Cg*. Future studies should take variation into careful account, and prior studies that performed regression, ANOVA, or LDA on datasets that represent each taxon by a single *Cg* value may need to be revisited. The subjective factors that complicate replication of *Cg* measurements—and might have led to the anomalous values and selection bias that we found in the Fabbri *et al*. datasets—also deserve more research.

The question of whether fossil specimens have a systematically different distribution of *Cg* than extant taxa do must also be resolved, with attention to factors such as matrix infilling and damage repair. The Fabbri *et al*. femoral dataset and the prior studied from which it was compiled is possibly the largest collection of samples ever assembled for which someone has compared the extant and extinct taxa *Cg* values statistically, as done here. Although the dataset was not created to explore differences in *Cg* measured from extinct and extant taxa, a strong bias toward extinct specimens is evident at the high end of the *Cg* range. Whether that is a real effect or an artifact of selection bias should be investigated.

We have identified other pitfalls that complicate the use of the pFDA method in paleobiology. The method does not test whether input datasets are suitable, for example. The present study seems to be the first to systematically test the normality assumption on the discriminants and to assess the effects of the training dataset size on the results. Our results show that sample size, a largely neglected factor in previous applications of LDA and pFDA in biology, affects classification in ways that are important to quantify. Our Monte Carlo analyses illustrate important principles and metrics that can be applied to evaluate whether datasets are large and normal enough to support the use of pFDA to make inferences.

Because pFDA does not produce *P* values or other quantitative estimates of statistical error, the results it produces must be interpreted with caution. In this study, we examined several metrics, including training-set accuracy *A*, the *P*_2_ probability of class membership, and a heuristic concept we introduced as *P*_rand_, the equivalent probability of random classification versus correct classification. We show that 75% training-set accuracy *A* is equivalent to *P*_rand_ = 0.5, meaning a classifier that is random half the time and correct the other half. *P*_rand_ offers one way to apply conventional thresholds of statistical confidence and significance; the 95% confidence level, for example, is heuristically equivalent to *P*_rand_ ≤ 0.05. But more work must be done to develop formal mathematical metrics of classification performance.

Future pFDA studies that use datasets for which the method is better suited may obtain *P*_rand_ values closer to 0.05 and may be able to draw clear decision boundary lines like those of Fig 1C and 1D. Statistical research is needed to determine what criteria for statistical significance are appropriate for pFDA studies. Until these questions are answered, it will remain difficult for paleontologists to interpret whether pFDA results have the same degree of statistical power and rigor as other statistical methods.

## Supporting information

S1 Fig Correlation between global bone compactness (*Cg*) and femoral maximum diameter (*MD*) in Sauropterygia. Fabbri *et al*. [15] include data from six specimens of *Nothosaurus*, two of the related nothosaur *Simosaurus*, and one related pachypleurosaur, *Serpicosaurus*. Each point is labeled with the identifier used in the Fabbri *et al*. datasets. A strong inverse correlation is shown between global bone compactness (*Cg*) and femoral *MD*, which is commonly used as a proxy for body size. The blue regression line only includes data points for *Nothosaurus*, the black regression line includes all taxa in the plot. Regression parameters are shown in the inset table. The coefficient of determination is extremely high (*R*^2^ = 0.96) for *Nothosaurus* alone but still very high (*R*^2^ = 0.84) for these sauropterygian taxa pooled together. The source of this strong trend is unknown to us; it could be a real biological effect, or a data artifact, or some combination thereof. If extrapolated, these trends would have *Cg* = 0 at *MD* = 103 mm for *Nothosaurus* and *MD* = 108 mm for all taxa, which is biologically impossible.
(TIF)

S2 Fig *Spinosaurus* aegyptiacus subadult femur retaining medullary cavity (CMN 41869). (A) Proximal half of the right femur in medial view. (B) Medullary cavity in ventrolateral view. (C) Bone lining the medullary cavity. Abbreviations: at, anterior trochanter; cb, cancellous bone; ft, fourth trochanter; hd, head; mc, medullary cavity.
(TIF)

S3 Fig *Suchomimus tenerensis* juvenile femoral mid shaft thin section. Thin section of the midshaft of a right femur of a juvenile individual (femur length 55.3 cm; MNBH GAD72).
(TIF)

S4 Fig Variant datapoints for the femoral ds1 dataset from Table 8. The hypothetical spinosaurid datapoints for femoral data (ds1) from Table 8 are plotted by *MD* and *Cg* to illustrate the effect of the variations. Numbers correspond to the variation suffixes in Table 8; the original datapoints used by *Fabbri et al*. are labelled with *Sp* for *Spinosaurus*, *Su* for *Suchomimus*, and *Ba* for *Baryonyx*. Points are colored according to the legend. Arrows indicate how far each of the variations is displaced in *MD* and *Cg* from the original data point.
(TIF)

S5 Fig Bootstrap distributions of *P*_*2*_ for *Spinosaurus* sensitivity cases 0–3. Bootstrap analysis was used with 2000 trials to predict *P*_*2*_, the posterior probability of *Spinosaurus* belonging to the class of “subaqueous foragers.” Each bootstrap trial contains the results of 100 random trees, so there are a total of 200,000 predictions. Histograms show the distribution of *P*_*2*_ for the *Spinosaurus* sensitivity analysis variations 0–3 of Table 8. Vertical gray lines and numbers along the top of each chart show the medians and their 95% CI, as determined by the BCa bootstrap confidence integral algorithm. Vertical red lines show the 2.5%, 50%, and 97.5% quantiles of the bootstrap distributions of *P*_*2*_. In a case where the bootstrap distribution has the same median as the original dataset prior to bootstrapping, there would be no bias. In general, however, bootstrapping can introduce bias. The BCa bootstrap algorithm adjusts the bias and also corrects for nonconstant variance. As a result, the BCa 95% CI does not always line up with the quantiles of the bootstrap distribution (*i*.*e*., gray lines and red lines may not overlap).
(TIF)

S6 Fig Bootstrap distributions of *P*_*2*_ for *Spinosaurus* sensitivity cases 4–7. Bootstrap analysis was used with 2000 trials to predict *P*_*2*_, the posterior probability of *Spinosaurus* belonging to the class of “subaqueous foragers.” Each bootstrap trial contains the results of 100 random trees, so there are a total of 200,000 predictions. Histograms show the distribution of *P*_*2*_ for the *Spinosaurus* sensitivity analysis variations 4–7 of Table 8. Vertical gray lines and numbers along the top of each chart show the medians and their 95% CI, as determined by the BCa bootstrap confidence integral algorithm. Vertical red lines show the 2.5%, 50%, and 97.5% quantiles of the bootstrap distributions of *P*_*2*_. In a case where the bootstrap distribution has the same median as the original dataset prior to bootstrapping, there would be no bias. In general, however, bootstrapping can introduce bias. The BCa bootstrap algorithm adjusts the bias and also corrects for nonconstant variance. As a result, the BCa 95% CI does not always line up with the quantiles of the bootstrap distribution (*i*.*e*., gray lines and red lines may not overlap).
(TIF)

S7 Fig Bootstrap distributions of *P*_*2*_ for *Spinosaurus* sensitivity cases 8 and 9. Bootstrap analysis was used with 2000 trials to predict *P*_*2*_, the posterior probability of *Spinosaurus* belonging to the class of “subaqueous foragers.” Each bootstrap trial contains the results of 100 random trees, so there are a total of 200,000 predictions. Histograms show the distribution of *P*_*2*_ for the *Spinosaurus* sensitivity analysis variations 8 and 9 of Table 8. Vertical gray lines and numbers along the top of each chart show the medians and their 95% CI, as determined by the BCa bootstrap confidence integral algorithm. Vertical red lines show the 2.5%, 50%, and 97.5% quantiles of the bootstrap distributions of *P*_*2*_. In a case where the bootstrap distribution has the same median as the original dataset prior to bootstrapping, there would be no bias. In general, however, bootstrapping can introduce bias. The BCa bootstrap algorithm adjusts the bias and also corrects for nonconstant variance. As a result, the BCa 95% CI does not always line up with the quantiles of the bootstrap distribution (*i*.*e*., gray lines and red lines may not overlap).
(TIF)

S8 Fig One-dimensional and two-dimensional effect size statistics on the Fabbri *et al*. and corrected training datasets, compared to original and remeasured spinosaurid values. (A) The plots use bars to show the 95% confidence interval and 95% single-prediction intervals for the mean value of *Cg* in the femoral and rib training sets. Within each training set, the intervals for the *F0D0* group are shown as red (95% CI) and pink (95% prediction) bars; the intervals for *F0D2* are shown in blue and cyan, respectively. The values for spinosaurid taxa used in Fabbri *et al*. [15] are marked with solid black markers. The confidence and prediction intervals for the mean provide a simple one-dimensional view of the overlap in distributions between the *F0D0* and *F0D2* groups. In the femoral dataset, the 95% confidence interval of the mean of *F0D2* lies entirely within the prediction interval of *F0D0*, showing that even the mean *Cg* in *F0D2* would be plausible as a member of *F0D0*. In the rib dataset, the mean 95% CI for *F0D2* is mostly within the prediction interval for *F0D0*. The 95% CI for the mean of *F0D0* overlaps with the prediction interval of *F0D2* for femoral data and falls entirely within the interval for rib data. In each case we see that an average value of *Cg* distribution of one group (say, *F0D2* divers) is plausible as a member of the opposite group (the *F0D0* nondivers) and vice versa. The overlap in *Cg* for the groups occurs not only at the edge cases of a group but also extends to group average. (B) Linear regressions (performed without phylogenetic bias adjustment) of (*Cg*, Log(10, *MD*)) are plotted with their with 95% prediction interval for the *F0D0* and *F0D2* groups of femoral and rib datasets. Outputs for *R*^2^ from the lm() function in R are reported. The two-dimensional intervals show that the overlap evident in the *Cg* plots of (A) is also present when diameter is considered. The regression results show that these two-dimensional regressions have extremely weak correlation and have somewhat minor impact on our interpretations, although they often produce *F0D0* 95% prediction intervals even closer to *Spinosaurus* values in the bivariate space. The weak correlations support the conclusion by both Fabbri *et al*. and ourselves that including bone diameter likely does not improve the predictive ability of the model.
(TIF)

S9 Fig Quantile-quantile plots of pFDA discriminants from dataset ds1 subsets *F0D0* and *F0D2*. In these panels, the quantiles of the discriminant distributions versus those of a normal or uniform distribution (heavy black points) can be compared to plots of the normal or uniform distribution with itself (thin dotted lines).
(TIF)

S10 Fig Quantile-quantile plots of pFDA discriminants from dataset ds2 subsets *F0D0* and *F0D2*. In these panels, the quantiles of the discriminant distributions versus those of a normal or uniform distribution (heavy black points) can be compared to plots of the normal or uniform distribution with itself (thin dotted lines).
(TIF)

S1 Table Settings for computed-tomographic scans of each of the specimens described. Links are provided to Morphosource records containing CT scans created for this study.
(DOCX)

S1 File Femur compactness all. This data file, which is processed by the R script of Fabbri *et al*., contains the full femoral dataset for that study. We denote this dataset ds1 in our study.
(CSV)

S2 File Rib compactness all. This data file, which is processed by the R script of Fabbri *et al*., contains the full rib dataset for that study. We denote this dataset ds2 in our study.
(CSV)

S3 File Femur compactness no graviportals no pelagics. This data file, which is processed by the R script of Fabbri *et al*., contains a femoral dataset that was reduced by elimination of selected taxa deemed graviportal or deep-diving. We denote this dataset ds3 in our study.
(CSV)

S4 File Rib compactness no graviportals_no pelagics. This data file, which is processed by the R script of Fabbri *et al*., contains a rib dataset that was reduced by elimination of selected taxa deemed graviportal or deep-diving. We denote this dataset ds4 in our study.
(CSV)

S1 Appendix Supporting information on methodological issues. The Appendix provides additional details, figures, and equations elaborating on our methods and results, in five sections: (1) the ecological fallacy; (2) ROC curves and whether P_2_>0.5 is the best threshold; (3) *P* values and the *p*<0.05 threshold; (4) classification performance metrics; and (5) a bug or misunderstanding in pFDA codes.
(DOCX)
