# Supplementary figures and images for: Diving dinosaurs? Caveats on the use of bone compactness and pFDA for inferring lifestyle
Source: PLoS One. 2024 Mar 6;19(3):e0298957. doi: 10.1371/journal.pone.0298957 (PMC10917332; doi:10.1371/journal.pone.0298957)

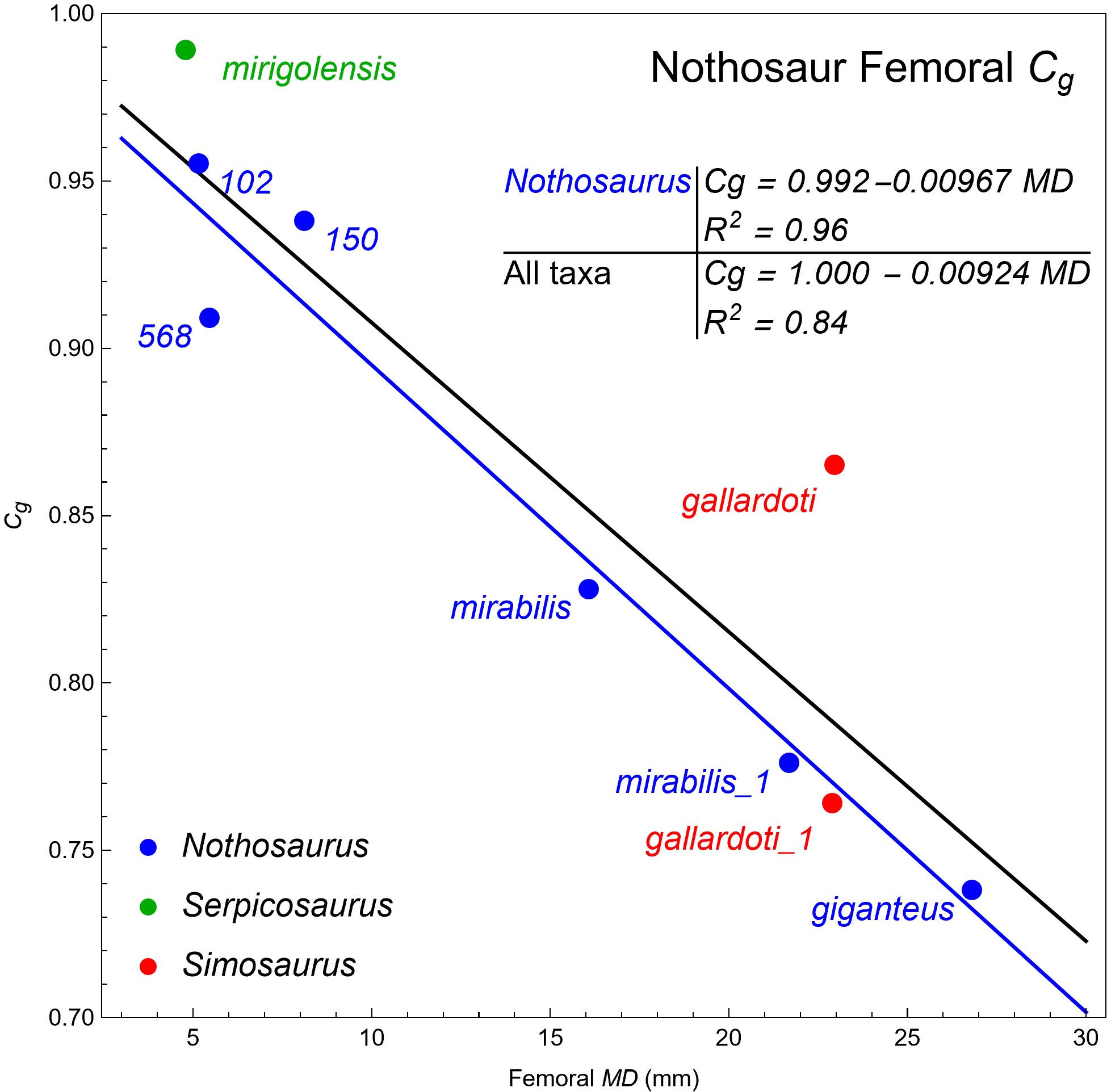

Supplement: S1 Fig — Fabbri et al. [15] include data from six specimens of Nothosaurus, two of the related nothosaur Simosaurus, and one related pachypleurosaur, Serpicosaurus. Each point is labeled with the identifier used in the Fabbri et al. datasets. A strong inverse correlation is shown between global bone compactness (Cg) and femoral MD, which is commonly used as a proxy for body size. The blue regression line only includes data points for Nothosaurus, the black regression line includes all taxa in the plot. Regression parameters are shown in the inset table. The coefficient of determination is extremely high (R2 = 0.96) for Nothosaurus alone but still very high (R2 = 0.84) for these sauropterygian taxa pooled together. The source of this strong trend is unknown to us; it could be a real biological effect, or a data artifact, or some combination thereof. If extrapolated, these trends would have Cg = 0 at MD = 103 mm for Nothosaurus and MD = 108 mm for all taxa, which is biologically impossible. (TIF) [file pone.0298957.s001.tif]

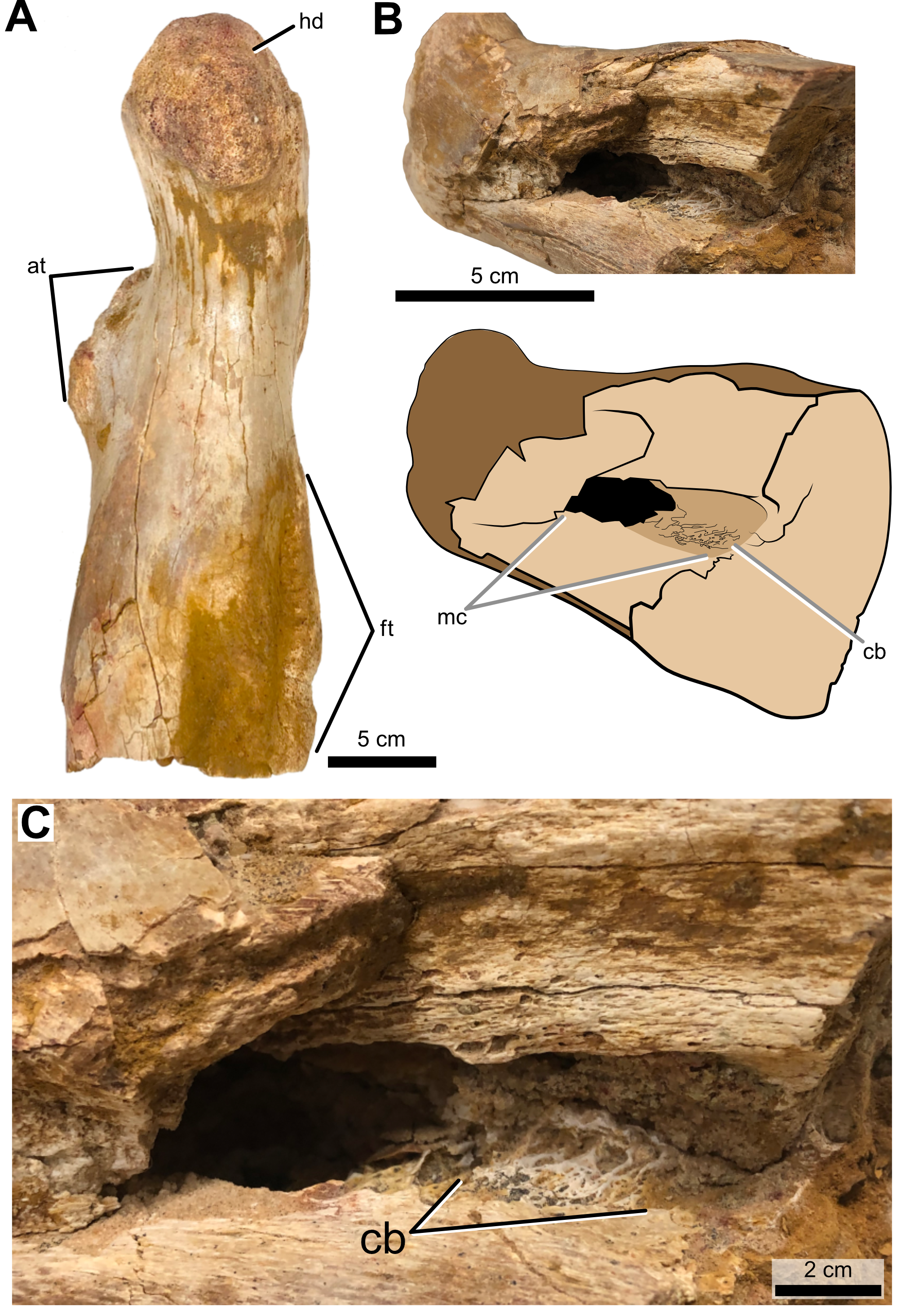

Supplement: S2 Fig — (A) Proximal half of the right femur in medial view. (B) Medullary cavity in ventrolateral view. (C) Bone lining the medullary cavity. Abbreviations: at, anterior trochanter; cb, cancellous bone; ft, fourth trochanter; hd, head; mc, medullary cavity. (TIF) [file pone.0298957.s002.tif]

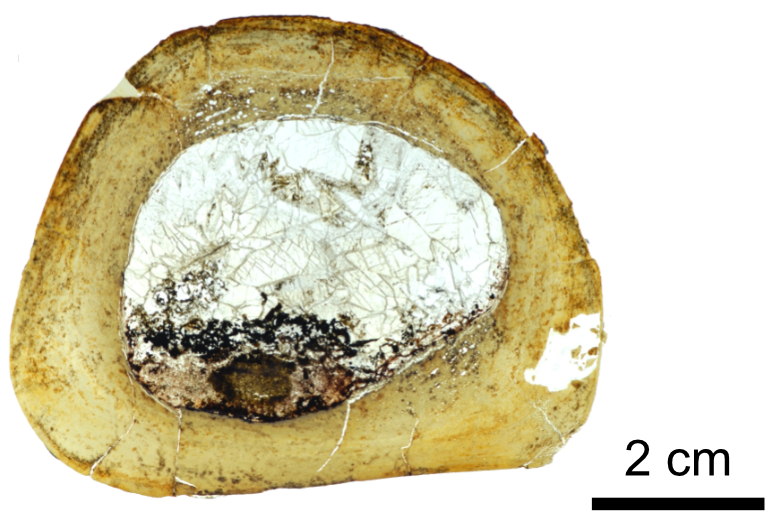

Supplement: S3 Fig — Thin section of the midshaft of a right femur of a juvenile individual (femur length 55.3 cm; MNBH GAD72). (TIF) [file pone.0298957.s003.tif]

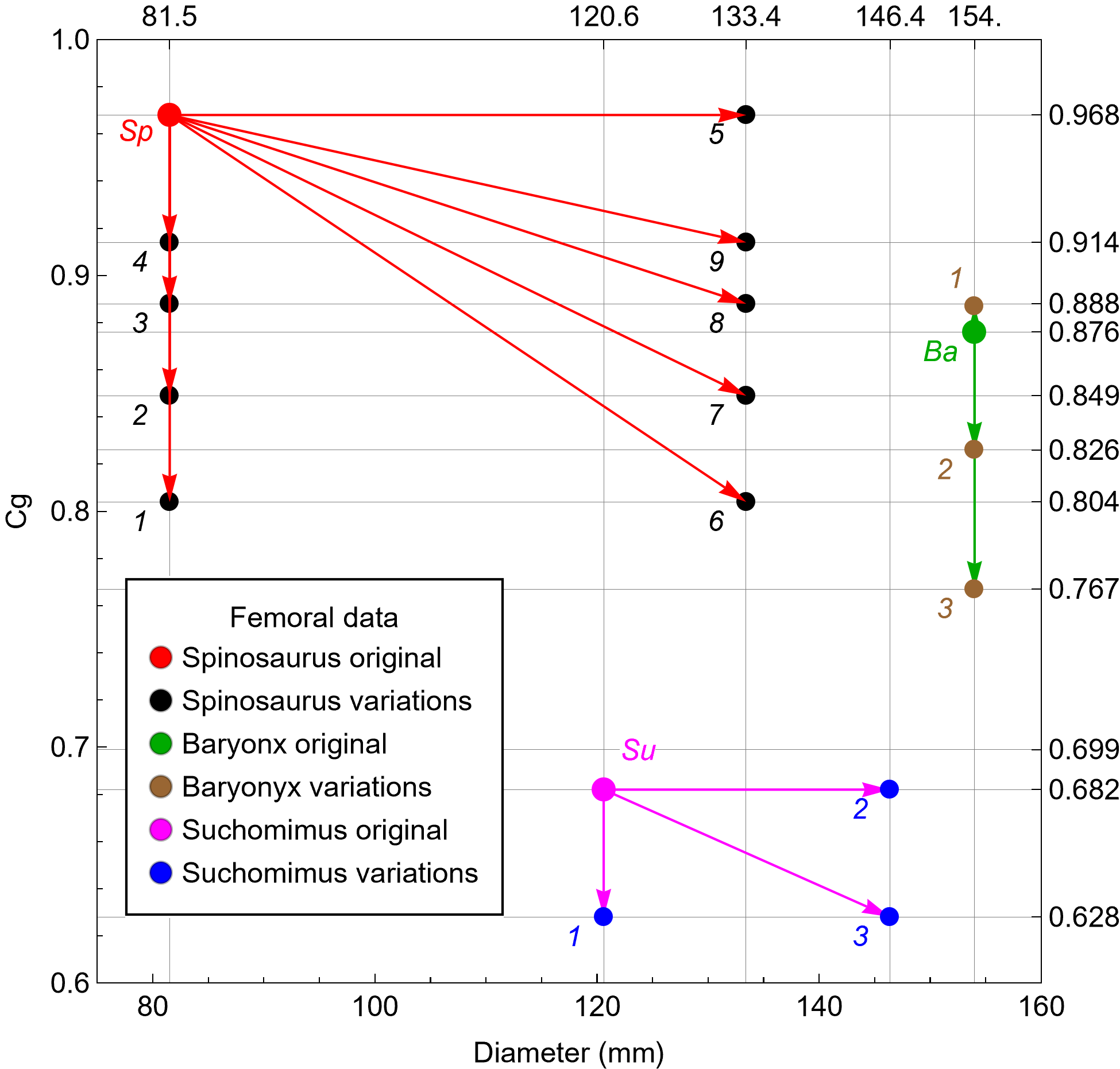

Supplement: S4 Fig — The hypothetical spinosaurid datapoints for femoral data (ds1) from Table 8 are plotted by MD and Cg to illustrate the effect of the variations. Numbers correspond to the variation suffixes in Table 8; the original datapoints used by Fabbri et al. are labelled with Sp for Spinosaurus, Su for Suchomimus, and Ba for Baryonyx. Points are colored according to the legend. Arrows indicate how far each of the variations is displaced in MD and Cg from the original data point. (TIF) [file pone.0298957.s004.tif]

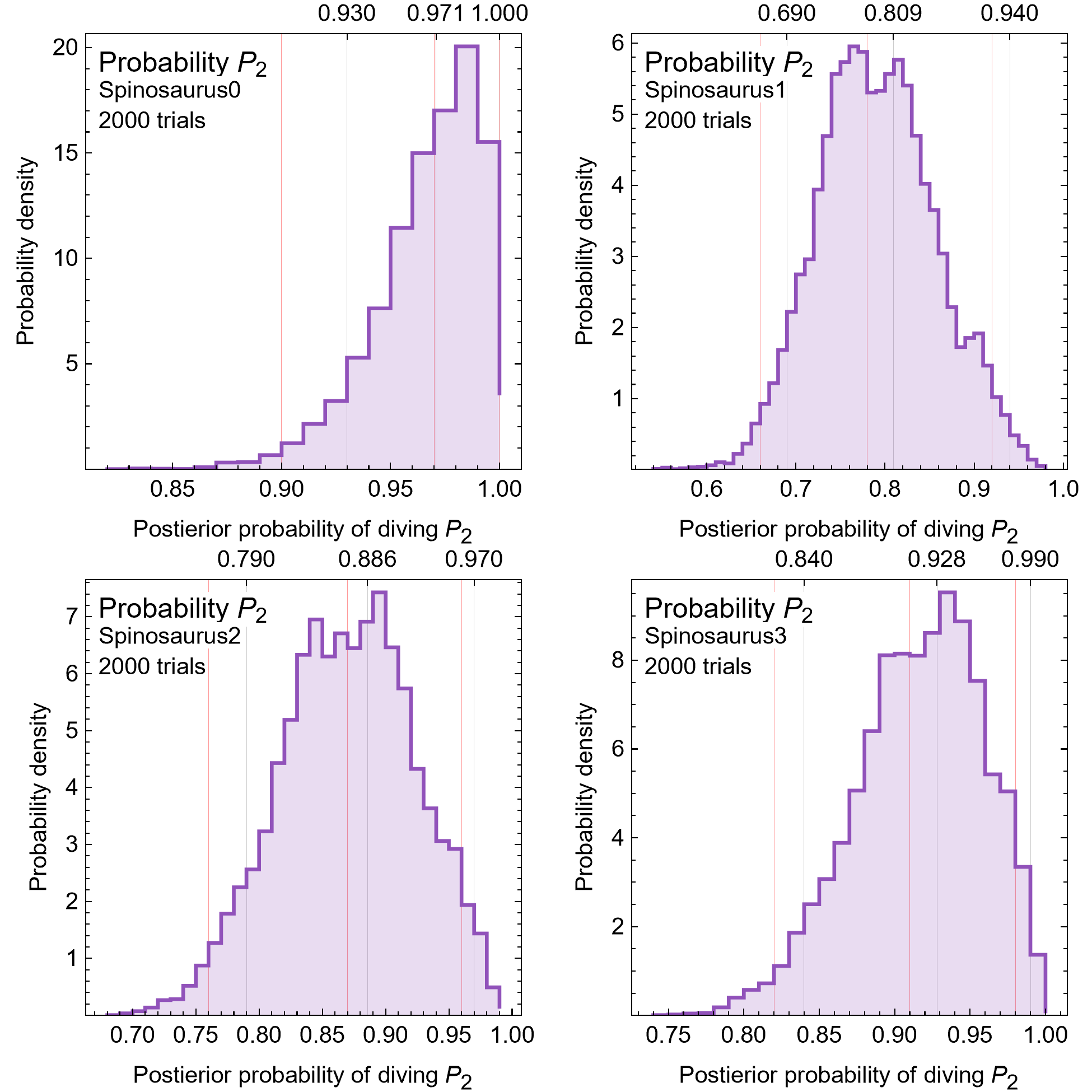

Supplement: S5 Fig — Bootstrap analysis was used with 2000 trials to predict P2, the posterior probability of Spinosaurus belonging to the class of “subaqueous foragers.” Each bootstrap trial contains the results of 100 random trees, so there are a total of 200,000 predictions. Histograms show the distribution of P2 for the Spinosaurus sensitivity analysis variations 0–3 of Table 8. Vertical gray lines and numbers along the top of each chart show the medians and their 95% CI, as determined by the BCa bootstrap confidence integral algorithm. Vertical red lines show the 2.5%, 50%, and 97.5% quantiles of the bootstrap distributions of P2. In a case where the bootstrap distribution has the same median as the original dataset prior to bootstrapping, there would be no bias. In general, however, bootstrapping can introduce bias. The BCa bootstrap algorithm adjusts the bias and also corrects for nonconstant variance. As a result, the BCa 95% CI does not always line up with the quantiles of the bootstrap distribution (i.e., gray lines and red lines may not overlap). (TIF) [file pone.0298957.s005.tif]

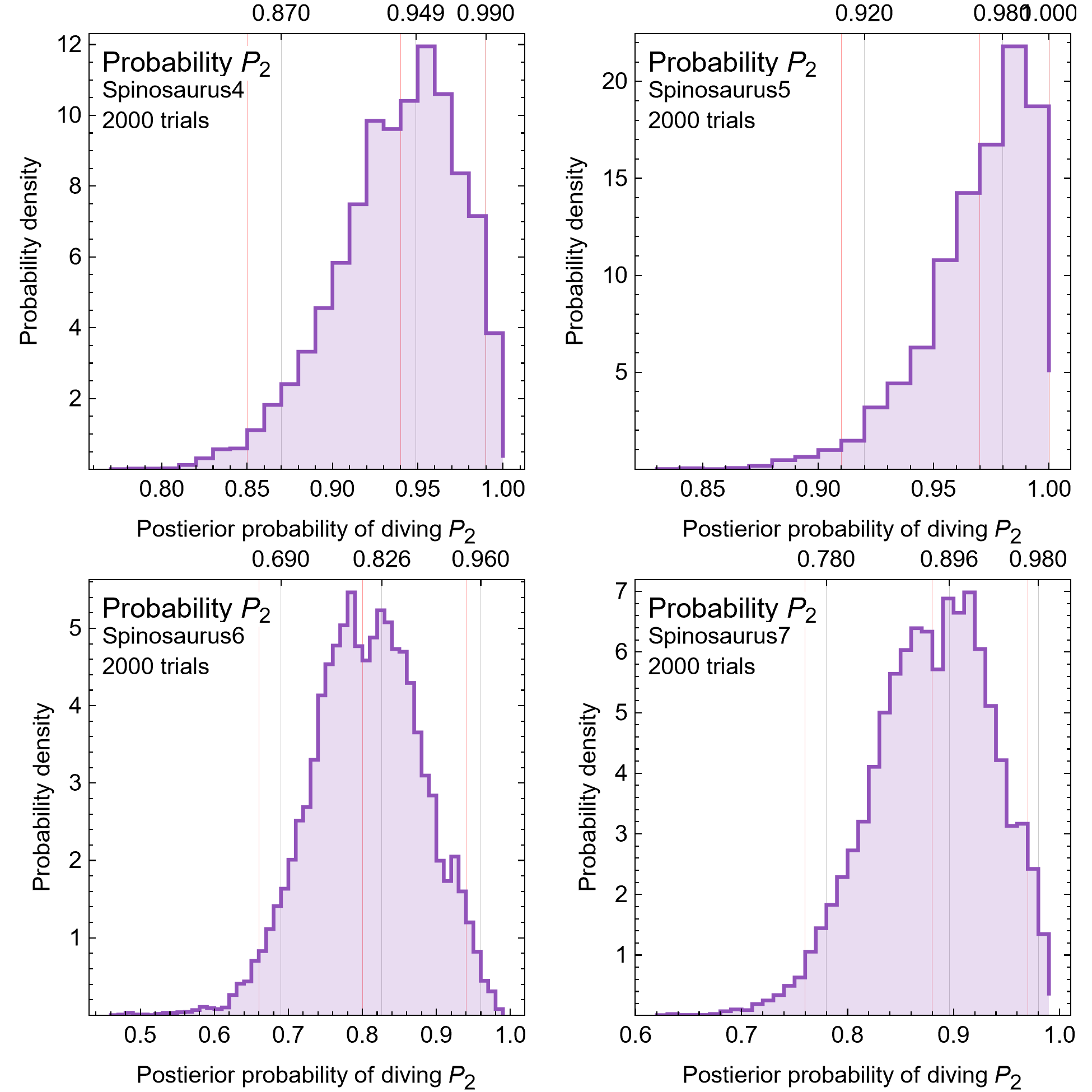

Supplement: S6 Fig — Bootstrap analysis was used with 2000 trials to predict P2, the posterior probability of Spinosaurus belonging to the class of “subaqueous foragers.” Each bootstrap trial contains the results of 100 random trees, so there are a total of 200,000 predictions. Histograms show the distribution of P2 for the Spinosaurus sensitivity analysis variations 4–7 of Table 8. Vertical gray lines and numbers along the top of each chart show the medians and their 95% CI, as determined by the BCa bootstrap confidence integral algorithm. Vertical red lines show the 2.5%, 50%, and 97.5% quantiles of the bootstrap distributions of P2. In a case where the bootstrap distribution has the same median as the original dataset prior to bootstrapping, there would be no bias. In general, however, bootstrapping can introduce bias. The BCa bootstrap algorithm adjusts the bias and also corrects for nonconstant variance. As a result, the BCa 95% CI does not always line up with the quantiles of the bootstrap distribution (i.e., gray lines and red lines may not overlap). (TIF) [file pone.0298957.s006.tif]

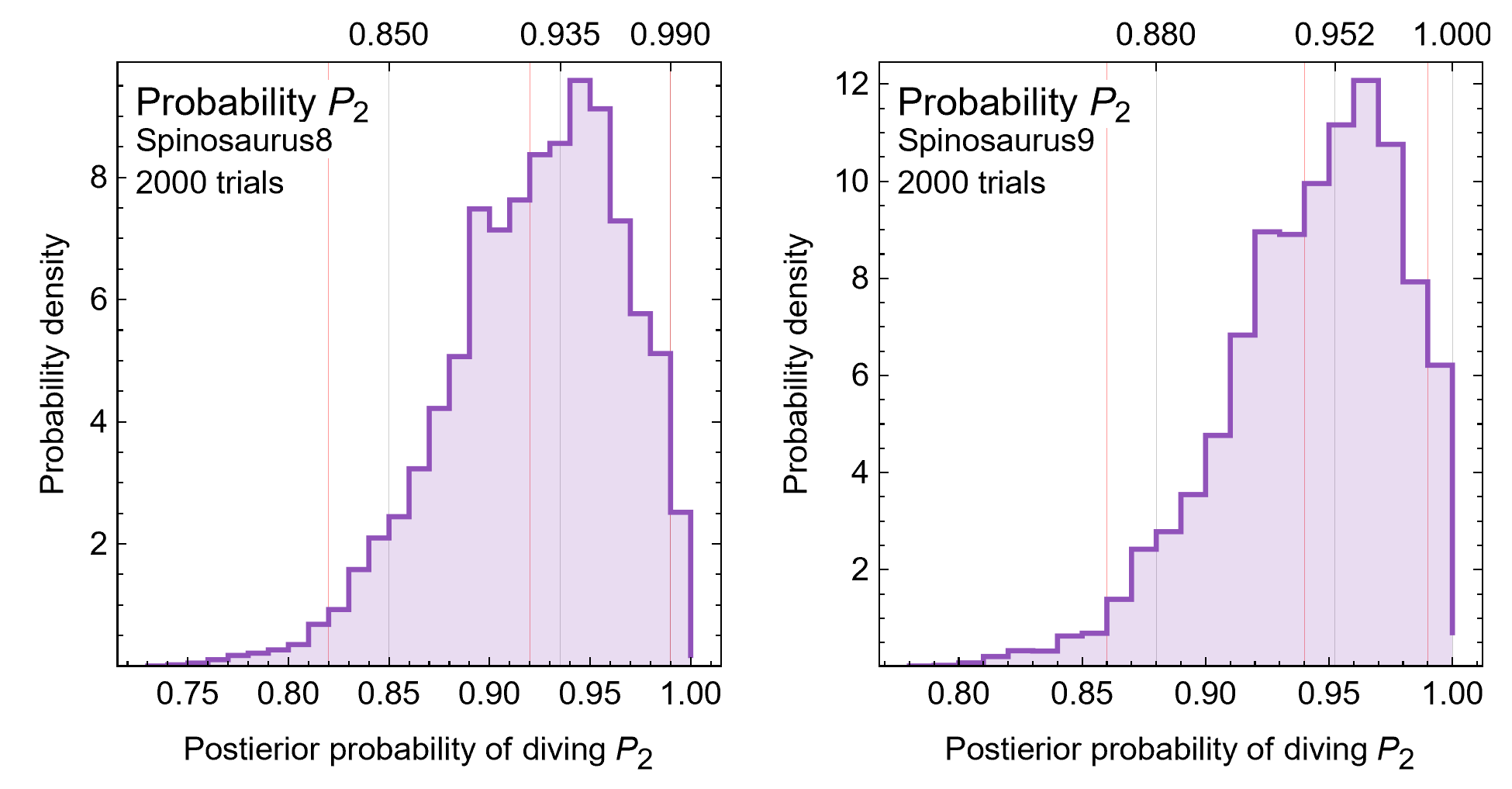

Supplement: S7 Fig — Bootstrap analysis was used with 2000 trials to predict P2, the posterior probability of Spinosaurus belonging to the class of “subaqueous foragers.” Each bootstrap trial contains the results of 100 random trees, so there are a total of 200,000 predictions. Histograms show the distribution of P2 for the Spinosaurus sensitivity analysis variations 8 and 9 of Table 8. Vertical gray lines and numbers along the top of each chart show the medians and their 95% CI, as determined by the BCa bootstrap confidence integral algorithm. Vertical red lines show the 2.5%, 50%, and 97.5% quantiles of the bootstrap distributions of P2. In a case where the bootstrap distribution has the same median as the original dataset prior to bootstrapping, there would be no bias. In general, however, bootstrapping can introduce bias. The BCa bootstrap algorithm adjusts the bias and also corrects for nonconstant variance. As a result, the BCa 95% CI does not always line up with the quantiles of the bootstrap distribution (i.e., gray lines and red lines may not overlap). (TIF) [file pone.0298957.s007.tif]

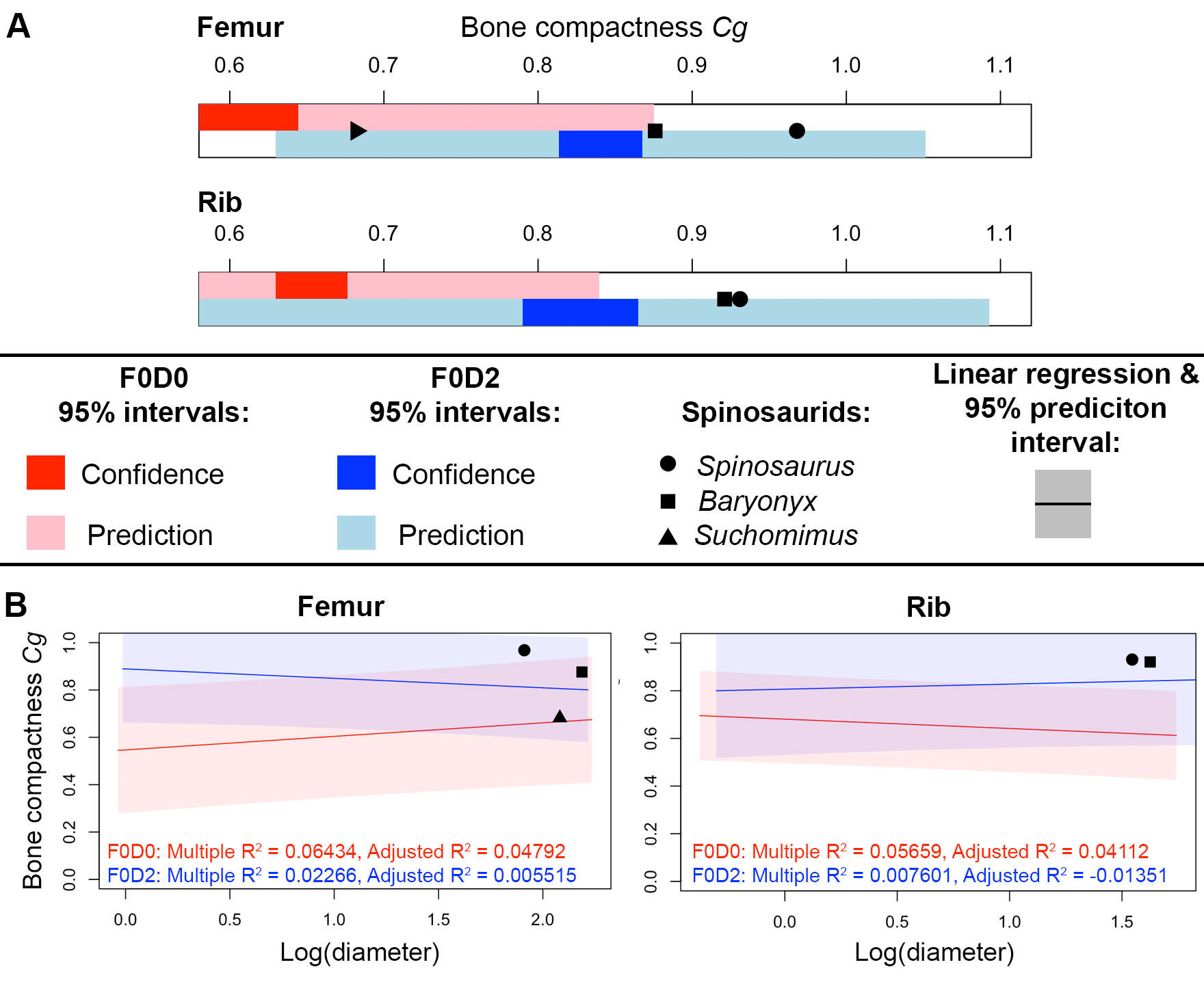

Supplement: S8 Fig — (A) The plots use bars to show the 95% confidence interval and 95% single-prediction intervals for the mean value of Cg in the femoral and rib training sets. Within each training set, the intervals for the F0D0 group are shown as red (95% CI) and pink (95% prediction) bars; the intervals for F0D2 are shown in blue and cyan, respectively. The values for spinosaurid taxa used in Fabbri et al. [15] are marked with solid black markers. The confidence and prediction intervals for the mean provide a simple one-dimensional view of the overlap in distributions between the F0D0 and F0D2 groups. In the femoral dataset, the 95% confidence interval of the mean of F0D2 lies entirely within the prediction interval of F0D0, showing that even the mean Cg in F0D2 would be plausible as a member of F0D0. In the rib dataset, the mean 95% CI for F0D2 is mostly within the prediction interval for F0D0. The 95% CI for the mean of F0D0 overlaps with the prediction interval of F0D2 for femoral data and falls entirely within the interval for rib data. In each case we see that an average value of Cg distribution of one group (say, F0D2 divers) is plausible as a member of the opposite group (the F0D0 nondivers) and vice versa. The overlap in Cg for the groups occurs not only at the edge cases of a group but also extends to group average. (B) Linear regressions (performed without phylogenetic bias adjustment) of (Cg, Log(10, MD)) are plotted with their with 95% prediction interval for the F0D0 and F0D2 groups of femoral and rib datasets. Outputs for R2 from the lm() function in R are reported. The two-dimensional intervals show that the overlap evident in the Cg plots of (A) is also present when diameter is considered. The regression results show that these two-dimensional regressions have extremely weak correlation and have somewhat minor impact on our interpretations, although they often produce F0D0 95% prediction intervals even closer to Spinosaurus values in the bivariate space. The weak co [file pone.0298957.s008.tif]

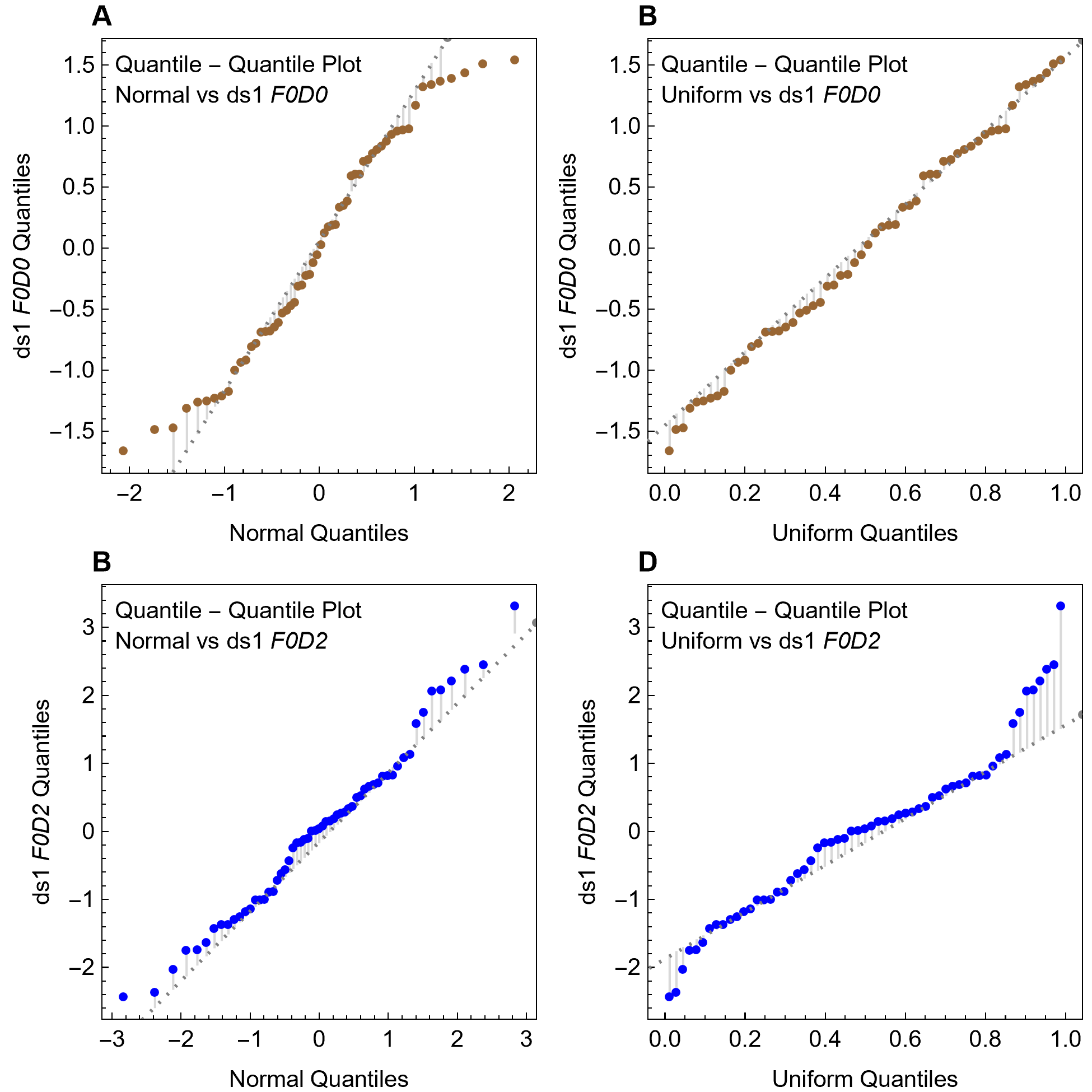

Supplement: S9 Fig — In these panels, the quantiles of the discriminant distributions versus those of a normal or uniform distribution (heavy black points) can be compared to plots of the normal or uniform distribution with itself (thin dotted lines). (TIF) [file pone.0298957.s009.tif]

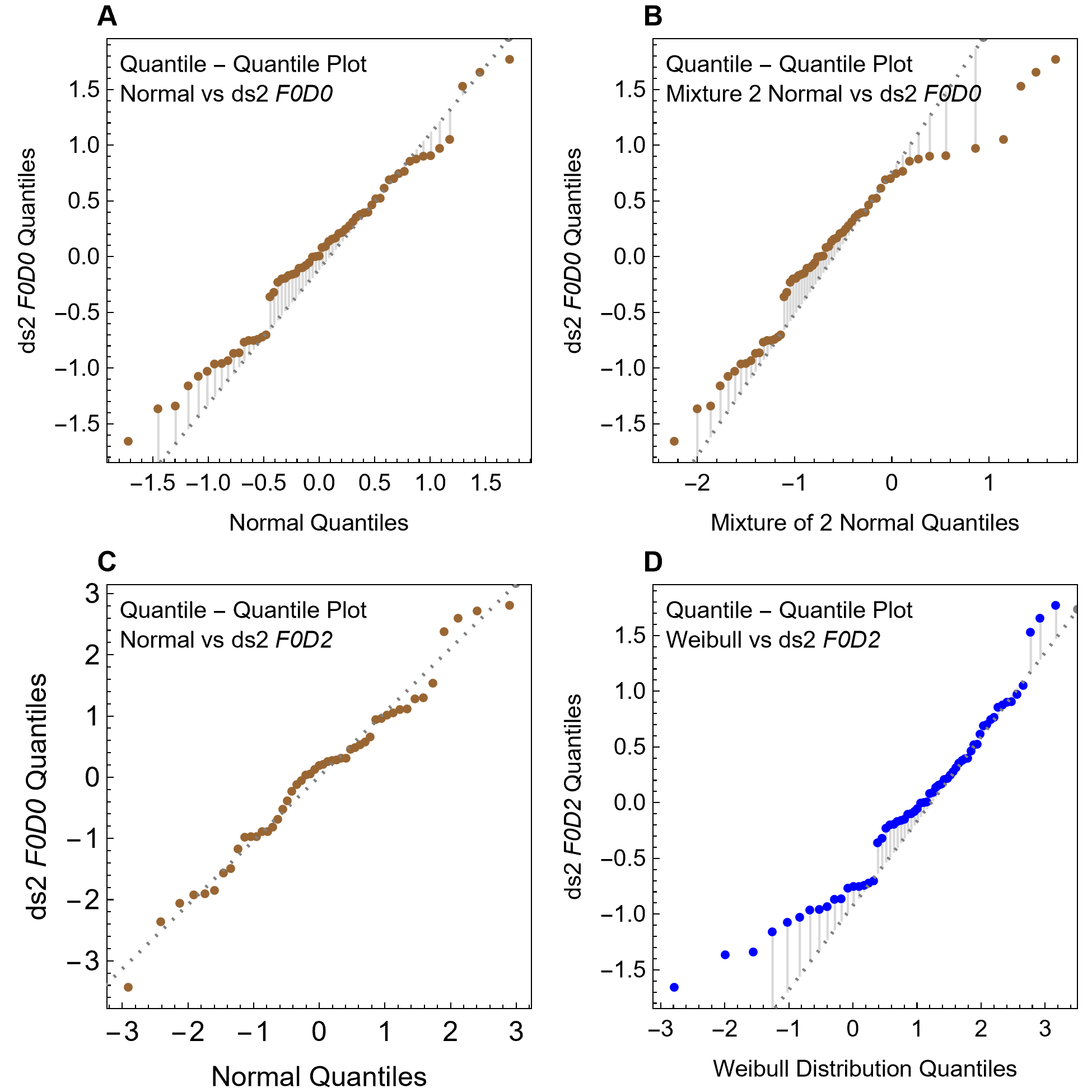

Supplement: S10 Fig — In these panels, the quantiles of the discriminant distributions versus those of a normal or uniform distribution (heavy black points) can be compared to plots of the normal or uniform distribution with itself (thin dotted lines). (TIF) [file pone.0298957.s010.tif]
