# Supplementary material for: Diving dinosaurs? Caveats on the use of bone compactness and pFDA for inferring lifestyle
Source: PLoS One. 2024 Mar 6;19(3):e0298957. doi: 10.1371/journal.pone.0298957 (PMC10917332; doi:10.1371/journal.pone.0298957)
Supplement: S1 Table — Links are provided to Morphosource records containing CT scans created for this study. (DOCX) [file pone.0298957.s011.docx]

S1 Table. Settings for computed-tomographic scans of each of the specimens described.

| Scans conducted at UChicago Hospital medical imaging facility by Nicholas Gruszauskas and David Klein on a Philips Brilliance iCT 256-slice multi-detector CT scanner | | | | | | | | | | | | | | | | |
| --- | --- | --- | --- | --- | --- | --- | --- | --- | --- | --- | --- | --- | --- | --- | --- | --- |
| **Specimen** | **Pixel spacing (mm)** | | **Spacing between slices (mm)** | | | **Exposure** | | **Exposure time (ms)** | | **X-ray tube current (uA)** | | **KVP (kV)** | | **Convolution kernel** | | **Projections** |
| [MNBH GAD500 dorsal vert. 2](https://www.morphosource.org/concern/media/000515705?locale=en) | 0.5104 | | 0.4 | | | 99 | | 1913 | | 52 | | 120 | | YD | | 2038 |
| [FSAC-KK 11888 dorsal vert. 6](https://www.morphosource.org/concern/media/000515710?locale=en) | 0.9766 | | 0.4 | | | 39 | | 1347 | | 29 | | 120 | | YC | | 2613 |
| [FSAC-KK 11888 dorsal vert. 8](https://www.morphosource.org/concern/media/000515714?locale=en) | 0.6510 | | 0.335 | | | 175 | | 1309 | | 134 | | 120 | | YC | | 2734 |
| [MNBH GAD500 dorsal vert. 12](https://www.morphosource.org/concern/media/000515718?locale=en) | 0.5234 | | 0.4 | | | 99 | | 1913 | | 52 | | 120 | | YD | | 2247 |
| [MNBH GAD500 sacral vert. 2 centrum](https://www.morphosource.org/concern/media/000515722?locale=en) | 0.6510 | | 0.335 | | | 175 | | 1309 | | 134 | | 120 | | YC | | 2733 |
| [FSAC-KK 11888 sacral vert. 3 centrum](https://www.morphosource.org/concern/media/000515734?locale=en) | 0.4688 | | 0.4 | | | 99 | | 1913 | | 52 | | 120 | | YD | | 2107 |
| [MNBH GAD500 left femur](https://www.morphosource.org/concern/parent/000434663/media/000434666) | 0.6250 | | 0.335 | | | 200 | | 1309 | | 153 | | 140 | | YC | | 3397 |
| [FSAC-KK 11888 left femur](https://www.morphosource.org/concern/parent/000434657/media/000434660) | 0.4518 | | 0.4 | | | 99 | | 1913 | | 52 | | 120 | | YD | | 2376 |
| [UCRC PV8 right phalanx I-1](https://www.morphosource.org/concern/media/000515745?locale=en) | 0.2747 | | 0.335 | | | 175 | | 1309 | | 134 | | 120 | | YC | | 1492 |
| Scans conducted at the Transportation Safety Board of Canada Engineering Laboratory by Vincent Bolduc and Jordan Mallon on a North Star Imaging X-view X500 CT scanner | | | | | | | | | | | | | | | | |
| **Specimen** | | **Voxel Size (mm)** | | **Focal spot size (um)** | | | **Frame Averaging** | | **Exposure (ms)** | | **Current (uA)** | | **Voltage (kV)** | | **Filter** | **Projections** |
| [CMN 50382 femur](https://www.morphosource.org/concern/media/000517980?locale=en) | | 0.0911 | | 11 | | | 4 | | 250 | | 60 | | 190 | | 1 Cu | 1440 |
| [CMN 41869 partial right femur](https://www.morphosource.org/concern/media/000517988?locale=en) | | 0.0911 | | 0 | | | 2 | | 250 | | 61 | | 190 | | 1 Cu | 3003 |
| Scans used from previously published papers | | | | | | | | | | | | | | | | |
| BSPG-2006-I-54 ant. dorsal vert | | | | | <https://figshare.com/articles/dataset/DICOM_BSPG_2006_I_54/1471654/1> | | | | | | | | | | | |
| NHMUK R 9951 right femur | | | | | <https://www.morphosource.org/concern/parent/000406876/media/000406878> | | | | | | | | | | | |
